# Incorporation of nitrogen in antinutritional *Solanum* alkaloid biosynthesis

**DOI:** 10.1038/s41589-024-01735-w

**Published:** 2024-09-13

**Authors:** Dagny Grzech, Samuel J. Smit, Ryan M. Alam, Marianna Boccia, Yoko Nakamura, Benke Hong, Ranjit Barbole, Sarah Heinicke, Maritta Kunert, Wibke Seibt, Veit Grabe, Lorenzo Caputi, Benjamin R. Lichman, Sarah E. O’Connor, Asaph Aharoni, Prashant D. Sonawane

**Affiliations:** 1 Department of Natural Product Biosynthesis, Max Planck Institute for Chemical Ecology, Jena, Germany; 2 Centre for Novel Agricultural Products, Department of Biology, University of York, York, UK; 3 Department of Plant and Environmental Sciences, Weizmann Institute of Science, Rehovot, Israel; 4 Department of Molecular Ecology, Max Planck Institute for Chemical Ecology, Jena, Germany; 5 Microscopic Imaging Service Group, Max Planck Institute for Chemical Ecology, Jena, Germany

**Keywords:** Biosynthesis, Natural products, Plant sciences, Metabolic pathways, Metabolic engineering

## Abstract

Steroidal glycoalkaloids (SGAs) are specialized metabolites produced by hundreds of *Solanum* species including food crops, such as tomato, potato and eggplant. Unlike true alkaloids, nitrogen is introduced at a late stage of SGA biosynthesis through an unknown transamination reaction. Here, we reveal the mechanism by which GLYCOALKALOID METABOLISM12 (GAME12) directs the biosynthesis of nitrogen-containing steroidal alkaloid aglycone in *Solanum*. We report that GAME12, a neofunctionalized *γ*-aminobutyric acid (GABA) transaminase, undergoes changes in both active site specificity and subcellular localization to switch from its renown and generic activity in core metabolism to function in a specialized metabolic pathway. Moreover, overexpression of *GAME12* alone in engineered *S.* *nigrum* leaves is sufficient for de novo production of nitrogen-containing SGAs. Our results highlight how hijacking a core metabolism GABA shunt enzyme is crucial in numerous *Solanum* species for incorporating a nitrogen to a steroidal-specialized metabolite backbone and form defensive alkaloids.

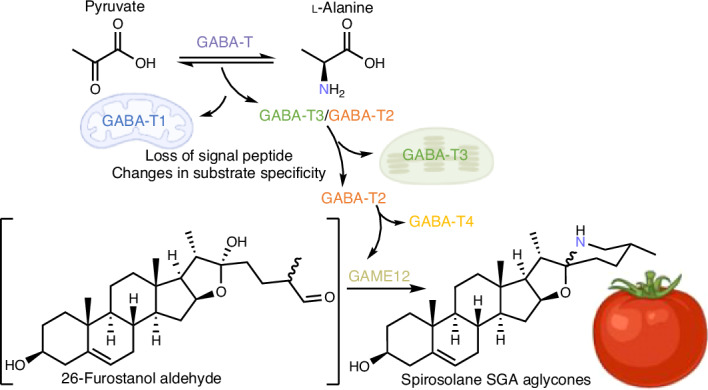

## Abstract (web summary)

The incorporation of nitrogen in steroidal glycoalkaloids is hypothesized to occur through a transamination reaction. Here, the authors show that GLYCOALKALOID METABOLISM12 appears to evolve from the canonical *γ*-aminobutyric acid transaminases and directs the biosynthesis of nitrogen-containing steroidal metabolites in *Solanum* plants.

## Main

**Fig. 1:**
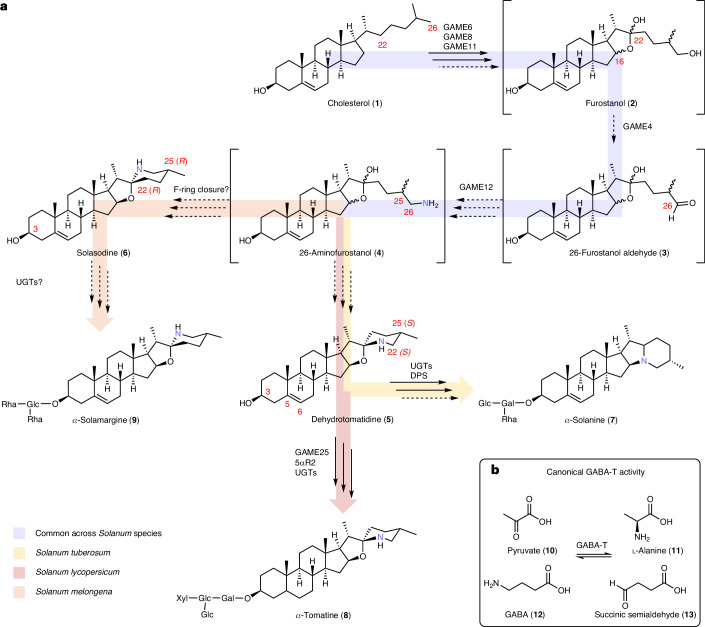
Predicted biosynthetic pathway of SGAs in *Solanum* species*.* **a**, The predicted biosynthetic pathway of SGAs in potato (*S.* *tuberosum*), tomato (*S.* *lycopersicum*) and eggplant (*S.* *melongena*). Solid arrows represent known biosynthetic steps and dashed arrows show uncharacterized steps. Unconfirmed intermediates in the SGA pathway are placed in brackets. Colored arrows represent the branches of SGA biosynthesis specific to different *Solanum* species. The relevant carbon positions modified by the biosynthetic enzymes are numbered in red. **b**, The primary metabolism GABA-T catalyzes pyruvate (**10**) transamination, leading to the formation of alanine (**11**) and succinic semialdehyde (**13**), using GABA (**12**) as a cosubstrate. Gal, galactose; Glc, glucose; Rha, rhamnose; Xyl, xylose; DPS, dioxygenase for potato solanidane synthesis; 5αR2, steroid 5α-reductase 2.

Plants synthesize a huge repertoire of diverse, lineage-specific steroidal-specialized metabolites. Steroidal glycoalkaloids (SGAs) represent one of the major classes of these metabolites, produced by hundreds of wild and cultivated species of the genus *Solanum*, including major staple food crops such as tomato (*S.* *lycopersicum*), potato (*S.* *tuberosum*) and eggplant (*S.* *melongena*)^1^. SGAs have important roles in plant defense and are classified as antinutrients because of their high toxicity and bitterness^2^. SGAs are considered to be pseudoalkaloids, which are compounds that contain a basic nitrogen moiety but, in contrast to true alkaloids, are not derived from an amino acid starting precursor^3^. Instead, SGAs are derived from cholesterol (**1**) and it is proposed that the nitrogen atom is introduced into the cholesterol backbone at a late stage of biosynthesis^4^ (Fig. 1a). After introduction of the nitrogen group, the steroidal alkaloid aglycones (for example, dehydrotomatidine (**5**) in tomato and potato) are subsequently glycosylated by a suite of uridine diphosphate (UDP) glycosyltransferases (UGTs) to generate diverse SGA products (**7**, **8** and **9**)^4–8^.

Itkin et al.^4^ reported on two metabolic gene clusters (on chr7 and chr12) responsible for the biosynthesis of the toxic SGAs *α*-tomatine (**8**) and *α*-solanine (**7**) in tomato and potato, respectively. These SGAs are biosynthesized from the starting precursor cholesterol (**1**) through a series of hydroxylation reactions catalyzed by GLYCOALKALOID METABOLISM6 (GAME6, CYP72A188; at C-22), GAME8 (CYP72A208; at C-26)^4,9^ and GAME11 (dioxygenase; at C-16)^4,10^ enzymes that generate the furostanol-type aglycone scaffold. This putative furostanol (**2**) intermediate is predicted to be oxidized further to the corresponding 26-furostanol aldehyde (**3**) by GAME4, a member of the CYP88D family^4^; however, notably, this C-26 oxidase activity has never been biochemically validated. Subsequently, transamination of 26-furostanol aldehyde (**3**) is mediated by GAME12, a *γ*-aminobutyric acid transaminase (GABA-T)-like protein^4,11^. While *GAME6*, *GAME8* and *GAME11* genes are arranged in a cluster on chr7 together with additional SGA genes (for example, *GAME1* and *GAME17*), downstream *GAME4* and *GAME12* genes are located on chr12 (ref. ^4^). The transamination reaction catalyzed by GAME12 is immediately followed by F-ring closure to generate the nitrogen-containing steroidal alkaloid aglycones dehydrotomatidine (**5**) in tomato and potato and solasodine (**6**) in eggplant (Fig. 1a). The involvement of GAME12 in the introduction of nitrogen in SGA biosynthesis was largely based on silencing experiments performed in tomato and potato. Silencing of *GAME12* and its potato ortholog *PGA4* (*POTATO GLYCOALKALOID BIOSYNTHESIS4*) in tomato and potato plants showed reduced SGA levels, with concomitant accumulation of non-nitrogenous steroidal saponins^4,11^.

Previous reports suggested that the GAME12/PGA4 enzymes belong to the GABA-T protein family^4,11^. Canonical GABA-Ts are involved in the GABA shunt pathway, a bypass of the tricarboxylic acid cycle of core metabolism^12^. In plants, GABA-Ts typically convert GABA (**12**) to succinic semialdehyde (**13**) through a transamination reaction (Fig. 1b)^12,13^. Most plants possess only one GABA-T ortholog, GABA-T1, which is localized to the mitochondria, the site of the GABA shunt pathway^12,13^. Clark et al.^13^ identified three GABA-T homologs in tomato (sharing 75–80% identity at the amino acid level), namely GABA-T1 (Solyc07g043310), GABA-T2 (Solyc12g006470) and GABA-T3 (Solyc12g006450), which showed distinct subcellular localization to the mitochondria, cytosol and plastids, respectively. These enzymes were able to transfer an amino group from GABA (**12**) to pyruvate (**10**), an acceptor substrate, although the mitochondrial GABA-T1 homolog was catalytically more active compared to the cytosolic (GABA-T2) and plastid (GABA-T3) counterparts^13^. GAME12, which is proposed to have a role in tomato SGA biosynthesis, is identical to GABA-T2, the cytosol-localized GABA-T homolog. To date, very few GABA-T family members, for example, *Capsicum annuum* vanillin aminotransferase (VAMT) and *Veratrum californicum* GABA-T1 have been known to catalyze the transamination reactions in specialized metabolism^14,15^. The transamination reaction catalyzed by GAME12 is a critical step that introduces the nitrogen atom into this important class of compounds occurring in hundreds of *Solanum* species^16^. This reaction controls the branch point between the production of antinutritional SGAs and the biologically important non-nitrogenous steroidal saponins^17^.

Here, we demonstrate through biochemical experiments that GAME12 displays the transaminase activity required for SGA biosynthesis. Using in vitro coupled assays with GAME4, also biochemically characterized here, and GAME12, we successfully demonstrate the incorporation of nitrogen and further steroidal alkaloid aglycone formation (for example, solasodine **6**) from the furostanol (**2**) substrate generated in situ. We report that GAME12 is a neofunctionalized GABA-T2 that evolved from a canonical mitochondrial GABA-T, part of the GABA shunt pathway in core metabolism. Comprehensive phylogenomic analyses show that GAME12 emerged in the *Solanum* lineage following three successive gene duplications and under diversifying selection. We moreover identify a fourth GABA-T homolog (named here GABA-T4) that is capable of catalyzing the transamination and subsequent steroid alkaloid aglycone formation from furostanol substrate. Together, our in planta and in vitro results reveal that altered subcellular localization, along with changes in substrate specificity, is crucial for the evolution the specialized aminotransferase enzymes that introduce a nitrogen moiety to form SGAs. Metabolic engineering of *S.* *nigrum* shows that overexpression of *GAME12* leads to de novo production of SGAs in leaves of *S.* *nigrum*, a wild *Solanum* species that typically accumulates only non-nitrogenous steroidal saponins in leaf tissue. This clearly demonstrates that GAME12 controls whether the plant accumulates non-nitrogenous steroidal saponins or nitrogen-containing steroidal alkaloids. Our findings exemplify how hijacking enzymes of core metabolism, a process that can be driven by several evolutionary mechanisms, can lead to neofunctionalized enzymes that in turn generate steroidal-specialized metabolite diversity.

## Results

### Identification and localization of GABA-T homologs

To verify the presence of GABA-T homologs, we first conducted a basic local alignment search tool (BLAST) search using GAME12 as the query against the tomato genome and interestingly identified four (not three as previously reported^13^) GABA-T family members that shared high sequence homology (75–90% identity at the amino acid level) (Supplementary Table 1). This fourth GABA-T homolog (GABA-T4; Solyc08g014610) is most similar to GAME12/GABA-T2 (87% sequence identity at the amino acid level). Like GAME12, GABA-T4 does not contain an N-terminal signal peptide sequence and is, therefore, predicted to be localized in the cytosol (Supplementary Fig. 1).

To experimentally determine the subcellular localization of all four tomato GABA-T homologs, we transiently expressed each of them fused to red fluorescent protein (for example, GABA-T1:RFP, C terminus) in *Nicotiana benthamiana*. Confocal microscopy analysis of the resulting leaf tissues expressing either GAME12/GABA-T2 or GABA-T4 together with a free green fluorescent protein marker (35Spro:GFP:35Ster; GFP, without any target peptide signal) confirmed the cytosolic localization of both GAME12/GABA-T2 and GABA-T4 proteins (Supplementary Fig. 2a,b). In contrast to earlier observations^13^, GABA-T1 appeared to be localized to both the mitochondria and the cytosol (Supplementary Fig. 2c), while GABA-T3 was found exclusively in the plastids (Supplementary Fig. 2d).

The distinct cytosolic localization pattern of GAME12/GABA-T2, along with its significantly lower activity with the substrates involved in the core GABA shunt pathway^13^, strongly suggests that GAME12 is a transaminase specific for the furostanol (**2**) intermediate involved in the proposed SGA biosynthetic pathway. Because the known steps of SGA biosynthesis occur in the cytosol^18,19^, we hypothesized that the loss of the N-terminal localization signal sequence in GAME12 signifies an important evolutionary step toward specialization for SGA production. Therefore, we decided to characterize all GABA-T homologs through both heterologous pathway reconstitution and in vitro biochemical assays.

### Reconstitution of SGA biosynthesis with GABA-T homologs

**Fig. 2:**
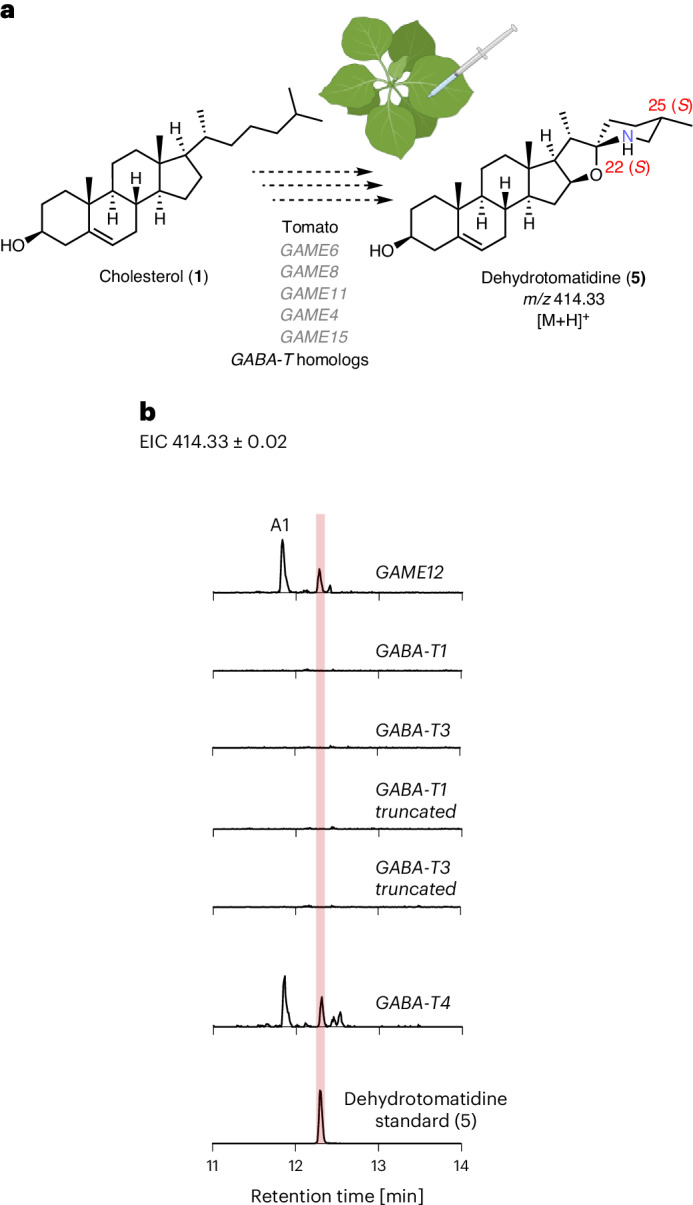
Activity of the tomato GABA-T homologs transiently expressed in *N.* *benthamiana*. **a**, Schematic representation of the pathway reconstitution approach used to assay the GABA-T homologs in *N.* *benthamiana*. **b**, Extracted ion chromatograms (EICs) showing the products of the pathway reconstitution-based assays and the corresponding dehydrotomatidine (**5**) standard. The MS^2^ spectra of the products and the dehydrotomatidine standard (**5**) are presented in Supplementary Fig. 3. The scale is uniform across all chromatograms (the *y* axis signifies signal intensity, where the maximum is 1.6 × 10^4^), except for the dehydrotomatidine (**5**) standard chromatogram where the *y* axis maximum is 1.6 × 10^5^).

*N.* *benthamiana* produces substantial amounts of cholesterol^20^, the starting precursor for SGA biosynthesis. This plant is, therefore, well suited to test the functional activity of SGA biosynthetic enzymes. Each GABA-T homolog was cloned from tomato and transiently coexpressed in *N.* *benthamiana* leaves with the previously reported upstream tomato SGA biosynthetic genes (*GAME6*, *GAME8*, *GAME11* and *GAME4*) and *GAME15* recently identified by us as a component of SGA biosynthesis (Fig. 2a). Metabolic profiling of the infiltrated leaf extracts by ultrahigh-performance liquid chromatography–mass spectrometry (UHPLC–MS) revealed that when GAME12/GABA-T2 was used in the reconstitution assays, dehydrotomatidine (**5**), the first steroidal alkaloid aglycone in tomato, was observed (Fig. 1a, Fig. 2b and Supplementary Fig. 3a,b), along with an additional product (marked as A1 in Fig. 2b) displaying the same mass fragmentation pattern as that of dehydrotomatidine (**5**) (Supplementary Fig. 3c). The characterization of A1 product is described later. We did not observe the formation of dehydrotomatidine (**5**) when GABA-T1 or GABA-T3 was used in place of GAME12/GABA-T2 in this reconstitution-based assay (Fig. 2b). Notably, GABA-T4 was also capable of producing dehydrotomatidine (**5**) in the reconstitution experiments (Fig. 2b). The catalytic activity, along with the cytosolic location, suggests that the GABA-T4 homolog could also have a role in SGA biosynthesis. In contrast to *GAME12* that is primarily expressed in the leaf and flower buds, *GABA-T4* is expressed primarily in root tissues (Supplementary Fig. 4).

We also assayed the cytosolic versions of GABA-T1 and GABA-T3 homologs, named here as GABA-T1 truncated and GABA-T3 truncated, respectively, by eliminating their N-terminal targeting sequences (Supplementary Fig. 5). We speculated that subcellular localization of GABA-T1 and GABA-T3 homologs in the mitochondrial and plastid compartments could be directly preventing their access to cytosolic substrates required for SGA-forming transaminase activity. However, no dehydrotomatidine (**5**) formation was observed when cytosolic or truncated GABA-T1 or GABA-T3 versions, lacking their organelle transit peptide, were expressed together with *GAME15* and upstream SGA pathway genes, suggesting that a cytosolic location is not the only requirement for these GABA-T homologs to act in the SGA pathway (Fig. 2b).

### In vitro GAME12 assays reveal SGA transaminase activity

We next set out to characterize the function of the GAME12 enzyme in vitro. However, the proposed substrate of GAME12, 26-furostanol aldehyde (**3**) (Fig. 1a), is not readily available. This compound has never been observed in any *Solanum* plant and chemical synthesis of it is challenging. Additionally, we did not observe the formation of 26-furostanol aldehyde (**3**) or its proposed immediate precursor furostanol (**2**) in our pathway reconstitution experiments. We, therefore, designed an enzymatic synthesis approach to access furostanol (**2**) and 26-furostanol aldehyde (**3**).

In principle, commercially available saponins such as protodioscin (**14**)^21^ could be hydrolyzed at C-3 and C-26 to yield furostanol (**2**). However, previous studies indicated that acid hydrolysis of furostanol-type saponins results in the formation of spirostane steroidal saponins (closed F-ring), which cannot serve as SGA precursors^11^. Therefore, we tested an enzymatic approach to deglycosylate protodioscin (**14**) under mild conditions^22^.

**Fig. 3:**
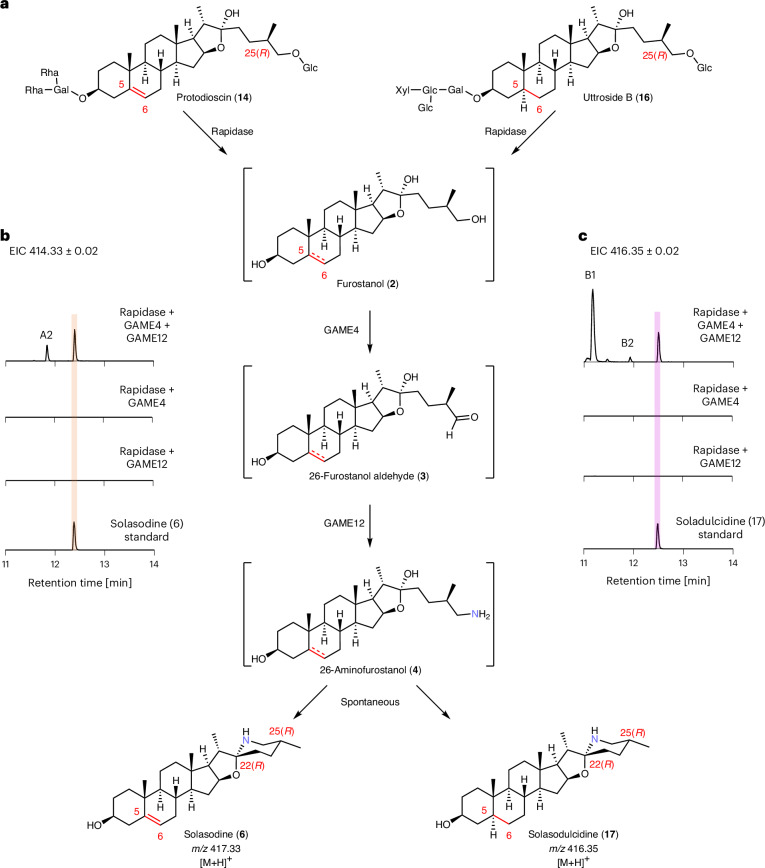
Establishment of in vitro assays of tomato GAME4 and GAME12 leading to the production of steroidal alkaloid aglycones solasodine (6) and soladulcidine (17). **a**, The catalytic steps occurring in the in vitro assays. Both unsaturated (**14**) and saturated (**16**) (C-5,6 bond marked with a dashed red line) steroidal saponins can serve as GAME4 and GAME12 substrates after hydrolysis. **b**, EICs of the in vitro assays products using protodioscin (**14**) as a substrate and the corresponding solasodine (**6**) standard. **c**, EICs of the in vitro assays products using uttroside B (**16**) as a substrate and the corresponding soladulcidine (**17**) standard. The scale is uniform across all of the chromatograms (the *y* axis signifies signal intensity, where the maximum is 1.6 × 10^4^). The MS^2^ spectra of the products and standards are presented in Supplementary Fig. 7.

We first incubated protodioscin (**14**) with Rapidase, a commercially available mix of promiscuous hydrolases, along with microsomes prepared from heterologously expressed *GAME4*, the enzyme that is predicted to oxidize furostanol (**2**) to 26-furostanol aldehyde (**3**), in an overnight reaction (Fig. 3a). After enzymatic incubation, we carried out a reductive amination reaction using dimethylamine and sodium cyanoborohydride. As hypothesized, this reductive amination resulted in the formation a compound with an MS^2^ spectrum consistent with 26-dimethylaminofurostanol (**15**), a tertiary amine that cannot undergo F-ring cyclization (Supplementary Fig. 6)^23^. This assay indirectly confirmed that GAME4 oxidizes furostanol (**2**) to 26-furostanol aldehyde (**3**) in vitro.

We then carried out coupled assays of protodioscin (**14**) using Rapidase and both heterologously produced GAME4 and GAME12 proteins. GABA (**12**) was used as the amino donor for the putative GAME12-catalyzed transamination reaction. We clearly observed the formation of solasodine (**6**), an expected steroidal alkaloid aglycone intermediate in the SGA pathway, thereby confirming the predicted catalytic function of GAME12 (Fig. 3a and Supplementary Fig. 7a,b). Interestingly, we also noticed the formation of another product (A2) with a mass and fragmentation pattern similar to that of the solasodine (**6**) standard (Supplementary Fig. 7c). Upon closer inspection, we observed that the MS^1^ spectrum of product A2 features an ion of *m/z* 432.34, which corresponds to the predicted *m/z* of the 26-aminofurostanol (**4**) intermediate (Supplementary Fig. 7d). Analysis of the coupled in vitro assay extracts by UHPLC–MS using a lower collision energy setting (10 eV) showed that the *m/z* 432.34 ion of product A2 is in fact a parent ion to the *m/z* 414.33 ion, which was, thus, putatively assigned as a 26-aminofurostanol (**4**) (Supplementary Fig. 7e). This was also the case in *N.* *benthamiana* reconstitution experiments where we observed an additional product A1 having same mass and fragmentation as that of dehydrotomatidine (Fig. 2b and Supplementary Fig. 3). On the basis of the MS^1^ spectrum of product A1 that also shows *m/z* 432.34, we tentatively assigned product A1 as a 26-aminofurostanol (Supplementary Fig. 7f). It has been proposed that 26-aminofurostanol (**4**) formed in the GAME12-catalyzed reaction undergoes immediate spontaneous cyclization to form different steroidal aglycone scaffolds (for example, solasodine (**6**) or dehydrotomatidine (**5**); Fig. 1a)^4,11^. To distinguish between products A1 and A2, both of which were putatively assigned as 26-aminofurostanol, we compared the stereochemistry of solasodine and dehydrotomatidine, the final products generated in the corresponding assay systems. Solasodine (**6**), the final product of the in vitro assay, displays 22(*R*),25(*R*) stereochemistry compared to the dehydrotomatidine, the final product of the pathway reconstitution assay (**5**), showing 22(*S*),25(*S*) configurations. Previous labeling studies showed that the C-25 stereochemistry is retained in the final steroidal alkaloid aglycone products after feeding of the cholesterol-derived precursors to different SGA-producing *Solanum* plants^24–26^. These observations suggest that products A1 and A2 are likely the 25-C epimers of 26-aminofurostanol; therefore, we propose product A1 as 25(*S*),26-aminofurostanol and product A2 as 25(*R*),26-aminofurostanol (Supplementary Fig. 8).

To determine whether the putatively assigned 26-aminofurostanol (**4**) intermediate truly cyclizes to steroidal alkaloid aglycones such as dehydrotomatidine (**5**) or solasodine (**6**) and, importantly, whether the cyclization occurs spontaneously or is facilitated by the GAME12 enzyme, we incubated the extracts of in vitro coupled assays and *N.* *benthamiana* reconstitution-based assays at room temperature after protein precipitation. We observed that the peak areas of both products, A1 and A2, were decreased over time with a simultaneous increase in the peak areas of dehydrotomatidine (**5**) and solasodine (**6**), respectively (Supplementary Fig. 8a,b). Therefore, on the basis of previous reports^24–28^, together with our findings and chemical logic, we propose that the F-ring formation occurs through spontaneous cyclization of the respective 26-aminofurostanol (**4**) epimers, leading to the formation of steroidal aglycone scaffolds dehydrotomatidine (**5**) and solasodine (**6**) (Supplementary Fig. 9). We also tested the SGA-forming activity of GAME12 using l-alanine (**11**) as an alternative amino donor (instead of the typical GABA donor) and observed the formation of solasodine (**6**), albeit at lower levels (Supplementary Fig. 10).

Both unsaturated (presence of double bond at C-5,6) and saturated (absence of C-5,6 double bond) SGA types (**7**, **8** and **9**; Fig. 1a) are present across diverse *Solanum* plants^29^. The assays with protodioscin (**14**) led to the formation of unsaturated SGAs. To test the activity of GAME4 and GAME12 on the saturated substrates, we decided to use uttroside B (**16**) as a starting substrate (Fig. 3a). Uttroside B is not commercially available, but a compound with a mass corresponding to uttroside B (**16**) (*m/z* 1197.58) was observed in the leaves of *S.* *nigrum*, a wild *Solanum* species (Supplementary Fig. 11). We isolated this compound from *S.* *nigrum* leaves and confirmed its structure by nuclear magnetic resonance (Supplementary Figs. 12–23 and Supplementary Table 2). This analysis also showed the 25(*R*) configuration in the steroidal scaffold of uttroside B. When uttroside B (**16**) was incubated with Rapidase, GAME4, GAME12 and GABA, we observed the formation of the expected soladulcidine, a saturated SGA product (**17**) (Fig. 3c and Supplementary Fig. 24a,b). We also observed the formation of an additional compound, product B1 (Fig. 3c), with *m/z* 1,034.5533 in the enzyme assays (Supplementary Fig. 24c). The mass fragmentation of product B1 is similar to an authentic standard of α-tomatine (**8**), a glycosylated steroidal alkaloid from tomato having 25(*S*) configuration (Supplementary Fig. 24c,d), but with different retention times (Supplementary Fig. 24e). Presence of the product B1 suggests that GAME4 and GAME12 can also act on partially hydrolyzed uttroside B (**16**) in vitro. Additionally, a trace amount of product B2 with *m/z* 434.36, corresponding to the saturated form of 26-aminofurostanol (**4**), was observed (Supplementary Fig. 24f). As glycosylated saponins with 25(*S*) configuration are not available, we could not investigate the activity of GAME4 and GAME12 for the production of steroidal alkaloid aglycones having the 25(*S*) configuration (for example, dehydrotomatidine (**5**)).

### Phylogenomic analysis of the Solanaceae GABA-T gene family

**Extended Data Fig. 1:**
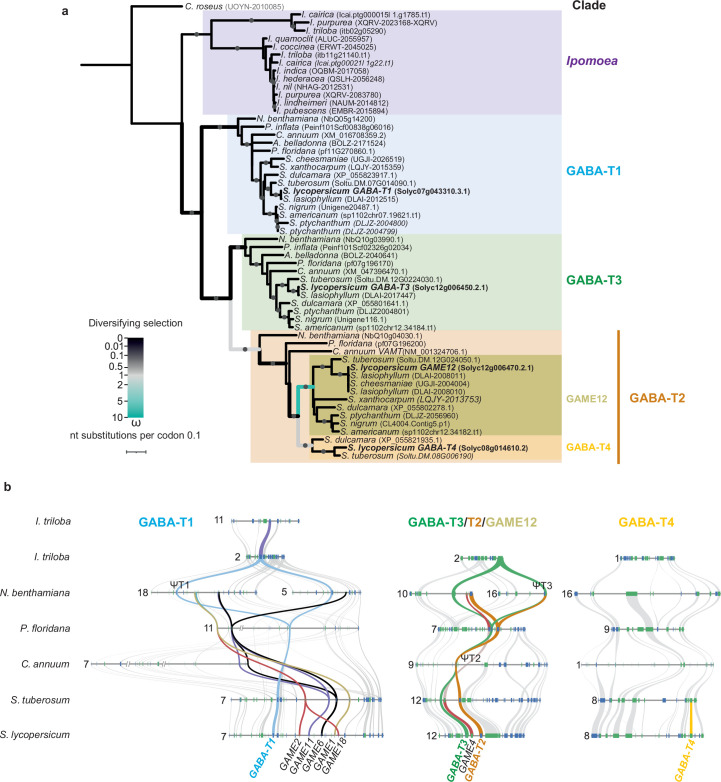
Phylogenomic analysis of GABA-Ts. **a**. Maximum likelihood gene tree of GABA-T homologues. Thick branches show those selected for diversifying selection tests. Branches are coloured if significant selection was detected, with color showing ω_1_ rate class value in model with one rate class per branch. Grey circles show branches with >80% and >95% support as judged by 1000X SH-aLRT and UltraFast Boostrapping replicates, respectively. An extended phylogentic tree can be found in Supplementary Fig. 25. **b**. Microsynteny analysis of GABA-T homologues. Curved lines connecting genomes represent homologous genes, with colors added manually to track genes of interest. Gaps (10-70 Mb) in synteny represented with double slash. Pseudogenes (genes that have premature stop codons in exons, highly divergent sequences or missing expected coding regions. are marked with the Ψ symbol. Larger versions of the results of each syntenic block analysis in panel (**b)** can be found in Supplementary Fig. 26. Source data

With a coupled in vitro assay system in hand, we further aimed to explore the evolutionary origins of the GABA-T homologs in tomato and investigate the molecular basis for the gain of SGA-forming activity by GAME12 and GABA-T4. To examine the genomic and evolutionary origins of the GABA-T homologs, we conducted a phylogenomic analysis, inferring a GABA-T gene tree and tracking the genomic context of GABA-T genes across species using gene order (synteny) analysis (Extended Data Fig. 1). To track the origins of the four homologs in *Solanum*, it was necessary to analyze species across the wider Solanaceae, with *Ipomoea* spp. (morning glory, Convolvulaceae) added as an outgroup. The GABA-T gene tree topology resolved into four major clades: the *Ipomoea* outgroup clade, which includes a lineage-specific duplication, and three Solanaceae clades (GABA-T1, GABA-T2 and GABA-T3) (Extended Data Fig. 1a). The three clades share a GABA-T common ancestor at the base of the Solanaceae. The GABA-T1 clade represents the ancestral-like gene because of its mitochondrial localization and the presence of genes from all examined Solanaceae species. All Solanaceae species examined here also possess additional representative sequences in the chloroplastic GABA-T3 clade (Extended Data Fig. 1a). We could not find a GABA-T2 ortholog in the *Petunia inflata* genome, although we observed *GABA-T2* sequences in the genomes of the non-SGA-producing plants *N.* *benthamiana*, *Physalis floridana* and *C.* *annum*. In the *Solanum* lineage, the GABA-T2 clade is divided into the GAME12 and GABA-T4 subclades (Extended Data Fig. 1a). All *Solanum* species included in the analysis had a *GAME12* ortholog but not all had a *GABA-T4* ortholog. However, the presence of a *S.* *dulcamara* GABA-T4 indicates that the gene may have been lost in some *Solanum* lineages such as *S.* *nigrum*^30^.

To identify the timing of shifts in protein function during the evolution of GABA-T into GAME12, we tested selected branches of the gene tree for diversifying selection. This analysis assesses the balance of silent (synonymous) versus residue-modifying (nonsynonymous) mutations to identify when a protein sequence diversifies more than would be expected. High values of diversifying selection can be observed upon gain of new function following gene duplication^31^. We found that, of nine branches tested, three showed significant diversifying selection: at the origin of the GABA-T2 clade (*P* = 0.0304) and then on both branches following the *Solanum*-specific duplication into the GABA-T4 and GAME12 subclades (*P* = 0.0091 and *P* = 0.0014, respectively) (Extended Data Fig. 1a). The strongest selection was observed on the branch that defines the GAME12 subclade (Supplementary Table 3).

Synteny analysis, conducted only using highly contiguous genome assemblies, allowed us to identify and compare the GABA-T genomic locations. As previously reported^4^, in the *S.* *tuberosum* and *S.* *lycopersicum* genomes, GABA-T1 is found adjacent to the chr7 SGA metabolic gene cluster that contains multiple SGA biosynthetic *GAME* genes. GABA-T3 and GABA-T2 (*GAME12*) are both found on chr12 (Extended Data Fig. 1b). In contrast, we found GABA-T4 homologs inserted into a region with no syntenic connection to the other GABA-T locations. Surprisingly, in *P.* *floridana* and *N.* *benthamiana*, both the chr7 and the chr12 SGA-related syntenic blocks are present, complete with GABA-T and *GAME*-like orthologs. In *C.* *annuum*, the equivalent regions are more diverged, with the chr7 syntenic region lacking GABA-T1 and most of the *GAME* homologs (Extended Data Fig. 1b). The *C.* *annuum* GABA-T3 block contains tandem GABA-T3 and a GABA-T2 pseudogene with no intervening *GAME4*. A second GABA-T2 homolog, which encodes the VAMT previously reported to be involved in capsaicinoid biosynthesis^14,32^, is not present on related syntenic regions. In line with the gene tree, the outgroup *I.* *triloba* has two GABA-T regions, both syntenic to the Solanaceae GABA-T1 and GABA-T3 regions. The deep syntenic connection between these regions was confirmed by the chromosome-level analysis (macrosynteny) (Supplementary Fig. 26d).

### Mutagenesis of GABA-T3 for the gain of SGA-forming activity

**Fig. 4:**
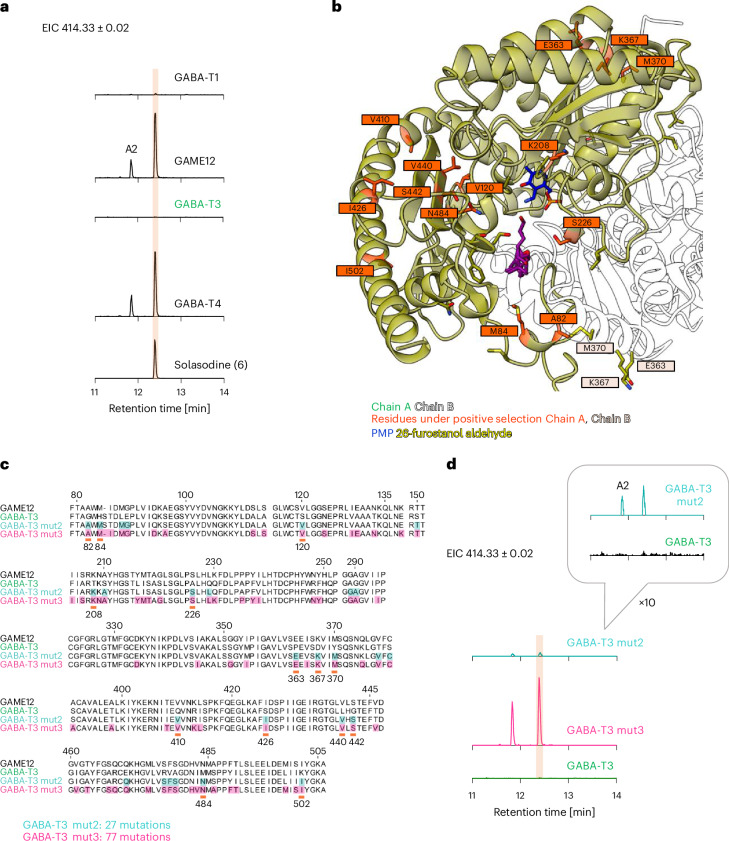
The activity of the tomato GABA-T homologs in in vitro coupled assays and mutagenesis of GABA-T3 for the gain of SGA-forming activity. **a**, EICs of the Rapidase–GAME4-coupled in vitro assays using protodioscin (**14**) as a substrate and the corresponding solasodine (**6**) standard. For the characterization of the additional product A2, a putative 26-aminofurostanol (Supplementary Fig. 7), the concentration of proteins in the assay was normalized to 1 μM of purified protein for all GABA-T homologs. The scale is uniform across all chromatograms. **b**, Visualization of the AlphaFold-generated model of the GAME12 homodimer with 26-furostanol aldehyde (**3**) docked into the active site and the PMP cofactor modeled in using a previously solved crystal structure of a GABA-T (PDB 4ATQ), as described in the Methods^40,41^. The model represents the beginning of the second transamination half-reaction, where the keto acid substrate (26-furostanol aldehyde (**3**)) acts as an amine acceptor. The GAME12 residues corresponding to the codons identified to be under strong diversifying selection are labeled in orange boxes. **c**, Sequence alignment of GAME12, GABA-T3 and GABA-T3 mut2 and mut3. Residue numbering is consistent with the diversifying selection analysis codon numbering. The residues corresponding to the codons identified to be under strong diversifying selection are underlined in orange. The GABA-T3 mut2 and mut3 residues highlighted in color were substituted with the corresponding residues from GAME12. **d**, EICs of the Rapidase–GAME4-coupled in vitro assays of the active GABA-T3 mutants using protodioscin (**14**) as a substrate. The concentration of proteins in the assay was normalized to 1 μM of total protein for all GABA-T homologs. The scale is uniform across all chromatograms of **a** and **d** (the *y* axis signifies signal intensity, where the maximum is 1.6 × 10^4^), except for the ×10 magnification on the chromatogram in **d** marked with a gray box where the *y* axis maximum of 1.6 × 10^3^.

The results of phylogenomics, synteny and diversifying selection analyses encouraged us to verify whether the ancestral GABA-T1 and descendent GABA-T3 show any SGA-forming activity in vitro (without the GABA-T1 or GABA-T3 targeting signal). The coupled in vitro assays were performed using protodioscin (**14**) as a substrate. GABA-T1 showed residual levels of SGA-forming activity in the coupled in vitro assays, while GABA-T3 did not display any solasodine (**6**) formation. In accordance with the results of the pathway reconstitution-based experiments, GABA-T4 showed efficient production of solasodine (**6**) in the in vitro assays (Fig. 4a).

We then set out to pinpoint the changes in the architecture of the GAME12 protein that allowed for the gain of SGA-forming activity. On the basis of the results of the phylogenomics and synteny analysis (Extended Data Fig. 1), we chose GABA-T3 as a background for the gain-of-function mutations, as it shared a more recent common ancestor with GAME12/GABA-T2 than GABA-T1 and did not display any SGA-forming activity in our in vitro assays (Fig. 4a). To identify the residues crucial for the gain of SGA-forming activity by GAME12, we used a combination of protein modeling and substrate docking studies and diversifying selection analysis.

As previously mentioned, three phylogenetic tree branches signifying the emergence of the GABA-T2 and GABA-T3 clades from ancestral GABA-T1 clade and the GAME12 and GABA-T4 subclades from the clade representing Solanaceae GABA-T2s showed significant diversifying selection (Extended Data Fig. 1a). To identify the specific residues corresponding to the codons under strong diversifying selection, we performed BUSTED (branch-site unrestricted statistical test for episodic diversification)^31^ and MEME (mixed-effects model of evolution)^33^ analyses (Fig. 4a, Supplementary Figs. 27 and 28 and Supplementary Table 4). On the basis of the results of diversifying selection analysis, we designed GABA-T3 mut1 (without a target signal), in which all of the residues corresponding to codons under strong diversifying selection were substituted to the corresponding residues from GAME12 (Supplementary Fig. 29). The resulting GABA-T3 mut1 was not expressed efficiently in our heterologous expression system and did not show a gain of activity when tested in vitro (coupled assays with GAME4) using protodioscin (**15**), which suggested that additional changes were crucial for the gain of SGA-forming activity during the emergence of GAME12 from GABA-T3 (Supplementary Fig. 30a,b). Because substitution of the residues under strong diversifying selection was not enough to construct a GABA-T3 SGA-producing mutant, we used AlphaFold-generated models of GAME12 and GABA-T3 with the 26-furostanol aldehyde (**3**) and pyridoxamine phosphate (PMP) cofactor docked in to assess the differences between the active site architecture of the two proteins (Fig. 4b and Supplementary Fig. 30c). It is worth noting that the GAME12 residues found to be under strong diversifying selection are located both within the active site of the protein, as well as on the interface between the two monomers, suggesting that GAME12 underwent substantial structural changes during its emergence from the GABA-T3 ancestor (Fig. 4b and Supplementary Fig. 30c). On the basis of the analysis of the structural models, we designed two additional GABA-T3 mutants, GABA-T3 mut2 and GABA-T3 mut3, which featured additional substitutions of residues found within 8 and 12 Å of the docked-in substrate, as well as substitutions of all the residues under strong diversifying selection (Fig. 4c and Supplementary Figs. 29 and 30d,e). GABA-T3 mut2 containing 27 substitutions in total displayed low levels of SGA-forming activity in vitro despite its poor protein yield in our heterologous expression system (Fig. 4c,d and Supplementary Fig. 30a). The extensively mutated GABA-T3 mut3 (77 substitutions) displayed efficient protein expression and showed SGA-forming activity similar to the GAME12 (Fig. 4c,d).

To assess the impact of protein architecture changes on canonical aminotransferase activity associated with the GABA shunt pathway, we performed in vitro assays with all tomato GABA-T homologs using pyruvate and GABA as cosubstrates. In accordance with previous reports^13^, we observed that GABA-T1 displayed the highest level of l-alanine (**11**)-forming activity, followed by GABA-T3 and GAME12 activity on the canonical substrates (Supplementary Fig. 31). The newly characterized GABA-T4 showed the lowest level of l-alanine (**11**)-forming activity among all of the tomato GABA-T homologs (Supplementary Fig. 31). We also tested the activity of the three GABA-T3 mutants on the canonical substrates and found that all of them displayed only residual levels of alanine (**11**)-forming activity (Supplementary Fig. 31).

### Engineering pseudoalkaloids in *S.nigrum* using GAME12

Because GAME12 controls the branch point between the amino-containing SGAs and steroidal saponins, we hypothesized that we could use it to introduce nitrogen into a saponin backbone and thereby modify the steroidal metabolite profiles in plants. For a proof of concept, we chose to metabolically engineer *S.* *nigrum* plants because the leaves of this plant merely produce steroidal saponins (furostanol-type; for example, uttroside B (**16**)). Moreover, SGAs are only produced in *S.* *nigrum* fruits (also known as berries)^34^. It is still unknown how these two classes of steroidal-specialized metabolites are biosynthesized in *S.* *nigrum* plants from their cholesterol precursor.

**Fig. 5:**
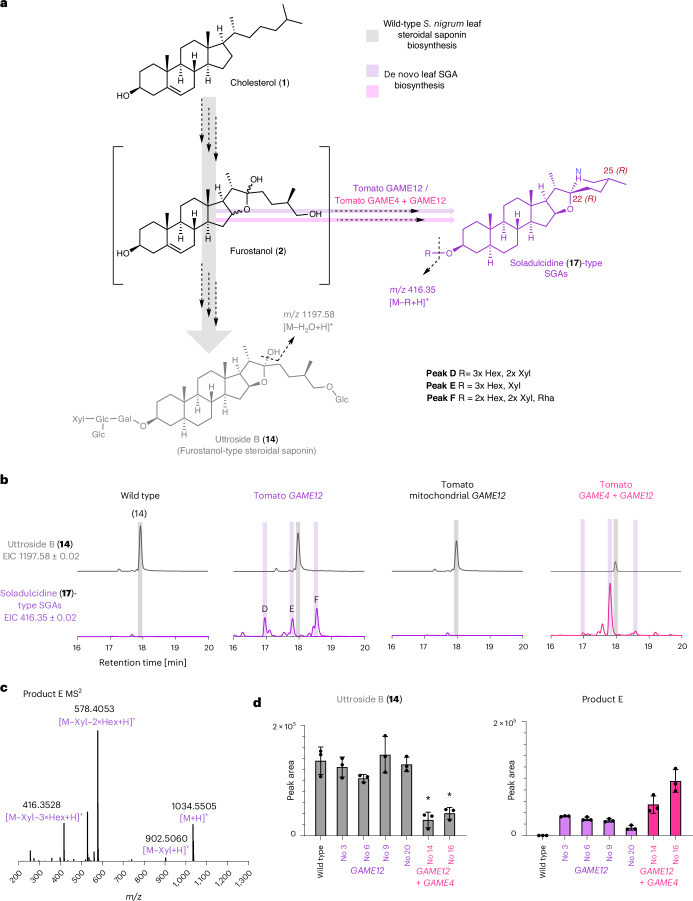
Introducing nitrogen into a steroidal backbone in stably transformed *S.* *nigrum* plants. **a**, Proposed biosynthesis of the most abundant furostane-type steroidal saponin, uttroside B (**16**) in *S.* *nigrum* leaves and the putatively assigned structures of soladulcidine (**17**)-type SGAs extracted from wild-type *S.* *nigrum* green berries. The early biosynthetic steps up to the formation of furostanol (**2**) are hypothesized to be common between the steroidal saponin and SGA pathways in different *Solanum* species. MS^2^-based putative structural assignment of the de novo produced SGAs (peaks D, E and F) can be found in Supplementary Table 5. **b**, EICs from transgenic and wild-type *S.* *nigrum* lines. *S.* *nigrum* leaves stably transformed with GAME12 produce SGAs, as shown by the presence of the characteristic *m*/*z* 416.35, corresponding to steroidal alkaloid aglycone soladulcidine (**17**) observed in UHPLC–MS analysis. The scale is uniform across all chromatograms (the *y* axis signifies signal intensity, where the maximum is 1 × 10^6^) **c**, The MS^2^ spectrum of product E, displaying the ion of *m*/*z* 416.35, characteristic to SGA scaffolds containing a soladulcidine (**17**)-type core. **d**, Comparison of the peak areas of product E, a de novo produced SGA, and the natively produced furostanol-type steroidal saponin uttroside B (**16**) in the wild-type *S.* *nigrum* plants, tomato *GAME12-*overexpressing *S.* *nigrum* transgenic lines (3, 6, 19 and 20) and *S.* *nigrum* lines (14 and 16) stably transformed with tomato *GAME4* and *GAME12*. The bar graphs represent the mean ± s.d. for three biological replicates (*n* = 3). Asterisks signify a statistically significant difference in the area of the uttroside B (**16**) peak between the transgenic lines and wild-type *S.* *nigrum* plants according to an unpaired, two-tailed *t*-test (for line 14, *P = 0.0029; for line 16, *P = 0.038). Source data

The furostanol product generated from cholesterol by the action of GAME6, GAME8 and GAME11 has been proposed as a key intermediate in *Solanum* SGA biosynthesis (Fig. 1a). Further oxidation and transamination of furostanol by GAME4 and GAME12, respectively, generates steroidal alkaloid aglycone scaffolds (for example, solasodine), which are further glycosylated to produce diverse SGA structures (for example, *α*-solamargine) (Fig. 1a). Uttroside B is a major steroidal saponin that accumulates in *S.* *nigrum* leaves^35^ (Fig. 5a and Supplementary Fig. 11). Notably, uttroside B shares a common furostanol scaffold with SGAs (Fig. 5a), strongly suggesting that the furostanol scaffold could act as a branching point for the production of the steroidal saponins in the leaves and SGAs in *S.* *nigrum* berries. This implies that GAME6, GAME8 and GAME11 generating the furostanol intermediate would likely act as common enzymes in both steroidal saponin and SGA biosynthesis in *S.* *nigrum*. Indeed, we explored the *S.* *nigrum* transcriptome (generated in-house from young leaves and green fruits) and identified orthologs of *GAME6*, *GAME8* and *GAME11* genes that were expressed in both young leaves and green fruits (Supplementary Fig. 32a), the tissues with the highest accumulation of steroidal saponins and SGAs, respectively. Although *GAME4*, a furostanol oxidase, was also identified in the transcriptome, it displayed low expression in young leaves as compared to the green fruits of *S.* *nigrum*. Notably, *GAME12* expression could only be detected in the green fruits and not in the young leaves in the transcriptome data (Supplementary Fig. 32a), consistent with the location of SGA accumulation in the same tissue. The expression pattern of these *GAME* genes was further validated by qPCR measurements performed in an independent experiment (five biological replicates per tissue, *n* = 5) (Supplementary Fig. 32b). Consistent with transcriptome data, *GAME12* was only expressed in the green fruits of *S.* *nigrum* (Supplementary Fig. 32b). It is also important to mention that, although *GAME4* was found to be expressed in the leaves in our qPCR experiment, its expression was indeed very low as compared to the upstream *GAME* biosynthetic genes (*GAME6*, *GAME8* and *GAME11*) (Supplementary Fig. 32c). Thus, the absence of *GAME12* expression in *S.* *nigrum* leaves explains the lack of SGAs in this tissue. As orthologs of all *GAME* genes except *GAME12* were expressed in *S.* *nigrum* leaves, this supported our hypothesis that merely overexpression of *GAME12* in this tissue would likely result in de novo production of SGAs.

Notably, expressing the tomato *GAME12* gene driven by a constitutive promoter in *S.* *nigrum* plants resulted in de novo formation of SGAs (Fig. 5b,c and Supplementary Table 5). We further confirmed that the de novo produced SGAs contain soladulcidine (**17**) as a major steroidal aglycone scaffold (25*R*) by analyzing the hydrolysates of leaf extracts from *GAME12*-overexpressed transgenic lines (Supplementary Fig. 33). In contrast, *S.* *nigrum* plants transformed with tomato GABA-T1 or GABA-T3, as well as with their truncated versions, did not result in SGA production in leaves (Supplementary Fig. 34). When we fused the tomato *GAME12* with the GABA-T1 mitochondrial localization signal (N terminus) and transformed to *S.* *nigrum*, no SGAs were observed in the leaf. This observation suggested that cytosolic GAME12 localization is required for SGA biosynthesis in planta (Fig. 5b and Supplementary Fig. 35).

Even though the *S.* *nigrum* lines stably transformed with tomato *GAME12* showed de novo production of SGAs in the leaves, the level of furostanol-type steroidal saponins in these plants did not decrease, meaning that a large portion of the common pathway intermediates were not redirected from steroidal saponin to SGA biosynthesis (Fig. 5b,d). We speculated that weak expression of *GAME4* in *S.* *nigrum* leaves could be limiting the redirection of pathway flux from steroidal saponin to SGA biosynthesis in the *GAME12*-overexpressing transgenic plants. To address this, we created *S.* *nigrum* transgenic plants expressing both tomato *GAME12* and *GAME4* genes, anticipating a boost in the de novo production of SGAs. Indeed, leaves of *S.* *nigrum* plants overexpressing both *GAME4* and *GAME12* displayed a significant increase (~4-fold) in SGA levels, with a simultaneous decrease in the levels of uttroside B, a major steroidal saponin in wild-type leaves (Fig. 5b,d).

## Discussion

The biosynthetic pathway of the antinutritional *Solanum* SGAs has been studied for decades but the crucial transamination step that leads to the formation of the steroidal alkaloid aglycone scaffold was not completely resolved. Although the previously reported changes in metabolite profile that occur upon silencing of *GAME12* suggested the role of this enzyme in SGA biosynthesis^4,11^, biochemical assays of GAME12 have never been possible because of the unavailability of the predicted substrate. We established an in vitro assay system using non-nitrogenous, furostanol-type saponins (for example, protodioscin (**14**)), along with a commercially available hydrolase, and successfully demonstrated the steroidal alkaloid aglycone forming activity of GAME4 and GAME12 enzymes (Fig. 3). We anticipate that the generation of furostanol-type substrates in vitro can also be used to assay many downstream enzymes (for example, UGTs, acyltransferases and CYPs) involved in the formation of the wide variety of steroidal saponins. Moreover, the trapping of 26-furostanol aldehyde (**3**) and detection of 26-aminofurostanol (**4**) in our study demonstrate the long-speculated functions of GAME4 and GAME12 in *Solanum* SGA biosynthesis. Furthermore, our findings illuminate the long-unresolved mechanism of F-ring cyclization in SGA aglycone formation^24–28^.

**Fig. 6:**
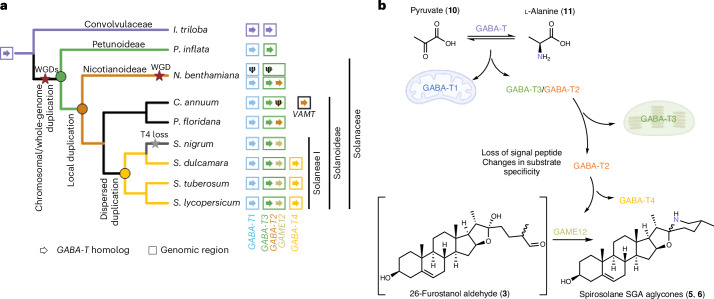
Model of the evolution of the SGA-forming GAME12 enzyme from the canonical GABA-Ts of the Solanaceae*.* **a**, Model of GABA-T evolution in the Solanaceae. GABA-T homologs (arrows) are colored according to the clades in Extended Data Fig. 1a, with the color of the squares matching the syntenic regions in Extended Data Fig. 1b. The square of the *C.* *annuum* VAMT is black, as it is located on an unrelated locus outside of the syntenic regions mentioned in Extended Data Fig. 1b. Selected reported whole-genome duplication events are labeled with a red star and loss of GABA-T4 is labeled with a gray star. **b**, Simplified depiction of the changes at the branch points of the evolution of GABA-T homologs in *S.* *lycopersicum*.

Many recent reports highlighted how core metabolism enzymes can be hijacked for specialized metabolism. Examples include the recruitment of a cellulose-synthase-like protein SOAP5 for triterpene biosynthesis in spinach and the acylsucrose frustofuranosidase invertase in acyl sugar metabolism in *S.* *pennelii*^36,37^. The evolution of novel gene function is closely linked to duplication, as the resulting redundancy can relax constraints and allow sequence divergence^38^. By integrating results from the gene tree, selection and synteny analyses, alongside the localization and biochemical data, it was possible to infer the events that led to the origin of the GAME12 from the primary metabolism GABA shunt pathway GABA-T^11,16^ (Fig. 6).

In the most recent common ancestor of *I.* *triloba* (Convolvulaceae) and the Solanaceae, there was a single GABA-T. Early in the Solanaceae lineage, this GABA-T chromosomal region duplicated to form GABA-T1 and GABA-T3 and their corresponding syntenic blocks (Extended Data Fig. 1 and Fig. 6). This chromosomal duplication was probably caused through whole-genome triplication events known at the base of the Solanaceae^30^. At a common ancestor of *P.* *floridana*, *N.* *benthamiana* and *Solanum*, GABA-T3 underwent a local duplication to give rise to the GABA-T2 clade. Next, in the *Solanum* lineage, a dispersed duplication of GABA-T2 gave rise to the GABA-T4 and GAME12 subclades, triggering protein sequence diversification under selective pressure Extended Data Fig. 1 and Fig. 6). The GABA-T3 local duplication and GABA-T2 dispersed duplication events coincided with a loss of the localization signal peptide resulting in a change in subcellular localization and changes to the enzyme substrate specificity. Both are crucial factors for the SGA-forming activity, as the enzyme must be able to both access and turn over the cholesterol-derived substrates. Moreover, major alterations for the active site pocket and overall protein structure revealed by mutational studies here are likely associated with the neofunctionalization. Thus, the overall evolution of GAME12 from GABA-T represents neofunctionalization, with the ancestor in a different cellular compartment and lacking detectable activity. However, the divergence of the *Solanum* GAME12 from within the GABA-T2 clade appears to be a subfunctionalization, with a cytosolic ancestor likely to have low SGA-forming activity and optimization occurring under selection after duplication. Remarkably, a parallel process described in a recent publication occurred in the *C.* *annum* lineage, with a dispersed GABA-T2 duplication giving rise to new function: the VAMT responsible for capsaicin production^39^ (Fig. 6). This pattern of divergence following duplication hints at an ancestral function of GABA-T2 (or GABA-T4) outside the SGA pathway. Such a function is yet unknown, although the loss of GABA-T4 paralogs in *Solanum* spp. indicates that it is nonessential.

The de novo production of SGAs in tomato *GAME12*-expressing *S.* *nigrum* leaves highlights that transamination is the branch point between steroidal saponin and SGA pathways in *Solanum* plants. The significant increase in SGA levels in *S.* *nigrum* lines expressing both *GAME4* and *GAME12* further substantiate the crucial role of the GAME4 oxidase in the SGA biosynthetic pathway. These results clearly show that it is possible to introduce a nitrogen into steroidal backbone and generate pseudoalkaloid-producing plants. In summary, characterization of the crucial enzymes involved in the biosynthesis of the SGA core scaffold allow the engineering of a dramatic switch in steroidal product profiles in plants. This study, therefore, sets the stage for metabolic engineering efforts to improve access to this valuable and diverse class of metabolites.

## Methods

### Chemicals and solvents

All the solvents used for UHPLC–MS analysis were MS-grade and were purchased from Thermo Fisher Scientific. Solvents used for semipreparative HPLC were HPLC-grade and purchased from Thermo Fisher Scientific. Ethanol and methanol used for in vitro assay extractions and extractions from *S.* *nigrum* and *N.* *benthamiana* tissues were HPLC-grade and purchased from Sigma-Aldrich (Supelco). Protodioscin, solasodine, tomatidine and l-alanine were purchased from Sigma-Aldrich. Dehydrotomatidine is present as an impurity in the tomatidine standard. Soladulcidine was purchased from Toronto Research Chemicals. All of the restriction enzymes were purchased from New England Biolabs. The Rapidase hydrolase mix (DSM Food Specialties) was purchased from Max F. Keller. Ready-to-use buffers were purchased from Sigma-Aldrich. Rifampicin and kanamycin were purchased from Sigma-Aldrich; spectinomycin, carbenicillin and gentamicin were purchased from Thermo Fisher Scientific.

### Cloning of the GABA-T homologs for in planta expression

Cultivated tomato (*S.* *lycopersicum* cv. Micro Tom) complementary DNA (cDNA) was prepared from previously isolated RNA using the high-capacity cDNA reverse transcription kit (Applied Biosystems). GABA-T candidates were identified by BLAST search against the SolGenomics database^42^. All primers used in this study were purchased from Sigma-Aldrich. Tomato GABA-T1, GABA-T3, *GAME12* (GABA-T2), GABA-T1 truncated, GABA-T3 truncated and core SGA pathway genes, *GAME6*, *GAME8*, *GAME11*, *GAME4* and *GAME15*, were amplified using tomato leaf cDNA as a template. GABA-T4 was amplified from a synthetic gene (Twist Bioscience). The amplification was carried out using either Phusion high-fidelity DNA polymerase (New England Biolabs) or Platinum SuperFi polymerase (Thermo Fisher Scientific). The sequences of all the primers used for PCR amplifications can be found in Supplementary Table 7. The resulting amplicons were cloned into a *BsaI-*digested modified 3Ω1 vector^43^ harboring a spectinomycin resistance gene, using the InFusion cloning kit (Clontech Takara). The resulting plasmids were transformed into chemically competent *Escherichia coli* Top10 cells (Invitrogen). Transformed cells were spread and selected for on Luria–Bertani agar plates supplemented with 100 μg ml^−1^ spectinomycin. The resulting colonies were used to inoculate liquid LB cultures, further used for plasmid isolation. Plasmids were isolated from the positive colonies using the Wizard Plus SV minipreps DNA purification kit (Promega). The sequence of the introduced fragments was verified using the Sanger sequencing service provided by Genewiz (Azenta Life Sciences). The verified plasmids were transformed into electrocompetent *Agrobacterium tumefaciens* GV3101 cells using electroporation. Transformed cells were spread and selected for on LB agar plates supplemented with 50 μg ml^−1^ rifampicin, 50 μg ml^−1^ gentamicin and 200 μg ml^−1^ spectinomycin. The presence of the desired coding sequence in the vector was verified by colony PCR. Briefly, the positive *A.* *tumefaciens* colonies were picked and dipped in 8 μl of double-distilled water. The suspension was mixed with the Phire II Master Mix (Thermo Fisher Scientific) and appropriate primers. The size of the resulting amplicons was verified using agarose gel electrophoresis. All GAMEs and GABA-T coding and amino acid sequences are provided in Supplementary Dataset 1.

### Testing GABA-Ts using in planta pathway reconstitution

The positive *A.* *tumefaciens* colonies were used to inoculate bacterial cultures in LB Lennox medium supplemented with 50 μg ml^−1^ rifampicin, 50 μg ml^−1^ gentamicin and 200 μg ml^−1^ spectinomycin. Cultures were then grown overnight at 28 °C with 220 r.p.m. shaking. The bacterial cells were then pelleted by centrifugation and resuspended in 10 ml of the infiltration buffer (50 mM MES pH 5.6, 10 mM MgCl_2_, 200 μM acetosyringione and 50 mg ml^−1^ d-glucose). The optical density at 600 nm (OD_600_) of the cell suspensions was measured and the appropriate volumes of each strain to mix within the final inoculum were calculated. The appropriate strains were mixed and the OD_600_ of the final inoculum was adjusted to 0.6. The mixes were incubated at room temperature with shaking in darkness for 2 h. *N.* *benthamiana* plants were grown in low-nutrient F1 compost under a long-day photoperiod (16 h light, 8 h dark) at 22 °C, 40–65% relative humidity for 3 weeks in a greenhouse. Plants were transferred to a controlled-environment chamber (York, Johnson Controls), operating under the same conditions. The *N.* *benthamiana* leaves were infiltrated with the *A.* *tumefaciens* mixtures from the abaxial site, using a needleless syringe. The plants were then incubated in the controlled-environment chamber for a further 96 h before being collected. Infiltrated tissue was isolated, snap-frozen in liquid N_2_ and stored at −80 °C until further analysis. Metabolic extraction was performed using 100% LC–MS-grade ethanol. Frozen leaf tissue was pulverized using a micropestle. For 100 mg of weighed-out frozen tissue, 300 μl of ethanol was added. The resulting mixture was then briefly vortexed and sonicated in a sonic bath for 15 min, further centrifuged at full speed for 15 min and passed through a 0.45-μm PTFE centrifuge filter (Thermo Fisher Scientific). The resulting extracts were transferred to LC–MS vials and subjected to UHPLC–MS analysis.

### Subcellular localization of GABA-Ts in *N.benthamiana*

All four tomato GABA-T homologs C-terminally fused to RFP were coexpressed with free GFP or mitochondrially targeted GFP^44^, using *Agrobacterium*-mediated transient expression in *N.* *benthamiana*, following the same procedure as in the pathway reconstitution experiments, with the only difference being the lower OD_600_ of the final inoculum (OD_600_ = 0.3). Leaf discs (0.5 cm in diameter) were isolated from the leaves 48 h after infiltration. Micrographs of the freshly punched leaf discs mounted in water were acquired on a cLSM 880 (Zeiss) using a Plan-Apochromat ×20/0.8 air or a C-Apochromat ×40/1.20 water immersion objective. Used excitation light sources were a 405-nm laser diode (3% transmission), 488-nm argon laser (4%) and a 543-nm helium–neon laser (30%) for chlorophyll autofluorescence, GFP and RFP respectively. The transmitted light was captured with a T-PMT added in the GFP channel. Two sequential tracks were acquired to reduce crosstalk between GFP and RFP. The spectral detector was set to an emission detection range of 490–570 nm in combination with a 488 MBS for GFP in the first track and 580–650 nm with a 488/543 MBS for RFP and 670–735 nm for chlorophyll in a second track. Acquisition was controlled in ZEN (Zeiss) and executed unidirectionally with 1 Airy unit, frame averaging of 4 and approximately 0.5 µs per pixel dwell time. Contrast improvement, cropping and scale bar insertion were performed in ImageJ^45^.

### UHPLC–MS analysis of *N.benthamiana* extracts

Extracts were analyzed using a Thermo UltiMate 3000 UHPLC system (Thermo Fisher Scientific) coupled with an Impact II ultrahigh-resolution quadrupole time-of-flight (QTOF) MS instrument (Bruker) and the Bruker Compass qToF Control version 6.3 and Bruker Compass Hystar version 6.0.30.0. software. Separation was performed on a Waters Acquity Premier BEH C18 Vanguard FIT column (2.1 × 100 mm, 1.7 μm, 130 Å) at column temperature of 40 °C. The first minute of each run was redirected to waste. Solvent A was 0.1% formic acid in water; solvent B was 100% acetonitrile. The gradient was as follows: 5% B from 0 to 1 min, to 28% B at 9.5 min, to 90% B at 14 min, held at 90% B until 16 min and then 100% B at 18 min. The column was then flushed with 100% B for 2 min and re-equilibrated to 5% B for 2.5 min. The flow was 0.3 ml min^−1^ and the injection volume was 2 μl. The samples were ionized using a pneumatic-assisted electrospray ionization source in positive mode (ESI^+^) with a capillary voltage of 3,500 V, plate offset of 500 V, nebulizer gas pressure of 2.0 bar, drying gas flow of 10 L min^−1^ and drying temperature of 250 °C. The spectra were acquired at a 12-Hz rate within the 80–1,300 *m/z* scan range. Fragmentation was triggered at an absolute threshold of 400 counts and limited to a total cycle time of 0.5 s. The stepping mode (20 to 50 eV) was used for the collision energy. Sodium formate solution in isopropanol was injected at the beginning of each run and the *m/z* values were recalibrated using the expected calibrant ion *m/z* values. Acquired data were analyzed using the Bruker DataAnalysis version 5.3 software and MzMine version 3.4.27 software^46^. The dehydrotomatidine (**5**) product was identified on the basis of the presence of previously reported fragment ions characteristic for SGA aglycones and correspondence to the spectrum of the dehydrotomatidine (**5**) standard^4,47^.

### *GAME4* cloning and microsome preparation

The tomato *GAME4* coding sequence was amplified from the previously obtained in planta expression plasmid (3Ω1) using Platinum SuperFi polymerase (Thermo Fisher Scientific). The resulting amplicons were cloned into a SalI*-*digested and Antarctic phosphatase-treated (New England Biolabs) pESC-Trp vector (Agilent), using the InFusion cloning kit (Clontech Takara). The resulting plasmids were transformed into chemically competent *E.* *coli* Top10 cells (Invitrogen). Transformed cells were spread and selected for on LB agar plates supplemented with 100 μg ml^−1^ carbenicillin. The resulting colonies were used to inoculate liquid LB cultures, further used for plasmid isolation. Plasmids were isolated from the positive colonies using the Wizard Plus SV minipreps DNA purification kit (Promega). The sequence of the introduced fragments was verified using the Sanger sequencing service provided by Genewiz (Azenta Life Sciences). The verified plasmid was transformed into chemically competent WAT11 *Saccharomyces cerevisiae* cells, according to a previously described protocol^48^. Transformed cells were spread and selected for on SD-Trp dropout plates supplemented with 2% (v/v) glucose. The presence of the *GAME4* coding sequence in the vector was verified by colony PCR. Briefly, the positive *S.* *cerevisiae* colonies were picked and dipped in 100 μl of 20 mM NaOH and incubated at 98 °C for 10 min. The suspension was briefly centrifuged and 2 μl was mixed with the Phire II Master Mix (Thermo Fisher Scientific) and appropriate primers. The size of the resulting amplicons was verified using agarose gel electrophoresis. Verified colonies were used to inoculate 30-ml cultures of SD-Trp dropout medium supplemented with 2% (v/v) glucose, which were incubated overnight at 30 °C with 200 r.p.m. shaking. The overnight cultures were used to start 100-ml SD-Trp dropout cultures supplemented with 2% (v/v) glucose, which were incubated at 30 °C with 200 r.p.m. shaking for another 24 h. The cells were pelleted by centrifugation, resuspended in 100 ml of SD-Trp dropout medium supplemented with 2% (v/v) galactose. The cultures were subsequently incubated for 18 h at 30 °C with 200 r.p.m. shaking. Cells were then pelleted by centrifugation at 4 °C, resuspended in 15 ml of TEK buffer (50 mM Tris-HCl, 1 mM EDTA and 0.1 M KCl), mixed and then pelleted by centrifugation. The pellets were resuspended in 2 ml of TES buffer (50 mM Tris-HCl, 1 mM EDTA and 0.6 M sorbitol) and lysed with glass beads. The beads were washed with 5 ml of TES buffer and the resulting suspension was collected, transferred to a centrifuge tube and centrifuged at 7,500*g* for 10 min at 4 °C. The resulting supernatant was transferred to ultracentrifuge tubes and centrifuged for 90 min at 100,000*g*. The supernatant was discarded and the microsomal pellet was washed first with TES buffer and then with TEG buffer (50 mM Tris-HCl, 1 mM EDTA and 20% (v/v) glycerol). The resulting microsomes were transferred to a potter, homogenized, aliquoted, snap-frozen in liquid N_2_ and stored at −80 °C until further use_._

### Trapping 26-furostanol aldehyde (3) through reductive amination

In vitro assays of GAME4 oxidase were performed in 50 mM HEPES buffer (pH 7.5), using 500 μg of protodioscin (**14**) substrate, 18 mg of Rapidase, 80 μl of GAME4 microsomes and 4 mM reduced nicotinamide adenine dinucleotide phosphate (NADPH) in a total volume of 500 μl overnight. The assay was then extracted three times with ethanol acetate (1 ml) and the combined organic phases were sequentially dried over anhydrous Na_2_SO_4_ and concentrated under reduced pressure. To a separate 10-ml amber glass vial was added anhydrous methanol (0.5 ml), dimethylamine hydrochloride (5.2 mg, 64 µmol) and triethylamine (8 µl, 64 µmol) under an Ar atmosphere. The mixture was then charged with the previously prepared extract containing 26-furostanol aldehyde (**3**) (approximately 0.5 mg, 1.2 µmol) in anhydrous methanol (1 ml). After stirring for 15 min at room temperature, NaCNBH_3_ (4 mg, 58 µmol) was added in one portion and the resulting solution was stirred at room temperature for a further 24 h. The reaction mass was then treated with H_2_O (2 ml) and subsequently extracted three times with CHCl_3_ (1 ml). The combined organic phases were dried over anhydrous Na_2_SO_4_ and concentrated in vacuo to afford an opaque residue. Analysis of the crude material indicated the presence of title compound dimethylaminofurostanol (**15**): retention time, 12.03 min; HRMS (ESI-QTOF) *m/z*, [M + H]^+^ calculated for C_29_H_50_NO_3_, 460.3785; found, 460.3788.

### Cloning, expression and purification of GABA-T homologs

Tomato GABA-T homolog coding sequences were amplified from the previously obtained in planta expression plasmids using Platinum SuperFi polymerase (Thermo Fisher Scientific). The resulting amplicons were cloned into a KpnI–HindIII-digested pOPINF, using the InFusion cloning kit (Clontech Takara)^49^. The resulting plasmids were transformed into chemically competent *E.* *coli* Top10 cells (Invitrogen). Transformed cells were spread and selected for on LB agar plates supplemented with 100 μg ml^−1^ carbenicillin. The resulting colonies were used to inoculate liquid LB cultures, further used for plasmid isolation. Plasmids were isolated from the positive colonies using the Wizard Plus SV minipreps DNA Purification kit (Promega). The sequence of the introduced fragments was verified using the Sanger sequencing service provided by Genewiz (Azenta Life Sciences). The pOPINF vectors containing the GABA-T coding sequences were transformed into chemically competent BL21 *E.* *coli* cells. The cells were spread on LB agar plates supplemented with 100 μg ml^−1^ carbenicillin. The transformed colonies were verified using colony PCR. Briefly, the positive colonies were picked and dipped in 8 μl of double-distilled water. The suspension was then mixed with the Phire II Master Mix (Thermo Fisher Scientific) and appropriate primers. The size of the resulting amplicons was verified using agarose gel electrophoresis. Positive colonies were used to inoculate 1-ml LB Lennox cultures supplemented with 100 μg ml^−1^ carbenicillin. The cultures were incubated at 37 °C overnight with 220 r.p.m. shaking. The cultures were then diluted in 100 ml of 2× YT medium, supplemented with 100 μg ml^−1^ carbenicillin. The cultures were grown at 37 °C with 220 r.p.m. shaking until the culture OD_600_ of 0.6–0.8 was reached. The cultures were then transferred to an 18 °C incubator and protein expression was then induced using 200 μM IPTG. The cultures were incubated at 18 °C with 220 r.p.m. shaking for a further 16 h. For protein purification, the cells were pelleted by centrifugation at 4 °C for 15 min at 3,200*g*. Each pellet was resuspended in 10 ml of buffer A1 (50 mM Tris-HCl, 50 mM glycine, 500 mM NaCl, 20 mM imidazole and 5% (v/v) glycerol, pH 8.0) with 0.2 mg ml^−1^ lysozyme and EDTA-free protease inhibitor tablets (Roche). Cells were lysed by sonication using a Sonics Vibra Cell VCX 750 system (Sonics and Materials), at 40% amplitude, in a 1-s on, 2-s off cycle for a total of 4 min. The lysates were then centrifuged at 4 °C for 15 min at 35,000*g*. The centrifuged lysates were transferred to 15-ml Falcon tubes and incubated for 1.5 h at 4 °C with 500 μl of NiNTA-agarose beads (Takara), which were equilibrated in A1 buffer before usage. The resulting mixtures were then passed through A1-primed 14-ml Protino columns (Macherey-Nagel). The beads sedimented at the bottom of the columns were washed twice with 10 ml of A1 buffer. The protein was eluted from the beads with 2.5 ml of B1 buffer (50 mM Tris-HCl, 50 mM glycine, 500 mM NaCl, 250 mM imidazole and 5% (v/v) glycerol, pH 8.0). The eluates were dialyzed using X30 PD10 desalting columns (Cytiva) and eluted in buffer A4 (20 mM HEPES, 150 mM NaCl and 10% (v/v) glycerol, pH 7.5). Proteins were then concentrated using Amicon 10-kDa size-exclusion concentrators (Millipore). Protein concentration was measured using the Pierce Rapid Gold BCA protein assay kit (Thermo Fisher Scientific), according to the manufacturer’s instructions.

### In vitro SGA-formation activity assays

Coupled in vitro assays of Rapidase (DSM Food Specialties), GAME4 microsomal preparation and GABA-T homologs were carried out as follows: reaction mixtures were set up to contain 50 mM HEPES buffer pH 7.5, 50 μM furostanol-type saponin substrate (protodioscin (**14**) or uttroside B (**16**)), 100 μM pyridoxal phosphate (PLP), 5 mM GABA or 5 mM l-alanine, 500 μM NADPH, 2.4 μg μl^−1^ Rapidase, 5 μl of microsomes and 2 μM GABA-T protein. The total reaction volume was 100 μl. The reactions were incubated at 30 °C for 3 h with 750 r.p.m. shaking. The assay mixtures were then extracted with 1× assay volume of 100% ethanol, vortexed briefly and centrifuged at full speed for 20 min to precipitate the proteins. The resulting mixture was analyzed using UHPLC–MS as described above. The SGA products were identified on the basis of the presence of previously reported fragment ions and correspondence to the spectra of the standards^4,47^.

### Structural characterization of uttroside B (16)

Uttroside B (**16**) was purified from wild-type *S.* *nigrum* leaf tissue. *S.* *nigrum* leaves were collected and frozen in liquid N_2_. Frozen tissue was pulverized using a blender and 70 g of powder was weighed out. The tissue was mixed with 500 ml of 100% methanol and filtered through Miracloth (Millipore); the remaining solvent was evaporated under reduced pressure. The resulting residue was resuspended in 20 ml of methanol and passed through a glass syringe filter. The eluate was fractionated using an Agilent 1260 Infinity semipreparative HPLC system. Separation was performed on a Phenomenex Kinetex 5-μm XB-C18 (4.6 × 100 mm, 5 μm, 100 Å) column. The column temperature was 40 °C. Solvent A was 0.1% formic acid in water; solvent B was 100% acetonitrile. The gradient was as follows: 5% B from 0 to 1 min, to 28% B at 11.50 min, to 100% B at 18.50 min and held at 100% B until 20 min. The column was re-equilibrated to 5% B from 20.5 min to 23 min. The injection volume was 10 μl. The flow rate was 1.5 ml min^−1^. Eluted fractions were collected at 30-s intervals. Each fraction was analyzed using the UHPLC–MS method described above to check for the presence of *m/z* 1,197.58 [M + H-H_2_O]^+^ ions, as uttroside B is not ultraviolet active. The collected fractions containing uttroside B were pooled and dried under reduced pressure. NMR measurements were carried out on a 500-MHz Bruker Avance III HD spectrometer (Bruker Biospin), equipped with a TCI cryoprobe using standard pulse sequences as implemented in Bruker Topspin version 3.6.1. (Bruker Biospin). Chemical shifts were referenced to the residual solvent signals of pyridine-*d5* (*δ*_H_ 8.74, *δ*_C_ 150.35). The structure of uttroside B (**16**) was determined by one-dimensional and two-dimensional NMR spectroscopy (^1^H, distortionless enhancement by polarization transfer including the detection of quaternary nuclei, correlation spectroscopy, heteronuclear single quantum coherence (HSQC), heteronuclear multiple bond correlation, HSQC total correlation spectroscopy and rotating-frame nuclear Overhauser enhancement; Supplementary Figs. 11–23). The NMR data (Supplementary Table 2) confirmed that the steroidal saponin is uttroside B with a (25*R)* configuration. The spatial configuration of position 25 was deduced from the chemical shift difference of the neighboring position 26 (*δ*_H_(26a) − *δ*_C_(26b) = 0.32 ppm). This difference had a value of >0.57 ppm for the (25*S*) configuration and <0.48 ppm for the 25(*R*) configuration^50,51^. Our NMR data of uttroside B are in good agreement with reported NMR data of the structurally related borivilianoside C (known as uttroside A, where C-22 is substituted with OCH_3_ instead of OH)^52^.

### Comparative genomic analyses of GABA-T orthologs

Comparative genomics was performed using the chromosome-level genome assemblies of *C.* *annuum*^53^, *I.* *triloba*^54^, *N.* *benthamiana*^55^, *P.* *floridana*^56^, *S.* *lycopersicum*^57^ and *S.* *tuberosum*^58^. Coding sequences were extracted with AGAT^59^ using genome sequences and corresponding GFF annotation files. Orthogroups and a species tree were inferred with Orthofinder^60^ using nucleotide sequences. Orthogroup sequences were aligned with MAFFT L-INS-i and maximum-likelihood trees inferred using IQ-Tree 2 (ref. ^61^) with ModelFinder^62^, 1,000 bootstraps (UFBoot2)^63^ and 1,000 SH-aLRT supports^64^. The JCVI implementation^65^ of MCScan^66^ was used for macrosynteny and microsynteny analyses using the nucleotide sequences of primary isoforms. For macrosynteny, the default settings and a minimum span (‘--minspan’ flag) of 16 were used. Pairwise microsynteny with MCScan was anchored to *S.* *lycopersicum* with a maximum of six iterations.

### GABA-T phylogeny

GABA-T ortholog gene sequences are provided in Extended Data Fig. 1. The GABA-T phylogeny was iteratively refined. The topology of an initial gene tree using GABA-T orthogroups indicated that GABA-T orthologs from *N.* *benthamiana*, *S.* *tuberosum* and *P.* *floridana* had incorrect gene models. To correct this, the genomic regions for these incorrect gene models were retrieved and *S.* *lycopersicum* GABA-T protein sequences were aligned to the genome using miniprot^67^. The resulting exon–intron structures were manually curated and open-reading frames were identified using Geneious Prime 2023.1.2. GABA-T homologs for *P.* *inflata* were identified by BLAST and similarly corrected using the *P.* *inlfata* genome^68^. *S.* *americanum* orthologs were obtained by nucleotide BLAST of *S.* *lycopersicum* GABA-T orthologs against the predicted coding sequences for the SP1102 genome^69^. To better understand the species topology of the GABA-T orthologs, we expanded the phylogeny to include the GABA-T orthologs (OG0005004) from the OneKP dataset^70^ with transcripts below 900 bp excluded. A nonredundant set of coding sequences (including the corrected genome models) were aligned using MAFFT and a phylogenetic tree inferred using FastTree^71^. The tree was anchored using the OneKP outgroup. From this large phylogeny, we identified the Solanales clade and performed a codon alignment. A *Catharanthus roseus* ortholog from the OneKP dataset was included as an outgroup to ensure correct rooting in the presence of multiple lineage-specific duplications. Gaps were removed using the gappyout algorithm of TrimAl followed by maximum-likelihood tree inference using IQ-Tree 2 (ref. ^61^) with ModelFinder^62^, 1,000 ultrafast bootstraps (UFBoot2)^63^ and 1,000 SH-aLRT supports^64^. Corrected gene models used for phylogenetic analysis are provided in Extended Data Fig. 1.

### Selection analysis

The codon alignment used to make the GABA-T maximum-likelihood gene tree was used for diversifying selection analysis using a combination of analyses from the DataMonkey webserver (https://datamonkey.org/)^72^. For assessing branches under selection, we used aBSREL, an adaptive branch-site REL test for episodic diversification^31^. The aBSREL analysis was run with default settings. Test branches were selected to detect the presence or timing of selection in the evolution from the Solanaceae GABA-T ancestor to major clades including the GAME12 subclade (Supplementary Fig. 26b). To identify specific sites under selection across branches of interest, we used BUSTED^73^ and MEME^33^. The BUSTED (version 4.1) analysis included site-to-site synonymous rate variation to reduce false positives^73^ and an evidence ratio threshold of 5. Branch-site tests using empirical Bayes factors were used to identify the codons changing over a priori branches of interest (Supplementary Fig. 26a). Furthermore, sites under episodic selection across a proportion of branches were assessed with MEME. Sites with MEME *P* < 0.05 were then assessed in the BUSTED output to identify sites that were under selection and featured nonsynonymous mutations across branches of interest. According to the BUSTED and MEME output, sites that had evidence of positive or diversifying selection across the branches from GABA-T3 to *S.* *lycopersicum* GAME12 were selected and mutations of *S.* *lycopersicum* GABA-T3 were designed on this basis (Supplementary Table 4).

### Cloning, expression and purification of GABA-T3 mutants

The GABA-T3 mutant coding sequences were purchased as synthetic genes (Twist Bioscience) with included overhangs for InFusion cloning into the pOPINF expression plasmid. The methods used for cloning, expression and purification of GABA-T3 mutant proteins were identical to corresponding methods used for GABA-T3 homologs.

### Modeling of GABA-T homologs and ligand docking

GABA-T homologs and GABA-T3 mutants were modeled as homodimers^74^, using the ColabFold version of AlphaFold^75^ in the AlphaFold multimer V3 mode. All the settings were kept as default. For docking of the GABA–PLP external aldimine complex, the structure of the complex was retrieved from a reported crystal structure (Protein Data Bank (PDB) 4ATQ)^40^. The 26-furostanol aldehyde (**4**) structure was generated in Chemdraw (Perkin Elmer) and converted into a mol2 file. Both substrates were docked into the GABA-T AlphaFold models using the Webina online AutoDock Vina server^41^. The docking results were visualized in UCSF Chimera (version 1.17.1)^76^ and manually assessed for proximity of the GABA–PLP external aldimine complex to the conserved PLP-binding residues. The accuracy of the 26-furostanol aldehyde docking was assessed on the basis of the proximity and correct orientation of the substrate to the catalytic lysine residue and the PMP cofactor (embedded in the structure based on the GABA–PLP external aldimine complex docking). The figures depicting the docking results were prepared in PyMol version 4.50 software (Schrödinger) and UCSF ChimeraX version 1.5 software^77^.

### In vitro GABA-T-catalyzed l-alanine (11) formation assays

In vitro assays on GABA-T homologs and GABA-T3 mutants using pyruvate and GABA as cosubstrates were carried out as follows: reaction mixtures were set up to contain 50 mM Tris buffer pH 9.0, 0.5 mM pyruvate, 5 mM GABA and 0.1 mM PLP. The final protein concentration was 50 nM for GABA-T1, 1 μM for GAME12, GABA-T3, GABA-T4 and GABA-T3 mut3 and 1 μM total protein (because of poor yield) for GABA-T3 mut1 and GABA-T3 mut2. The assays were incubated at 30 °C for either 20 min in the case of GABA-T1 or 30 min in the case of the other tested proteins with 750 r.p.m. shaking. The assay mixtures were then extracted with 1× assay volume of 75% ethanol in H_2_O, vortexed briefly and centrifuged for 20 min at top speed to precipitate the proteins. The resulting mixture was analyzed using UHPLC–ESI-MS as described below.

### UHPLC–MS analysis of in vitro l-alanine (11)-formation

Extracts were analyzed using a Thermo UltiMate 3000 UHPLC system (Thermo Fisher Scientific) coupled with an EVOQ-TQS Elite mass spectrometer (Bruker) and the Bruker Daltonics MSWS version 8.2.1. and Bruker Compass Hystar version 5.1.8.1. software. Separation was performed on a Phenomenex Luna Omega PS C18 column (2.1 × 150 mm, 5 μm, 100 Å) at a column temperature of 30 °C. The first 40 s of each run was redirected to waste. Solvent A was 0.01% formic acid in water; solvent B was 100% methanol. The gradient was as follows: 2% B from 0 to 2 min, to 100% B at 3 min, held at 100% B until 4 min and re-equilibrated to 2% B for 3 min. The flow was 0.25 ml min^−1^ and the injection volume was 2 μl. The samples were ionized using a heated ESI^+^ with spray voltage of 4,000 V, cone temperature of 350 °C, cone gas flow of 20 psi, heated probe temperature of 400 °C, probe gas flow of 45 psi and nebulizer gas flow of 55 psi. Quantification of l-alanine (**11**) was based on the selective reaction monitoring of the l-alanine *m/z* 90.00 precursor ion to *m/z* 44.30 product ion using the collision energy of 9 eV. The scan time was 166.7 ms. Unit resolution was applied to Q1 and standard resolution was applied to Q3. The scan time was 166.7 ms. Acquired data were analyzed using the Bruker MS Data Review version 8.2.1 software.

### *Agrobacterium*-mediated stable transformation of *S.nigrum*

*S.* *nigrum* (wild type, SN30 bulk) seeds were obtained from seed stocks maintained by the greenhouse team at the Max Planck Institute for Chemical Ecology. Seeds were sterilized using a solution of 0.1 g of sodium dichloroisocyanurate dihydrate and 50 μl of 0.5% Tween 20 in 5 ml of deionized sterile water. Seeds were incubated in the sterilizing solution for 5 min, washed three times with deionized sterile water and then incubated overnight in 5 ml of sterile 1 M KNO_3_ solution at 4 °C in darkness. Seeds were germinated on Gamborg B5 medium for 7 days under the following day–night cycle: 16 h of light, 26 °C; 8 h of darkness, 24 °C. Plasmid-harboring *A.* *tumefaciens* (strain GV3101) was used to inoculate 8-ml LB media cultures supplemented with 200 μg ml^−1^ spectinomycin, 100 μg ml^−1^ rifampicin and 50 μg ml^−1^ gentamycin. The cultures were grown until the culture OD_600_ reached 0.4–0.8. The cells were pelleted by centrifugation at 4,000*g* at room temperature for 5 min. The pellet was resuspended in 7 ml of *Agrobacterium* washing medium (basal medium: 4.41 g L^−1^ Murashige and Skoog containing vitamins and 30 g L^−1^ sucrose, supplemented with 0.02 mg L^−1^ indole-3-acetic acid (IAA) and 1 mg L^−1^ 6-benzylaminopurine (BAP), pH 5.80). The seedling hypocotyls were cut into 3-mm-long pieces using a sterile scalpel dipped in the *A.* *tumefaciens* suspension. The explants were transferred onto callus induction medium (basal medium supplemented with 3 g L^−1^ Phytagel, 0.02 mg L^−1^ IAA and 1 mg L^−1^ BAP, pH 5.80) and incubated at 26 °C in darkness for 3 days. The explants were then subcultured onto fresh callus induction medium plates containing antibiotics (25 mg L^−1^ kanamycin and 125 mg L^−1^ timentin) and incubated at 30 °C for 16 h and 27 °C for 8 h for a total of 14 days. The resulting callus was transferred onto shoot induction media (basal medium supplemented with 0.5 mg L^−1^ BAP, 25 mg L^−1^ kanamycin and 125 mg L^−1^ timentin) and incubated at 30 °C for 16 h and 27 °C for 8 h for a total of 7 days. For shoot maturation, the calli with primordia shoots were subcultured on maturation medium (basal medium supplemented with 25 mg L^−1^ kanamycin and 125 mg L^−1^ timentin) at 28 °C for 16 h and 24 °C for 8 h for 7 days. The resulting plantlets were separated and transferred onto rooting medium (292 mg L^−1^ of Peter’s hydrosol salts, 2 ml of 500× Murashige and Skoog vitamin solution and 6 g L^−1^ plant agar supplemented with 125 mg L^−1^ timentin, pH 6.0). The plantlets were placed in a culture room with a constant 22 °C temperature and a photopetiod of 16 h light and 8 h darkness and subcultured onto fresh rooting medium plates every 14 days. After rooting, the plants were transferred to soil in Magenta boxes (100 mm × 77 mm × 77 mm) and finally planted in 2-L pots in the greenhouse for breeding under a long-day photoperiod (16 h light, 8 h dark) at 25 °C and 65% relative humidity.

### qPCR analysis

Total RNA was isolated from *S.* *nigrum* wild-type plants (young leaves and green fruits) and transgenic lines (young leaves) with the RNeasy mini kit (Qiagen) according to the manufacturer’s instructions. Unless stated otherwise, at least three biological replicates from each genotype were used for gene expression analysis (*n* ≥ 3). Three biological replicates (for each genotype) denote three tissue samples obtained from three independently grown wild-type or corresponding transgenic line plants. DNase I-treated (Sigma-Aldrich) RNA was reverse-transcribed using a high-capacity cDNA reverse transcription kit (Applied Biosystems). Gene-specific primers were designed with the Primer BLAST software (National Center for Biotechnology Information (NCBI)). The sequences of all primers used in qPCR analysis can be found in Supplementary Table 7. The *EF1α* gene was used as a reference gene in the analysis^78^.

### UHPLC–MS analysis of *S.nigrum* transgenic line leaves

The leaf tissue of the *S.* *nigrum* transformant lines was collected, snap-frozen in liquid N_2_ and pulverized. Metabolites were extracted using 80% methanol with 0.1% formic acid. For 100 mg of weighed-out frozen tissue, 300 μl of the extraction solvent was added. The resulting mixtures were vortexed briefly and sonicated in a sonic bath for 15 min. The extracts were centrifuged at top speed for 15 min and passed through a 0.22-μm PTFE filter (Thermo Fisher Scientific). Extracts were analyzed using a Waters UHPLC system (Waters Acquity) coupled to a SYANPT-G2QTOF MS instrument (Waters Acquity). Separation was performed on a Waters Acquity Premier BEH C18 Vanguard FIT column (2.1 × 100 mm, 1.7 μm, 130 Å). Solvent A was 0.1% formic acid in 5% acetonitrile in water; solvent B was 0.1% formic acid in acetonitrile. The gradient was as follows: 0% at 0 min to 28% B at 22 min and 28% to 100% B at 36 min. The column was flushed with 100% B for 2 min, re-equilibrated to 0% B within 0.5 min and conditioned at 0% B for 1.5 min. The samples were ionized using a pneumatic-assisted ESI^+^ with a capillary voltage of 3,400 V, cone voltage of 24 V, source temperature of 125 °C, desolvation temperature of 275 °C and desolvation gas flow of 650 L h^−1^. The spectra were acquired within the 50–1,600 *m/z* scan range. Data were acquired using the MS^E^ mode with energy ramp. The collision energy was set to 4 eV at the low-energy acquisition function and a 10–30 eV ramp for the high-energy function. Sodium formate solution and Leu encephalin were used at the lock mass. Acquired data were analyzed using the MassLynx version 4.2 software. The putative structural assignment of SGAs and steroidal saponins produced by the transgenic *S.* *nigrum* plants was based on the identification of characteristic, previously reported fragment ions^4,47^.

### Reporting summary

Further information on research design is available in the Nature Portfolio Reporting Summary linked to this article.

## Online content

Any methods, additional references, Nature Portfolio reporting summaries, source data, extended data, supplementary information, acknowledgements, peer review information; details of author contributions and competing interests; and statements of data and code availability are available at 10.1038/s41589-024-01735-w.

## Supplementary information

Supplementary Information Supplementary Figs. 1–36 and Tables 1–7.

Reporting Summary

Supplementary Data 1 Sequences of constructs used in the study.

Supplementary Data 2 Source data for Supplementary Figs. 10, 31, 32b and 35.

## Source data

Source Data Fig. 5 Peak areas for uttroside B and product E.

Source Data Extended Data Fig. 1 Gene sequences used for construction of the phylogenetic tree and sequences of corrected gene models.

## Data Availability

The sequences of the gene constructs used in the study are listed in Supplementary Data 1. The sequences used in comparative genomics analyses, the corrected gene models and the sequences used in phylogeny analyses are listed in Extended Data Fig. 1. *S.* *nigrum* young leaves and green fruit (berries) transcriptome raw sequence reads were deposited to the NCBI Sequence Read Archive under accession numbers PRJNA1134384 and PRJNA1133681, respectively. The accession numbers of the genomes used for the phylogenomics and synteny analysis of GABA-T homologs are provided in Supplementary Table 6. The crystal structure used in the modeling of GABA-T homologs was retrieved from PDB 4ATQ. Data are available from the corresponding authors upon request. Source data are provided with this paper.
