# Supplementary material for: Incorporation of nitrogen in antinutritional Solanum alkaloid biosynthesis
Source: Nat Chem Biol. 2024 Sep 13;21(1):131–42. doi: 10.1038/s41589-024-01735-w (PMC11666457; doi:10.1038/s41589-024-01735-w)
Supplement: Supplementary file 2 — Reporting Summary [file 41589_2024_1735_MOESM2_ESM.pdf]

nature portfolio

Corresponding author(s): Sarah E. O'Connor, Asaph Aharoni, Prashant D. Sonawane

Last updated by author(s): 25/07/24

Reporting Summary

Nature Portfolio wishes to improve the reproducibility of the work that we publish. This form provides structure for consistency and transparency in reporting. For further information on Nature Portfolio policies, see our Editorial Policies and the Editorial Policy Checklist.

Statistics

For all statistical analyses, confirm that the following items are present in the figure legend, table legend, main text, or Methods section.

n/a	Confirmed	
☐	☒	The exact sample size (*n*) for each experimental group/condition, given as a discrete number and unit of measurement
☐	☒	A statement on whether measurements were taken from distinct samples or whether the same sample was measured repeatedly
☐	☒	The statistical test(s) used AND whether they are one- or two-sided<br>*Only common tests should be described solely by name; describe more complex techniques in the Methods section.*
☒	☐	A description of all covariates tested
☐	☒	A description of any assumptions or corrections, such as tests of normality and adjustment for multiple comparisons
☐	☒	A full description of the statistical parameters including central tendency (e.g. means) or other basic estimates (e.g. regression coefficient) AND variation (e.g. standard deviation) or associated estimates of uncertainty (e.g. confidence intervals)
☐	☒	For null hypothesis testing, the test statistic (e.g. *F*, *t*, *r*) with confidence intervals, effect sizes, degrees of freedom and *P* value noted<br>*Give P values as exact values whenever suitable.*
☒	☐	For Bayesian analysis, information on the choice of priors and Markov chain Monte Carlo settings
☒	☐	For hierarchical and complex designs, identification of the appropriate level for tests and full reporting of outcomes
☒	☐	Estimates of effect sizes (e.g. Cohen's *d*, Pearson's *r*), indicating how they were calculated

Our web collection on statistics for biologists contains articles on many of the points above.

Software and code

Policy information about availability of computer code

Data collection	All data presented in the manuscript was acquired using pre-existing, routinely used software. The software used is mentioned in the respective parts of the manuscript. Liquid chromatography-mass spectrometry data was collected using Bruker Compass qtofControl 6.3/ Bruker Compass Hystar v 6.0.30.0 software; Bruker Daltonics MSWS v 8.2.1/ Bruker Compass Hystar v 5.1.8.1 software or MassLynx v 4.2 software. NMR data was collected using Bruker TopSpin v 3.6.1 software. Confocal microscopy images were collected using ZEN black v 2.1 software.
Data analysis	Liquid chromatography-mass spectrometry data was analyzed using Bruker Compass DataAnalysis v 6.0.313.6.0 software, Bruker Daltonics MS Data Review v 8.2.1 or MassLynx v 4.2 software. Confocal microscopy images were analyzed and adjusted using ImageJ v 1.54f. NMR data was analyzed using Bruker TopSpin v 3.6.1 software. Comparative genomic analyses of GABA-T orthologoues and phylogeny inference were carried out using: AGAT v 1.2.0, Orthofinder 2, IQ-Tree 2 v2.2.2.6, MCScanX and Geneious Prime v 2023.1.2. Diversifying selection was analyzed using UFBoot2. GABA-T protein structure modelling was carried out using ColabFold version of AlphaFold multimer V3. Substrate docking was carried out using Webina online AutoDock Vina server 1.0.5. The docking results were visualized in UCSF Chimera v 1.17.1. and PyMol v 4.50. Heatmaps were generated in Morpehus (https://software.broadinstitute.org/morpheus/). Graphs were generated in GraphPad Prism v 9.5.1 and SigmaPlot v 14.03.192. Figures were prepared in Adobe Illustrator CS5 V15.0.

For manuscripts utilizing custom algorithms or software that are central to the research but not yet described in published literature, software must be made available to editors and reviewers. We strongly encourage code deposition in a community repository (e.g. GitHub). See the Nature Portfolio guidelines for submitting code & software for further information.

Data

Policy information about availability of data

All manuscripts must include a data availability statement. This statement should provide the following information, where applicable:

- Accession codes, unique identifiers, or web links for publicly available datasets
- A description of any restrictions on data availability
- For clinical datasets or third party data, please ensure that the statement adheres to our policy

The sequences of gene constructs used in the study are listed in Supplementary Data 1. The sequences used in comparative genomics analyses, the corrected gene models and sequences used in phylogeny analyses are listed in Extended Data Source Data 1. S. nigrum young leaves and green fruit (berries) transcriptome raw sequence reads have been deposited in the NCBI Sequence Read Archive under the Bio project IDs PRJNA1134384 (https://www.ncbi.nlm.nih.gov/sra/?term=PRJNA1134384) and PRJNA1133681 (https://www.ncbi.nlm.nih.gov/sra/?term=PRJNA1133681), respectively. The accession numbers of the genomes used for the phylogenomics and synteny analysis of GABA-T homologues are provided in Supplementary Table 6. The crystal structure used in the modelling of GABA-T homologues was retrieved from PDB with identifier 4ATQ (https://www.rcsb.org/structure/4ATQ). Source data are provided with this paper. Data is available from the corresponding authors upon request.

Research involving human participants, their data, or biological material

Policy information about studies with human participants or human data. See also policy information about sex, gender (identity/presentation), and sexual orientation and race, ethnicity and racism.

Reporting on sex and gender	Not applicable
Reporting on race, ethnicity, or other socially relevant groupings	Not applicable
Population characteristics	Not applicable
Recruitment	Not applicable
Ethics oversight	Not applicable

Note that full information on the approval of the study protocol must also be provided in the manuscript.

Field-specific reporting

Please select the one below that is the best fit for your research. If you are not sure, read the appropriate sections before making your selection.

☒ Life sciences ☐ Behavioural & social sciences ☐ Ecological, evolutionary & environmental sciences

For a reference copy of the document with all sections, see nature.com/documents/nr-reporting-summary-flat.pdf

Life sciences study design

All studies must disclose on these points even when the disclosure is negative.

Sample size	Prior determination of sample size is not applicable to our study. N. benthamiana pathway reconstitution-based and enzyme assay-based were conducted using at least three independent biological replicates on at least two different days to ensure reproducibility of the data. Microscopy experiments were conducted on at least two biological replicates and on at least two different days, to ensure reproducibility.
Data exclusions	No data was excluded.
Replication	Details of biological/technical replicates are provided in respective main figure legends, Supplementary Figure legends and methods sub-sections. The data presented for transgenic S. nigrum lines was obtained from three biological replicates coming from three independent transgenic lines (T1 generation). For heterologous expression in N. benthamiana, data presented was obtained from three biological replicates, collected from three independently grown N. benthamiana plants. In vitro enzyme assays were successfully performed with at least three replicates. Enzyme assay experiments were repeated at least twice. 26-furostanol aldehyde trapping experiment was successfully performed twice.
Randomization	The order of the samples analyzed using UHPLC-MS was randomized prior to the runs. In the enzyme assays and N. benthamiana pathway reconsitution-based experiments, we sampled at least three different biological replicates (three different assay set-ups and three different plants) in two independent experiments conducted on different days. Other types of randomization are not applicable to our study.
Blinding	Blinding was not relevant for our study; characterization of enzymes conducted in this publication requires insight into the experimental conditions and characteristics of the samples.

Behavioural & social sciences study design

All studies must disclose on these points even when the disclosure is negative.

Study description	*Briefly describe the study type including whether data are quantitative, qualitative, or mixed-methods (e.g. qualitative cross-sectional, quantitative experimental, mixed-methods case study).*
Research sample	*State the research sample (e.g. Harvard university undergraduates, villagers in rural India) and provide relevant demographic information (e.g. age, sex) and indicate whether the sample is representative. Provide a rationale for the study sample chosen. For studies involving existing datasets, please describe the dataset and source.*
Sampling strategy	*Describe the sampling procedure (e.g. random, snowball, stratified, convenience). Describe the statistical methods that were used to predetermine sample size OR if no sample-size calculation was performed, describe how sample sizes were chosen and provide a rationale for why these sample sizes are sufficient. For qualitative data, please indicate whether data saturation was considered, and what criteria were used to decide that no further sampling was needed.*
Data collection	*Provide details about the data collection procedure, including the instruments or devices used to record the data (e.g. pen and paper, computer, eye tracker, video or audio equipment) whether anyone was present besides the participant(s) and the researcher, and whether the researcher was blind to experimental condition and/or the study hypothesis during data collection.*
Timing	*Indicate the start and stop dates of data collection. If there is a gap between collection periods, state the dates for each sample cohort.*
Data exclusions	*If no data were excluded from the analyses, state so OR if data were excluded, provide the exact number of exclusions and the rationale behind them, indicating whether exclusion criteria were pre-established.*
Non-participation	*State how many participants dropped out/declined participation and the reason(s) given OR provide response rate OR state that no participants dropped out/declined participation.*
Randomization	*If participants were not allocated into experimental groups, state so OR describe how participants were allocated to groups, and if allocation was not random, describe how covariates were controlled.*

Ecological, evolutionary & environmental sciences study design

All studies must disclose on these points even when the disclosure is negative.

Study description	*Briefly describe the study. For quantitative data include treatment factors and interactions, design structure (e.g. factorial, nested, hierarchical), nature and number of experimental units and replicates.*
Research sample	*Describe the research sample (e.g. a group of tagged Passer domesticus, all Stenocereus thurberi within Organ Pipe Cactus National Monument), and provide a rationale for the sample choice. When relevant, describe the organism taxa, source, sex, age range and any manipulations. State what population the sample is meant to represent when applicable. For studies involving existing datasets, describe the data and its source.*
Sampling strategy	*Note the sampling procedure. Describe the statistical methods that were used to predetermine sample size OR if no sample-size calculation was performed, describe how sample sizes were chosen and provide a rationale for why these sample sizes are sufficient.*
Data collection	*Describe the data collection procedure, including who recorded the data and how.*
Timing and spatial scale	*Indicate the start and stop dates of data collection, noting the frequency and periodicity of sampling and providing a rationale for these choices. If there is a gap between collection periods, state the dates for each sample cohort. Specify the spatial scale from which the data are taken*
Data exclusions	*If no data were excluded from the analyses, state so OR if data were excluded, describe the exclusions and the rationale behind them, indicating whether exclusion criteria were pre-established.*
Reproducibility	*Describe the measures taken to verify the reproducibility of experimental findings. For each experiment, note whether any attempts to repeat the experiment failed OR state that all attempts to repeat the experiment were successful.*
Randomization	*Describe how samples/organisms/participants were allocated into groups. If allocation was not random, describe how covariates were controlled. If this is not relevant to your study, explain why.*
Blinding	*Describe the extent of blinding used during data acquisition and analysis. If blinding was not possible, describe why OR explain why blinding was not relevant to your study.*

Did the study involve field work? ☐ Yes ☐ No

Field work, collection and transport

Field conditions	*Describe the study conditions for field work, providing relevant parameters (e.g. temperature, rainfall).*
Location	*State the location of the sampling or experiment, providing relevant parameters (e.g. latitude and longitude, elevation, water depth).*
Access & import/export	*Describe the efforts you have made to access habitats and to collect and import/export your samples in a responsible manner and in compliance with local, national and international laws, noting any permits that were obtained (give the name of the issuing authority, the date of issue, and any identifying information).*
Disturbance	*Describe any disturbance caused by the study and how it was minimized.*

Reporting for specific materials, systems and methods

We require information from authors about some types of materials, experimental systems and methods used in many studies. Here, indicate whether each material, system or method listed is relevant to your study. If you are not sure if a list item applies to your research, read the appropriate section before selecting a response.

Materials & experimental systems

n/a	Involved in the study
☒	☐ Antibodies
☒	☐ Eukaryotic cell lines
☒	☐ Palaeontology and archaeology
☒	☐ Animals and other organisms
☒	☐ Clinical data
☒	☐ Dual use research of concern
☐	☒ Plants

Methods

n/a	Involved in the study
☒	☐ ChIP-seq
☒	☐ Flow cytometry
☒	☐ MRI-based neuroimaging

Antibodies

Antibodies used	not applicable
Validation	not applicable

Eukaryotic cell lines

Policy information about cell lines and Sex and Gender in Research

Cell line source(s)	not applicable
Authentication	not applicable
Mycoplasma contamination	not applicable
Commonly misidentified lines (See ICLAC register)	not applicable

Palaeontology and Archaeology

Specimen provenance	not applicable
Specimen deposition	not applicable
Dating methods	not applicable

☐ Tick this box to confirm that the raw and calibrated dates are available in the paper or in Supplementary Information.

Ethics oversight	not applicable

Note that full information on the approval of the study protocol must also be provided in the manuscript.

Animals and other research organisms

Policy information about studies involving animals; ARRIVE guidelines recommended for reporting animal research, and Sex and Gender in Research

Laboratory animals	not applicable
Wild animals	not applicable
Reporting on sex	not applicable
Field-collected samples	not applicable
Ethics oversight	not applicable

Note that full information on the approval of the study protocol must also be provided in the manuscript.

Clinical data

Policy information about clinical studies

All manuscripts should comply with the ICMJE guidelines for publication of clinical research and a completed CONSORT checklist must be included with all submissions.

Clinical trial registration	not applicable
Study protocol	not applicable
Data collection	not applicable
Outcomes	not applicable

Dual use research of concern

Policy information about dual use research of concern

Hazards

Could the accidental, deliberate or reckless misuse of agents or technologies generated in the work, or the application of information presented in the manuscript, pose a threat to:

No	Yes	
☒	☐	Public health
☒	☐	National security
☒	☐	Crops and/or livestock
☒	☐	Ecosystems
☒	☐	Any other significant area

Experiments of concern

Does the work involve any of these experiments of concern:

No	Yes	
☒	☐	Demonstrate how to render a vaccine ineffective
☒	☐	Confer resistance to therapeutically useful antibiotics or antiviral agents
☒	☐	Enhance the virulence of a pathogen or render a nonpathogen virulent
☒	☐	Increase transmissibility of a pathogen
☒	☐	Alter the host range of a pathogen
☒	☐	Enable evasion of diagnostic/detection modalities
☒	☐	Enable the weaponization of a biological agent or toxin
☒	☐	Any other potentially harmful combination of experiments and agents

Plants

Seed stocks	Solanum nigrum (wild type, SN30 bulk) seeds were obtained from seed stocks maintained by the greenhouse team at Max Planck Institute for Chemical Ecology, Jena. Nicotiana benthamiana seeds were obtained from seed stocks maintained by the greenhouse team at Max Planck Institute for Chemical Ecology, Jena.
Novel plant genotypes	The stable transformation of S. nigrum plants was carried out as described in the 'Agrobacterium- mediated stable transformations of S. nigrum' materials and methods paragraph. Hetereologous overexpression in N. benthamiana was carried out as described in the 'Testing of GABA-T homologues using the pathway reconstitution approach in N. benthamiana' paragraph.
Authentication	Heterologous overexpression in N. benthamiana metabolite profiling was carried out as described in 'UHPLC-MS analysis of N. benthamiana extracts ' methods paragraph. The S. nigrum stable transformation lines metabolite profiling was carried out as described in the 'UHPLC-MS analysis of leaves from S. nigrum stable transformant lines' materials and methods paragraph.

ChIP-seq

Data deposition

- [ ] Confirm that both raw and final processed data have been deposited in a public database such as GEO.
- [ ] Confirm that you have deposited or provided access to graph files (e.g. BED files) for the called peaks.

Data access links<br>*May remain private before publication.*	not applicable
Files in database submission	not applicable
Genome browser session<br>(e.g. UCSC)	not applicable

Methodology

Replicates	not applicable
Sequencing depth	not applicable
Antibodies	not applicable
Peak calling parameters	not applicable
Data quality	not applicable
Software	not applicable

Flow Cytometry

Plots

Confirm that:

- [ ] The axis labels state the marker and fluorochrome used (e.g. CD4-FITC).
- [ ] The axis scales are clearly visible. Include numbers along axes only for bottom left plot of group (a 'group' is an analysis of identical markers).
- [ ] All plots are contour plots with outliers or pseudocolor plots.
- [ ] A numerical value for number of cells or percentage (with statistics) is provided.

Methodology

Sample preparation	not applicable
Instrument	not applicable
Software	not applicable
Cell population abundance	not applicable

Gating strategy | not applicable

☐ Tick this box to confirm that a figure exemplifying the gating strategy is provided in the Supplementary Information.

Magnetic resonance imaging

Experimental design

Design type | not applicable

Design specifications | not applicable

Behavioral performance measures | not applicable

Acquisition

Imaging type(s) | not applicable

Field strength | not applicable

Sequence & imaging parameters | not applicable

Area of acquisition | not applicable

Diffusion MRI ☐ Used ☐ Not used

Preprocessing

Preprocessing software | not applicable

Normalization | not applicable

Normalization template | not applicable

Noise and artifact removal | not applicable

Volume censoring | not applicable

Statistical modeling & inference

Model type and settings | not applicable

Effect(s) tested | not applicable

Specify type of analysis: ☐ Whole brain ☐ ROI-based ☐ Both

Statistic type for inference | not applicable

(See Eklund et al. 2016)

Correction | not applicable

Models & analysis

n/a	Involved in the study
☐	☐ Functional and/or effective connectivity
☐	☐ Graph analysis
☐	☐ Multivariate modeling or predictive analysis

Functional and/or effective connectivity | *Report the measures of dependence used and the model details (e.g. Pearson correlation, partial correlation, mutual information).*

Graph analysis | *Report the dependent variable and connectivity measure, specifying weighted graph or binarized graph, subject- or group-level, and the global and/or node summaries used (e.g. clustering coefficient, efficiency, etc.).*

Multivariate modeling and predictive analysis | *Specify independent variables, features extraction and dimension reduction, model, training and evaluation*

Multivariate modeling and predictive analysis

metrics.
