# Supplementary material for: Incorporation of nitrogen in antinutritional Solanum alkaloid biosynthesis
Source: Nat Chem Biol. 2024 Sep 13;21(1):131–42. doi: 10.1038/s41589-024-01735-w (PMC11666457; doi:10.1038/s41589-024-01735-w)
Supplement: Supplementary file 1 — Supplementary Figs. 1–36 and Tables 1–7. [file 41589_2024_1735_MOESM1_ESM.pdf]

nature chemical biology

Supplementary information https://doi.org/10.1038/s41589-024-01735-w

Incorporation of nitrogen in antinutritional *Solanum* alkaloid biosynthesis

In the format provided by the authors and unedited

Table of Contents

Supplementary Figure 1. Amino acid sequence alignment of the four GABA-T homologues found in tomato (*S. lycopersicum*)........ 3
Supplementary Figure 2. Sub-cellular localization of the tomato GABA-T homologues........ 4
Supplementary Figure 3. The MS^2 spectra of assay products observed in biosynthetic pathway reconstitution in *N. benthamiana*........ 5
Supplementary Figure 4. The expression of GAME12 and GABA-T4 across two different cultivated tomato varieties........ 6
Supplementary Figure 5. Sub-cellular localization of the *S. lycopersicum* GABA-T homologue truncated variants........ 6
Supplementary Figure 6. The trapping of 26-furostanol aldehyde *via* reductive amination........ 7
Supplementary Figure 7. The MS spectra of the products observed in coupled *in vitro* assays with protodioscin and the corresponding standards........ 8
Supplementary Figure 8. The disappearance of Product A1 and Product A2, putatively assigned as 25(*R*) and 25(*S*) epimers of 26-aminofurostanol, the uncyclized intermediate of solasodine and dehydrotomatidine........ 9
Supplementary Figure 9. The proposed mechanism of spriosolane SGA aglycone F-ring formation, based on the results of the in vitro assays, trapping of the 26-furostanol aldehyde and previous labelling studies........ 10
Supplementary Figure 10. Comparison of the peak area of the solasodine product of *in vitro* GAME12 assays using either GABA or L-alanine as an amino donor........ 11
Supplementary Figure 11. The UHPLC-MS data of uttroside B isolated from *S. nigrum* leaves........ 11
Supplementary Figure 12. Chemical structure of uttroside B........ 12
Supplementary Figure 13. Uttroside B, ^{1}H NMR, full range, in pyridine-d_5........ 13
Supplementary Figure 14. Uttroside B, ^{1}H NMR, sugar range, in pyridine-d_5........ 13
Supplementary Figure 15. Uttroside B, ^{1}H NMR, aglycone range, in pyridine-d_5........ 14
Supplementary Figure 16. Uttroside B, DEPTQ in pyridine-d_5........ 15
Supplementary Figure 17. Uttroside B, phase sensitive HSQC, full range, in pyridine-d_5........ 15
Supplementary Figure 18. Uttroside B, phase sensitive HSQC, sugar range, in pyridine-d_5........ 16
Supplementary Figure 19. Uttroside B, phase sensitive HSQC, aglycone range, in pyridine-d_5........ 16
Supplementary Figure 20. Uttroside B, HMBC, full range, in pyridine-d_5........ 17
Supplementary Figure 21. Uttroside B, gradient-selected DQFCOSY, full range, in pyridine-d_5........ 18
Supplementary Figure 22. Uttroside B, HSQC-TOCSY, full range, in pyridine-d_5........ 18
Supplementary Figure 23. Uttroside B, ROESY, full range, in pyridine-d_5........ 19

Supplementary Figure 24. The MS^2 spectra of the products obtained by *in vitro* coupled assays with uttroside B and the corresponding standards. ... 20
Supplementary Figure 25. Extended *GABA-T* phylogeny. ... 21
Supplementary Figure 26. Chromosome-level microsynteny analysis of GABA-T1, GABA-T3 and GABA-T4 and macrosynteny analysis of GABA-T1 GABA-T3 syntenic blocks. ... 23
Supplementary Figure 27. Site-specific diversifying selection tests. ... 24
Supplementary Figure 28. Complete branch-site Empirical Bayes Factors for selected branches ... 25
Supplementary Figure 29. Alignment of the amino acid sequences of tomato GAME12, GABA-T3 and the GABA-T3 mutants. ... 26
Supplementary Figure 30. The architecture of the active sites of GABA-T3 and the GABA-T3 mutants and the results of mutant protein expression. ... 28
Supplementary Figure 31. Percentage activity of the GABA-Ts on the canonical substrate pyruvate, relative to the mean activity of GABA-T1 ... 29
Supplementary Figure 32. Analysis of the expression of GAME genes in wildtype *S. nigrum* tissues. ... 30
Supplementary Figure 33. UHPLC-MS data for the products of enzymatic hydrolysis of the extracts from the stable *S. nigrum* transgenic lines expressing tomato *GAME12* ... 31
Supplementary Figure 34. UHPLC-MS data for the stable *S. nigrum* transgenic lines ... 32
Supplementary Figure 35. Relative expression of *GAME12* and mitochondrial-*GAME12* version in transgenic *S. nigrum* lines as determined by qRT-PCR. ... 33
Supplementary Figure 36. The uncropped image of the SDS-PAGE analysis of the purified GABA-Ts and GABA-T3 mutants presented in Supplementary Figure 30. ... 33
Supplementary Table 1. Amino acid sequence identity comparison between the four GABA-T homologues identified in tomato (*S. lycopersicum*). ... 34
Supplementary Table 2. ^{1}H- and ^{13}C-NMR data of uttroside B in pyridine-d_5. ... 35
Supplementary Table 3. aBRSEL diversifying selection test. ... 36
Supplementary Table 4. Site-specific diversifying selection tests. ... 37
Supplementary Table 5. The putative fragmentation patterns of soladulcidine-type SGAs produced *de novo* in the leaves of transgenic *S. nigrum* plants. ... 38
Supplementary Table 6. The accession numbers of the genomes used for the phylogenomics and synteny analysis of GABA-T homologues. ... 39
Supplementary Table 7. Sequences of primers used in this study. ... 40

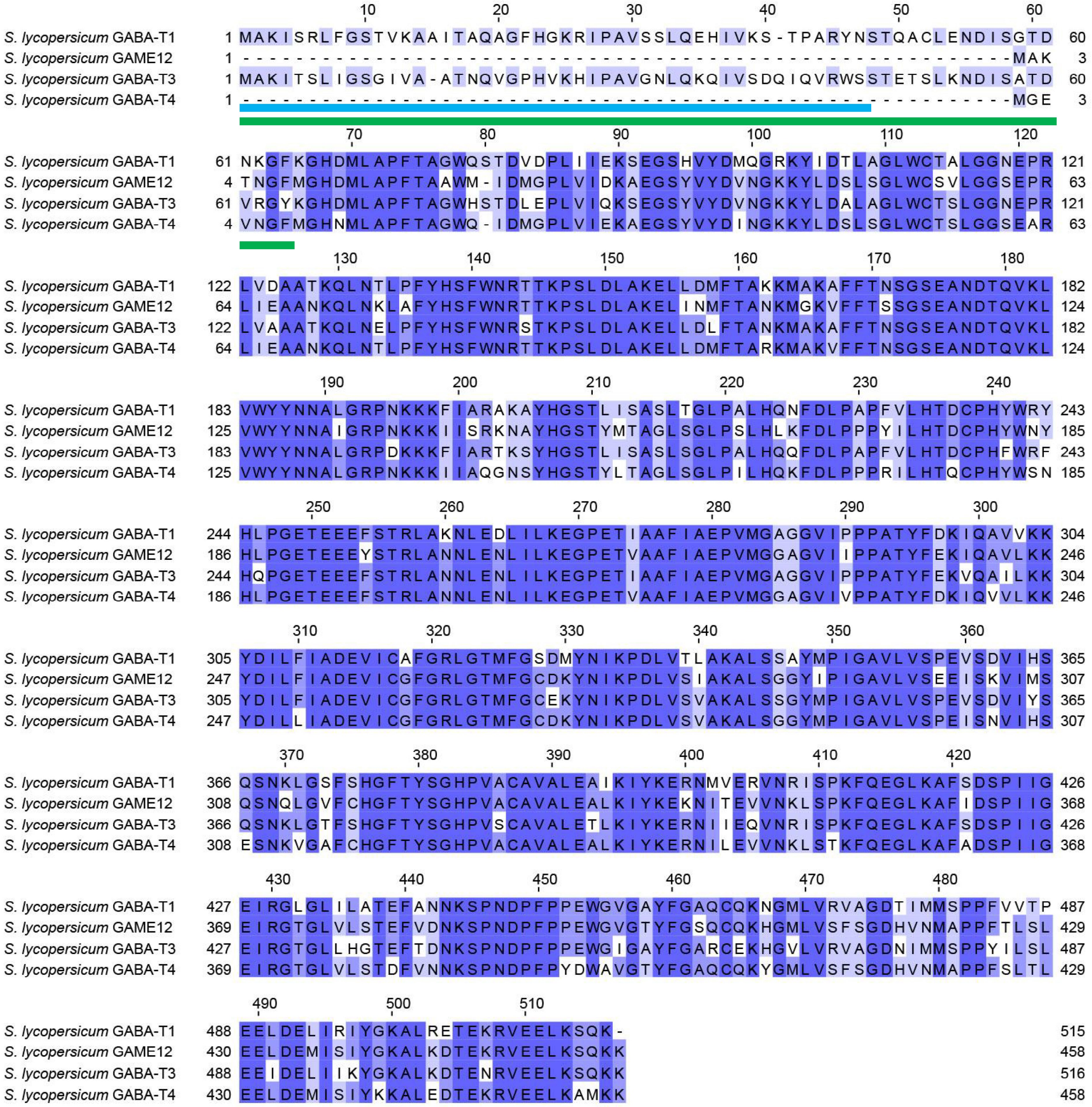

Supplementary Figure 1. Amino acid sequence alignment of the four GABA-T homologues found in tomato (*S. lycopersicum*). The predicted N-terminal signal peptide sequence residues, are highlighted in blue for the mitochondrial GABA-T1 and green for the plastidic GABA-T3[1]. Sequences were aligned using the Clustal Omega algorithm[2]. The alignment was visualized in Jalview[3].

a

b

c

d

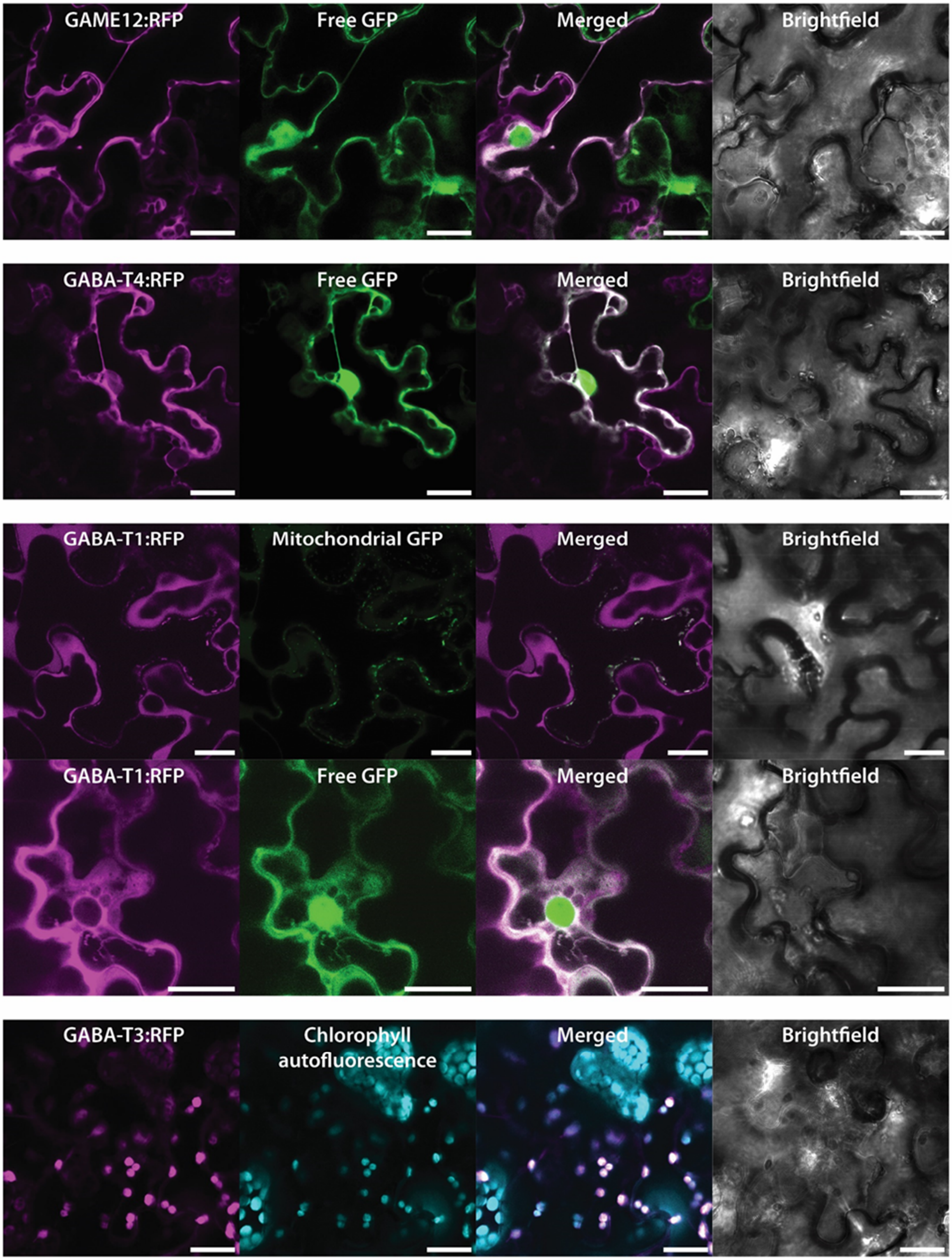

GAME12:RFP
Free GFP
Merged
Brightfield
GABA-T4:RFP
Free GFP
Merged
Brightfield
GABA-T1:RFP
Mitochondrial GFP
Merged
Brightfield
GABA-T1:RFP
Free GFP
Merged
Brightfield
GABA-T3:RFP
Chlorophyll autofluorescence
Merged
Brightfield

Supplementary Figure 2. Sub-cellular localization of the tomato GABA-T homologues. The scale bar represents 10 μm. The results for each GABA-T homologue are representative of at least two independent experiments. **(a-d)** Sub-cellular localization of GAME12/GABA-T2 **(a)**, GABA-T4 **(b)**, GABA-T1 **(c)** and GABA-T3 **(d).** For more experimental details, please refer Materials and Methods section.

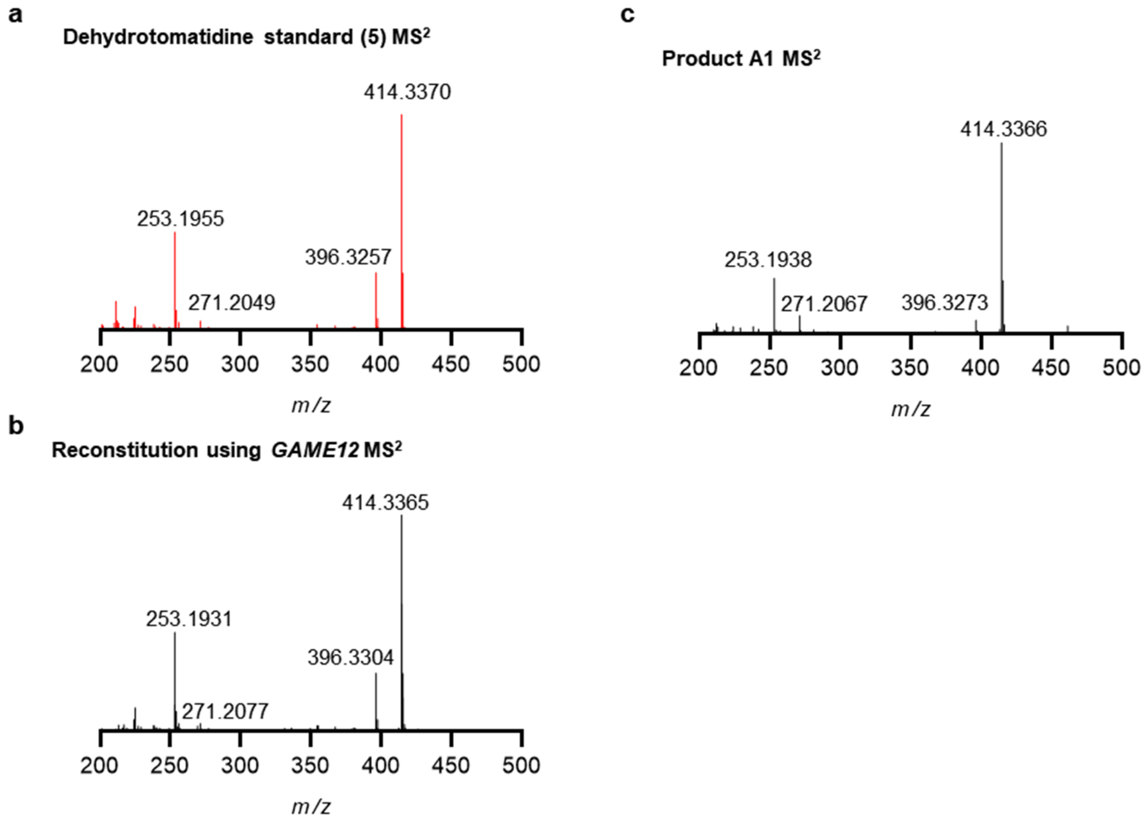

Supplementary Figure 3. The MS^2 spectra of assay products observed in biosynthetic pathway reconstitution in *N. benthamiana*. a. MS^2 spectrum of the dehydrotomatidine (**5**) standard. **b.** MS^2 spectrum of the reconstitution-based GAME12/GABA-T2 assay product peak. **c.** MS^2 spectrum of Product A1.

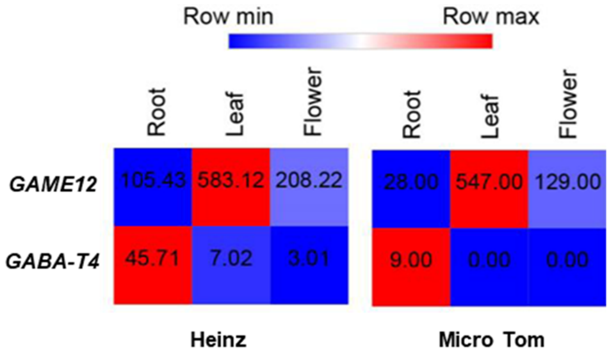

Supplementary Figure 4. The expression of *GAME12* and *GABA-T4* across two different cultivated tomato varieties. The expression values were sourced from the Tomato Functional Genomics Database[4]. Normalized reads per kilobase of transcript per million reads mapped (RPKM) were used for visualization of expression in the Heinz variety. Normalized fragments per kilobase of transcript per million mapped reads (FPKM) values were used for visualization of expression in the Micro Tom variety. The heatmaps were generated in Morpheus (https://software.broadinstitute.org/morpheus).

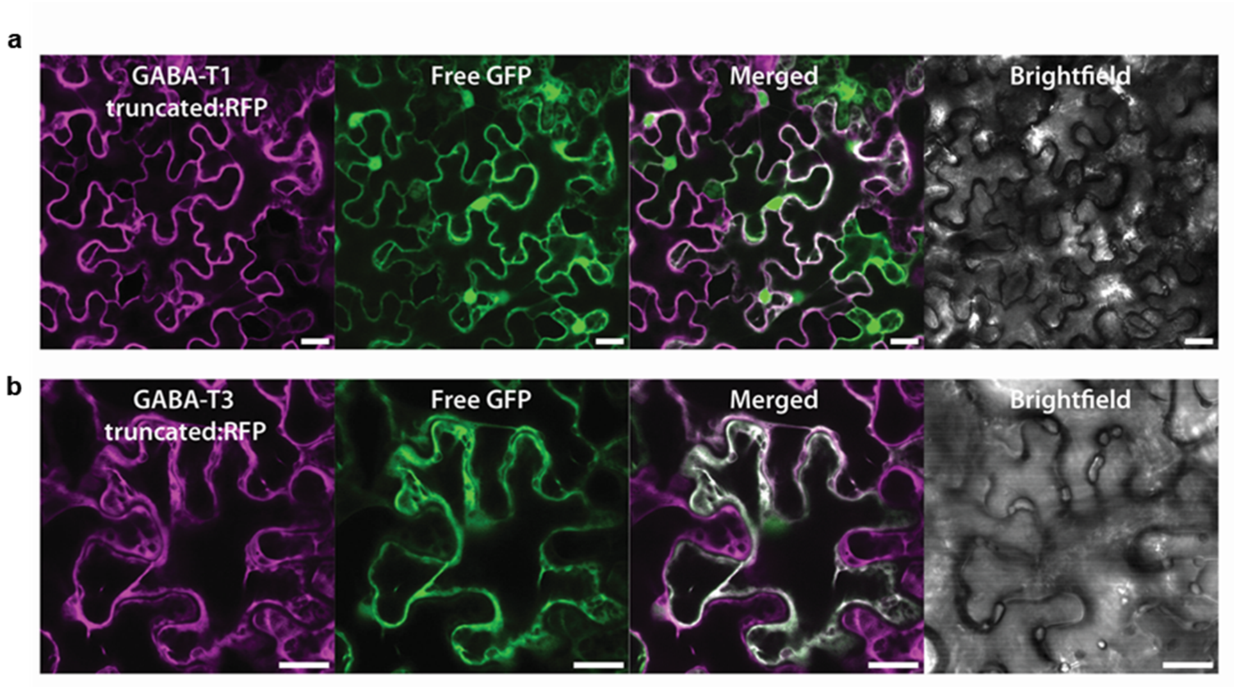

Supplementary Figure 5. Sub-cellular localization of the *S. lycopersicum* GABA-T homologue truncated variants. The scale bar represents 10 μm. The GABA-T1 truncated and GABA-T3 truncated constructs used for the sub-cellular localization analysis were designed based on the previously verified signal peptide sequences reported by Clark et al[1]. See **Supplementary Figure 1** for the N-terminal signal peptide sequence shown (based on Clark et al.[1]) for the mitochondrial GABA-T1 (in blue) and the plastidic GABA-T3 (in green). These signal peptide regions were removed to generate corresponding GABA-T1 or GABA-T3 truncated versions. The results for each truncated GABA-T variant are representative of at least two independent experiments. **a.** Sub-cellular localisation of GABA-T1 truncated variant. **b.** Sub-cellular localisation of GABA-T3 truncated variant.

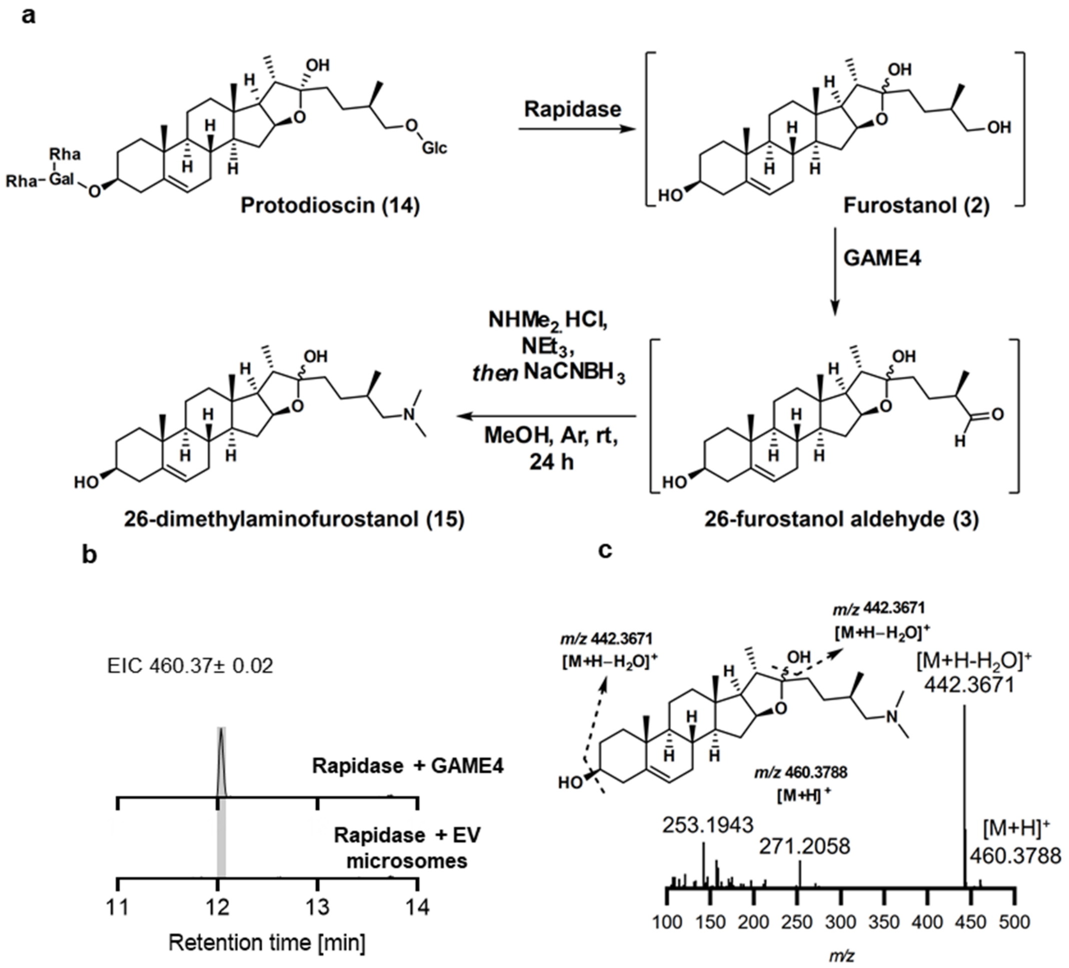

Supplementary Figure 6. The trapping of 26-furostanol aldehyde (3) *via* reductive amination. a. Schematic representation of the reductive amination of 26-furostanol aldehyde (**3**). **b.** EIC of the trapping experiment product (**15**). **c.** MS^2 spectrum of reductive amination product 26-dimethylamino furostanol (**15**). EIC; extracted ion chromatogram.

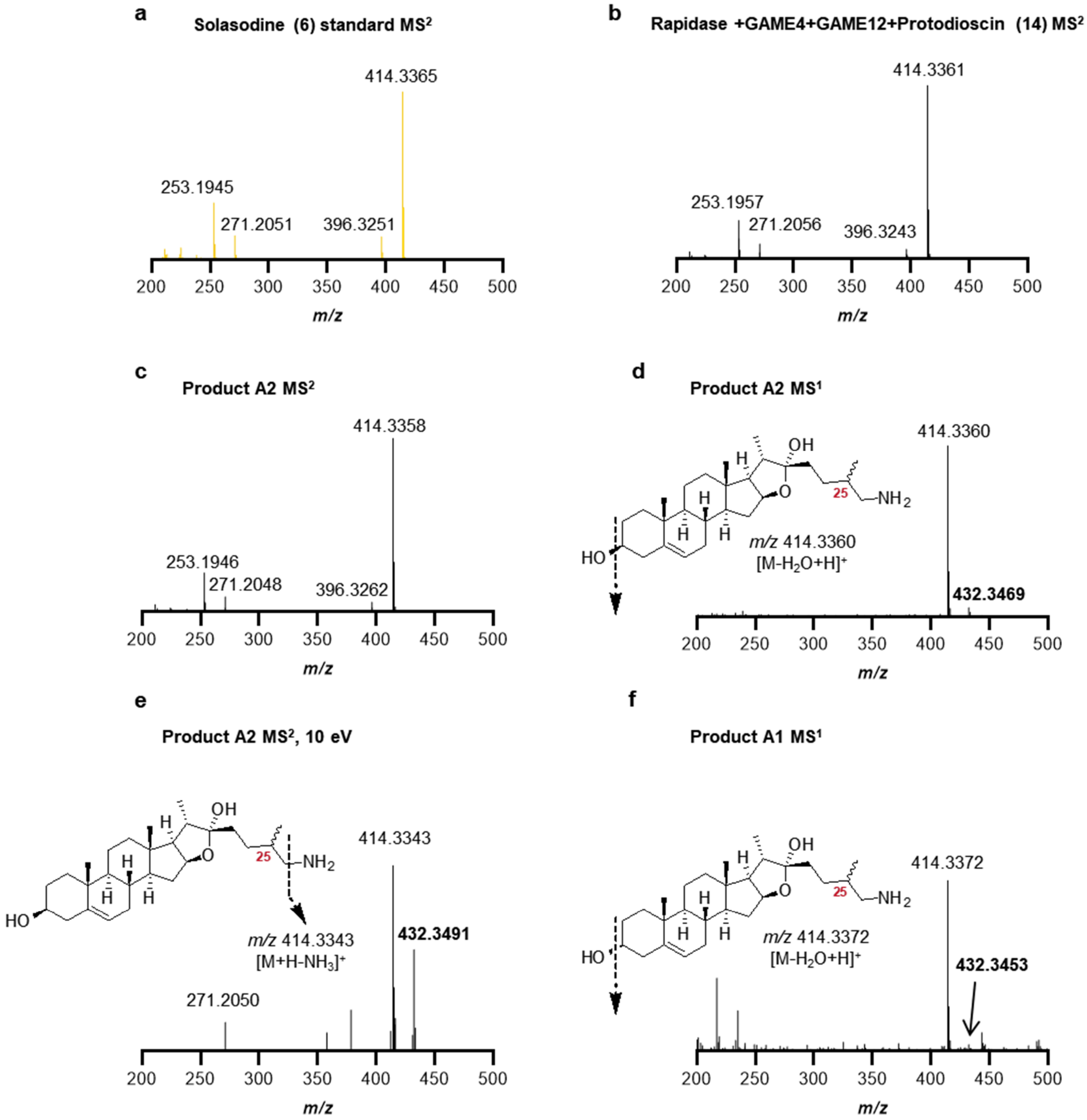

Supplementary Figure 7. The MS spectra of the products observed in coupled *in vitro* assays with protodioscin (14) and the corresponding standards. a. MS^2 spectrum of the solasodine **(6)** standard. **b**. MS^2 spectrum of the *in vitro* coupled assay product using protodioscin **(14)** as a substrate. **c**. MS^2 spectrum of the *in vitro* coupled assay product A2. **d.** The MS^1 spectrum of Product A2 detected in coupled *in vitro* assays of Rapidase, GAME4 and GAME12 on protodioscin **(14)**. **e.** The MS^2 spectrum of Product A2 at 10 eV collision energy. **f.** The MS^2 spectrum acquired of Product A1 detected in reconstitution based assays of GAME12 in *N. benthamiana*.

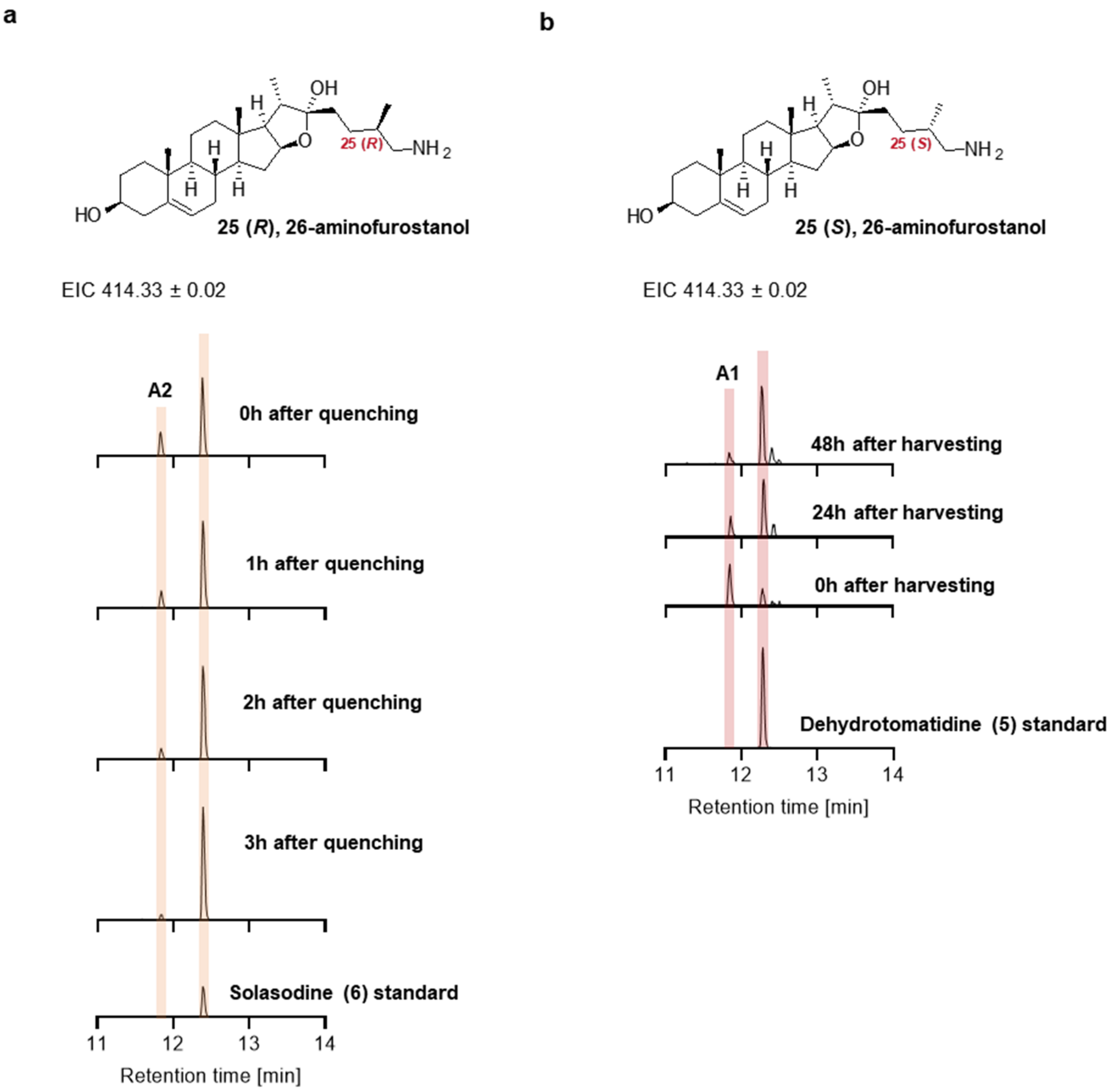

Supplementary Figure 8. The disappearance of Product A1 and Product A2, putatively assigned as 25(*R*) and 25(*S*) epimers of 26-aminofurostanol (4), the uncyclized intermediate of solasodine (6) and dehydrotomatidine (5). a. EICs of the coupled *in vitro* assay products incubated at room temperature for 0 to 3h after quenching of the reaction and precipitation of the enzymes, using protodioscin **(16)** as a substrate. **b.** EICs of the products of reconstitution assays in *N. benthamiana*, incubated at room temperature for 0 to 48h after extraction.

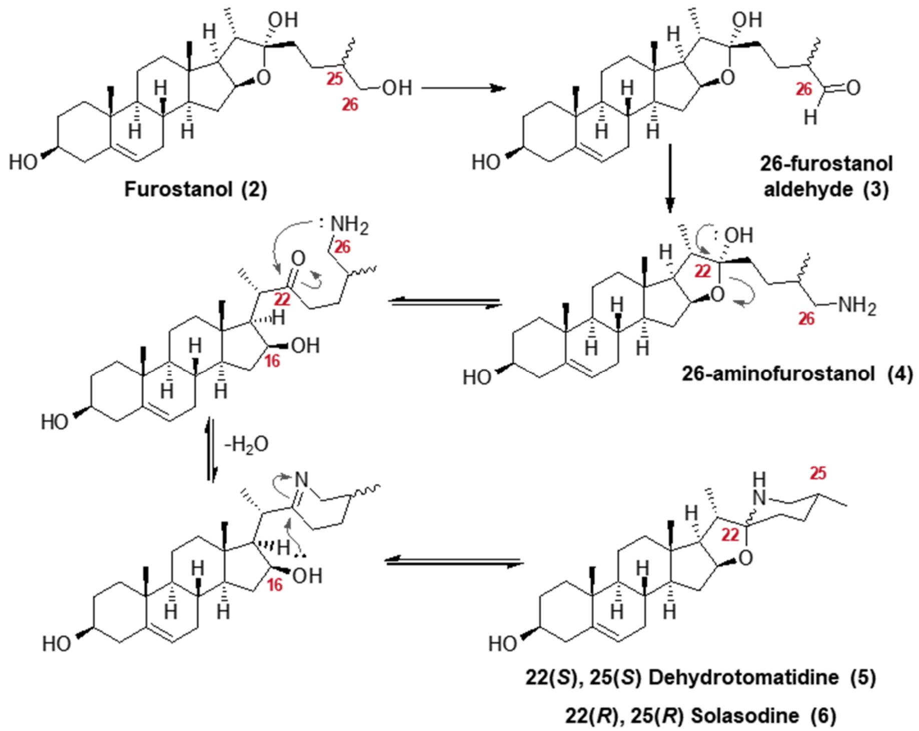

Supplementary Figure 9. The proposed mechanism of spriosolane SGA aglycone F-ring formation, based on the results of the *in vitro* assays, trapping of the 26-furostanol aldehyde (3) and previous labelling studies. The primary alcohol at C-26 of furostanol **(2)** is first oxidized in the presence of GAME4 to generate 26-furostanol aldehyde **(3)**. Following the GAME12-catalyzed transamination of 26-furostanol aldehyde **(3)** to give 26-aminofurostanol **(4)**, ring-opening of the cyclic hemiacetal at C-22 later reveals the corresponding ketone which can then subsequently react intramolecularly with the primary amino functionality at C-26 through a 6-exo-trig cyclization. Further intramolecular nucleophilic attack of the electrophilic cyclic imine motif by the adjacent C-16 hydroxy group generates the spiro carbinolamine ether scaffold of dehydrotomatidine **(5)** or solasodine **(6)**, depending on the C-25 stereochemistry of the precursor.

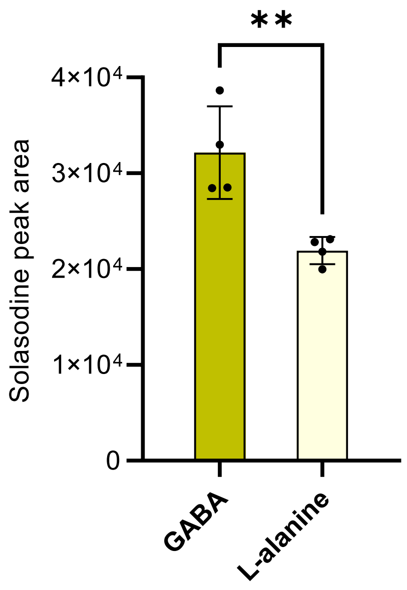

Supplementary Figure 10. Comparison of the peak area of the solasodine product of *in vitro* GAME12 assays using either GABA (12) or L-alanine (11) as an amino donor. The assays using either amino donor were performed in 4 replicates (n=4). Data are displayed as mean ± SD. Unpaired, two-tailed t-test analysis showed a significant difference between the peak areas produced when using the two different amino donors (p = 0.0066).

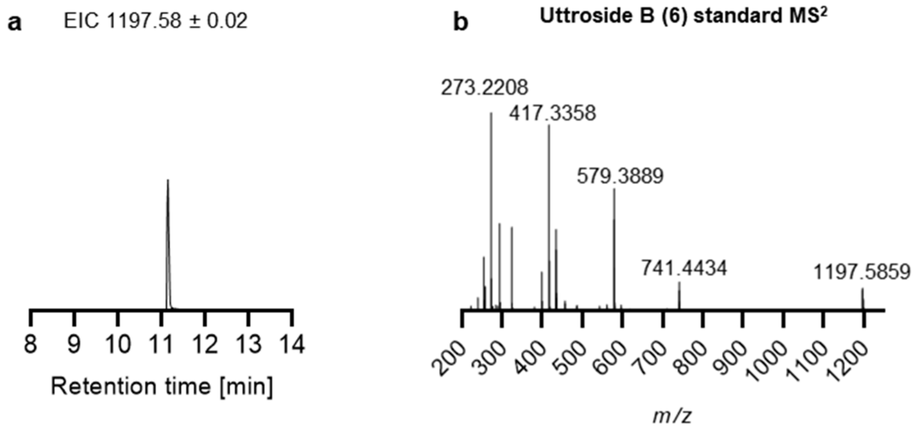

Supplementary Figure 11. The UHPLC-MS data of uttroside B (16) isolated from *S. nigrum* leaves. **a.** EIC of uttroside B (**16**) purified from the wildtype *S. nigrum* leaves. **b.** MS^2 spectrum of the purified uttroside B.

a

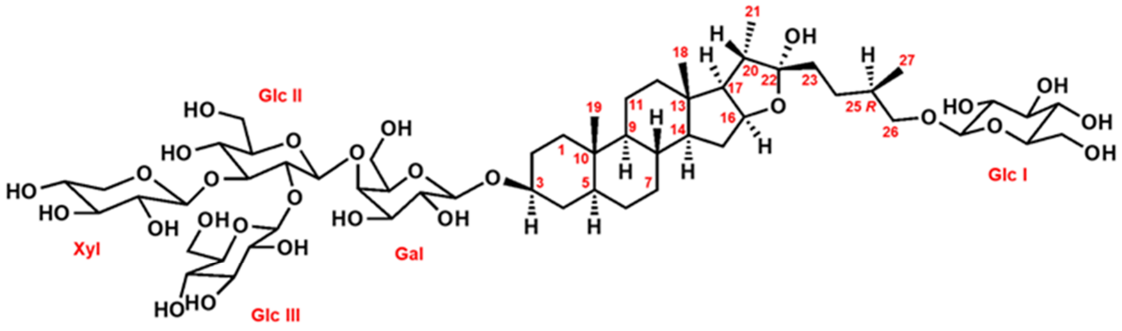

b

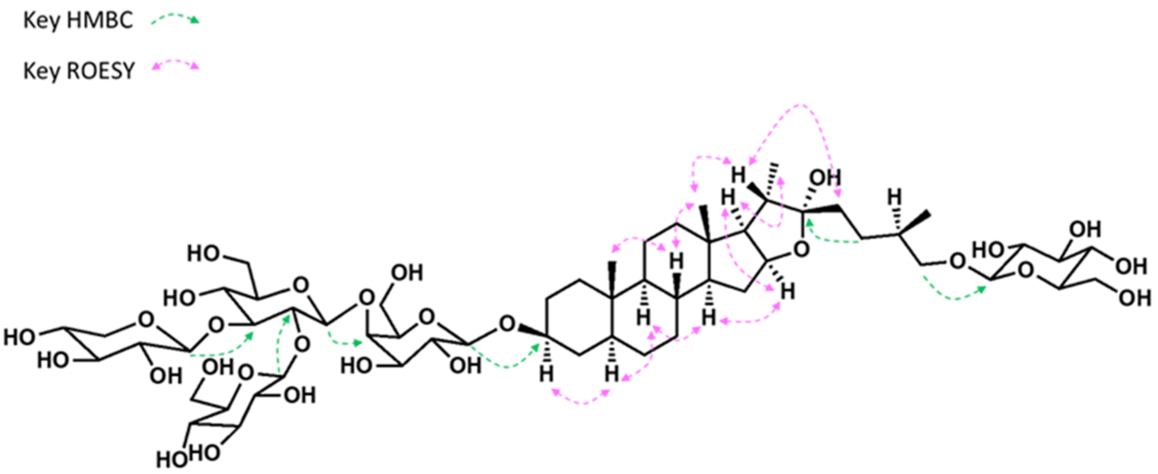

Supplementary Figure 12. a. Chemical structure of uttroside B (16). The stereochemistry of C25 is marked. **b.** Key HMBC and ROESY correlations of uttroside B (**16**).

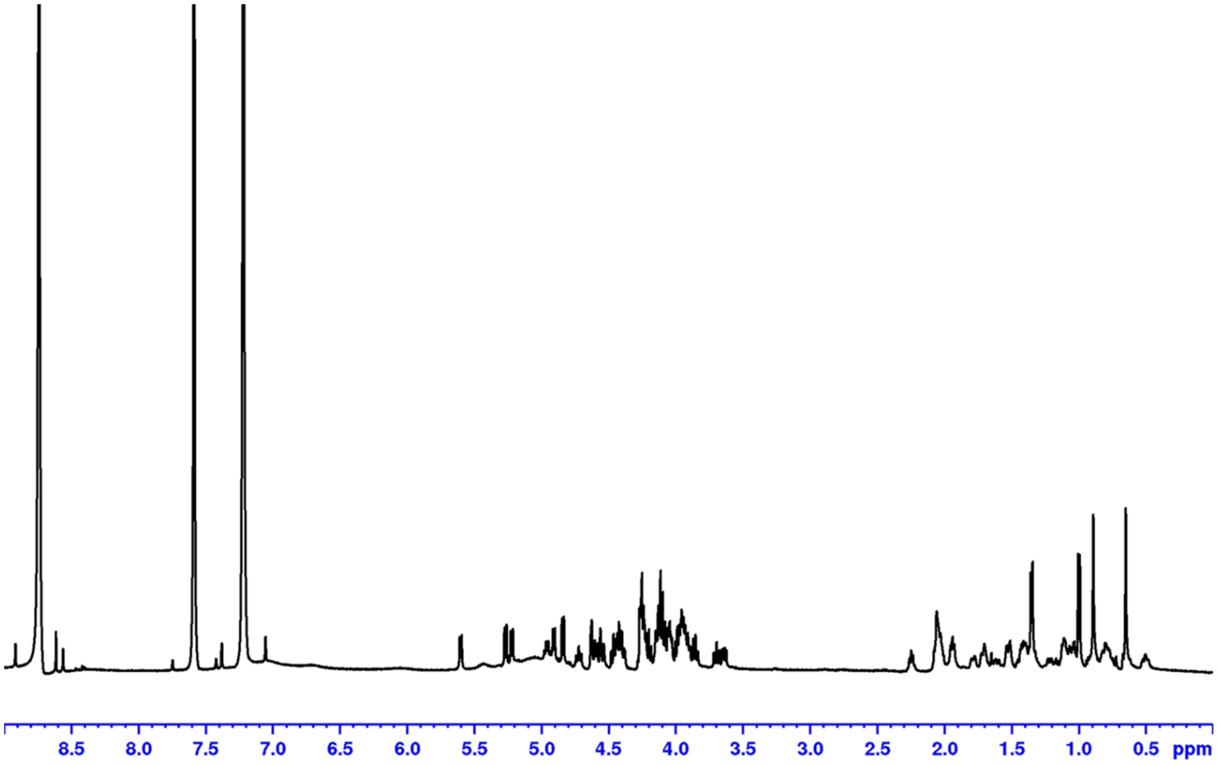

Supplementary Figure 13. Uttroside B (16), ^{1}H NMR, full range, in pyridine-*d*5.

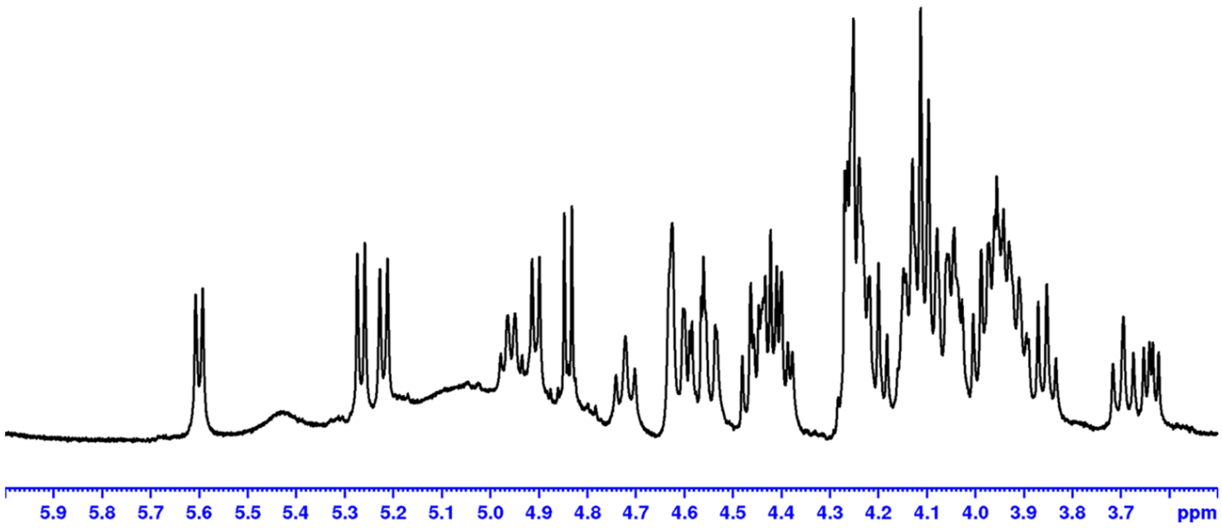

Supplementary Figure 14. Uttroside B (16), ^{1}H NMR, sugar range, in pyridine-*d*5.

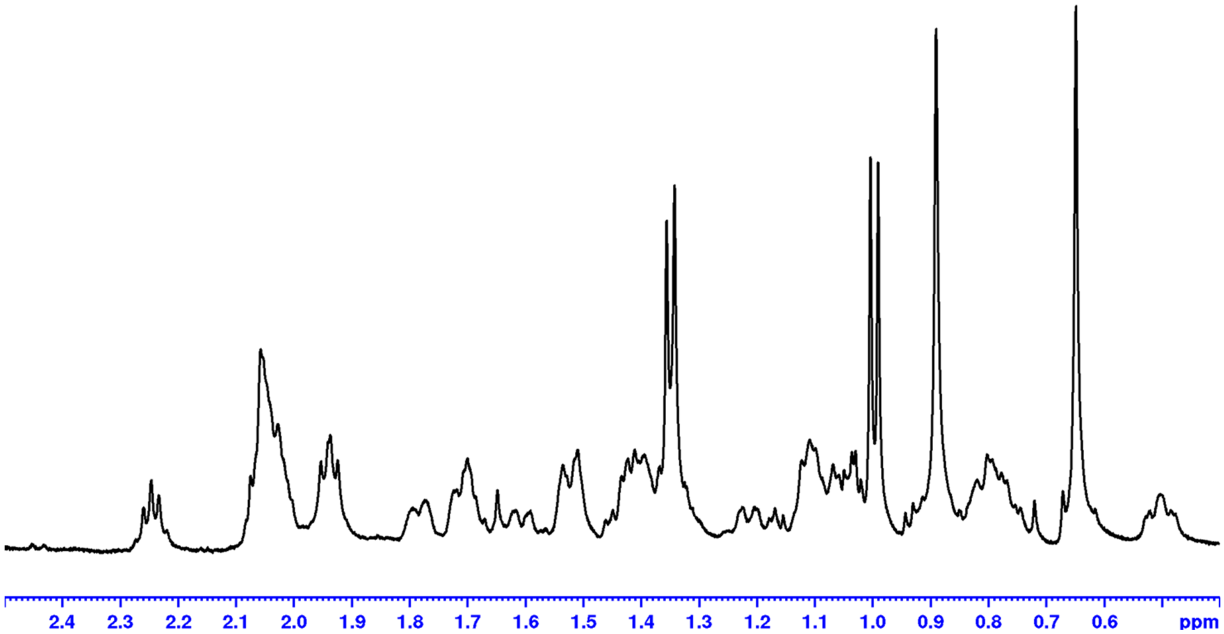

Supplementary Figure 15. Uttroside B (16), ^{1}H NMR, aglycone range, in pyridine-*d*$_5$.

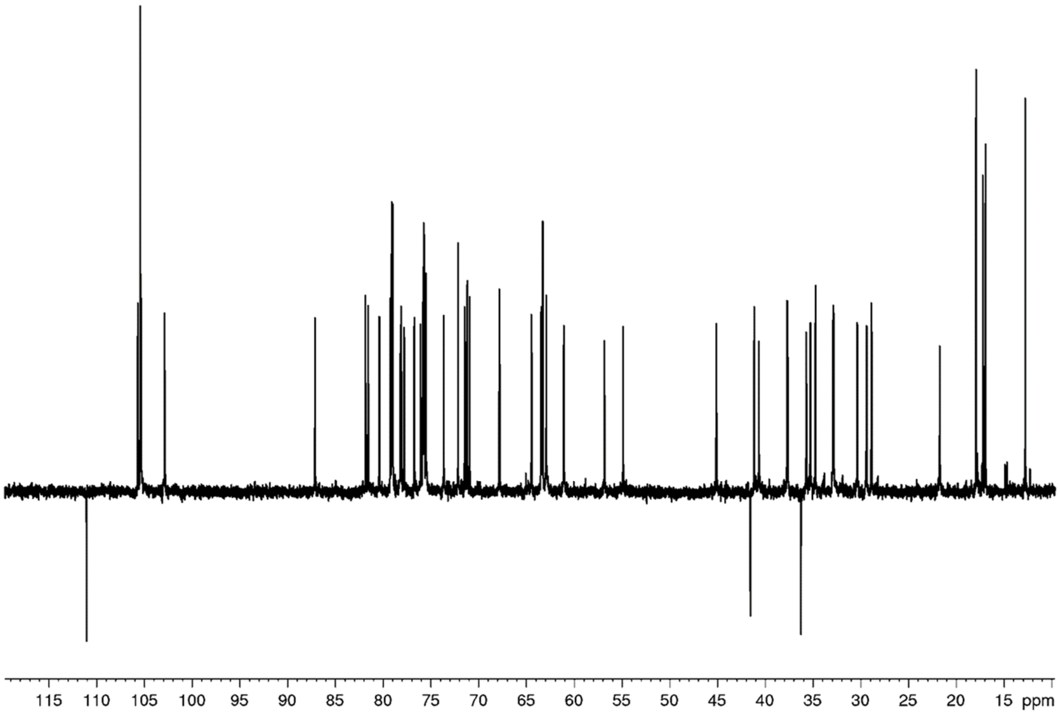

Supplementary Figure 16. Uttroside B (16), DEPTQ in pyridine-*d*5.

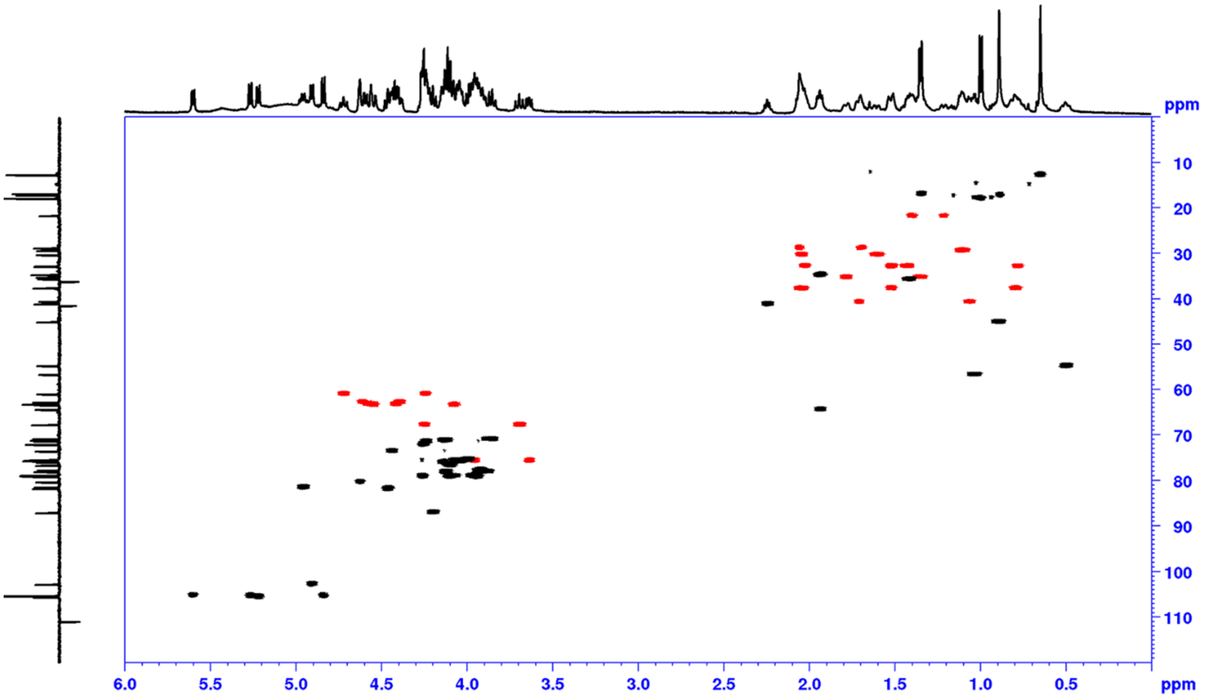

Supplementary Figure 17. Uttroside B (16), phase sensitive HSQC, full range, in pyridine-*d*5.

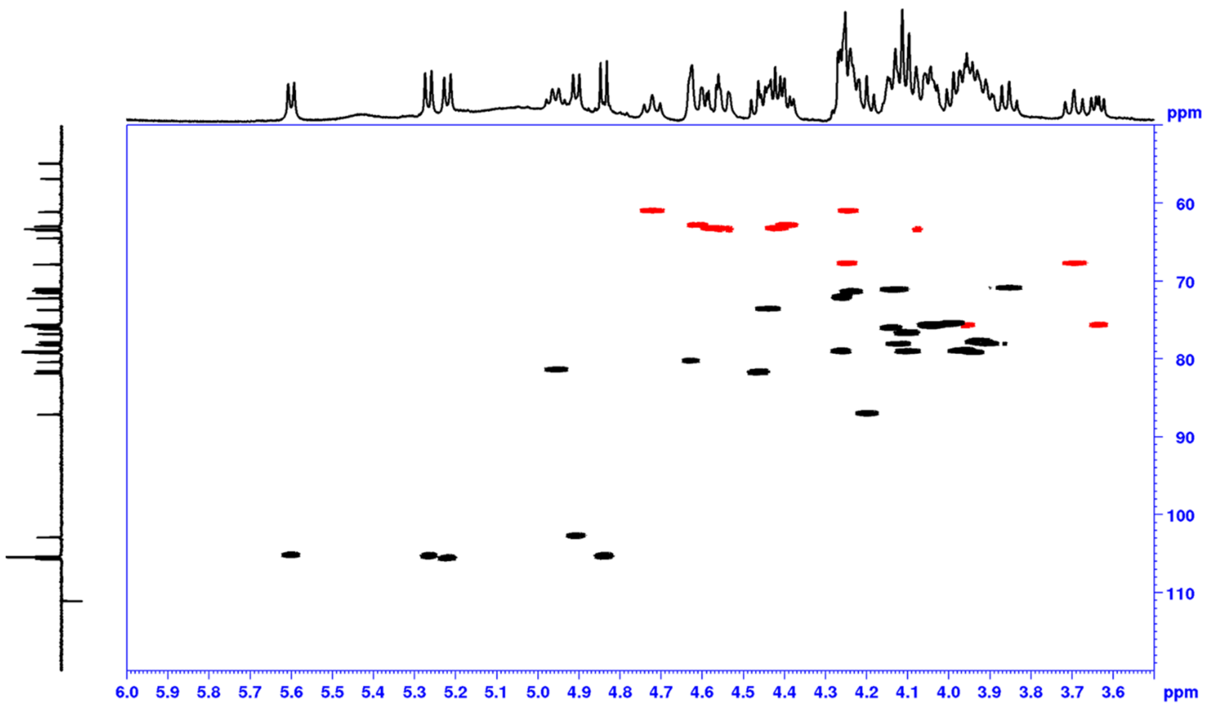

Supplementary Figure 18. Uttroside B (16), phase sensitive HSQC, sugar range, in pyridine-*d*5.

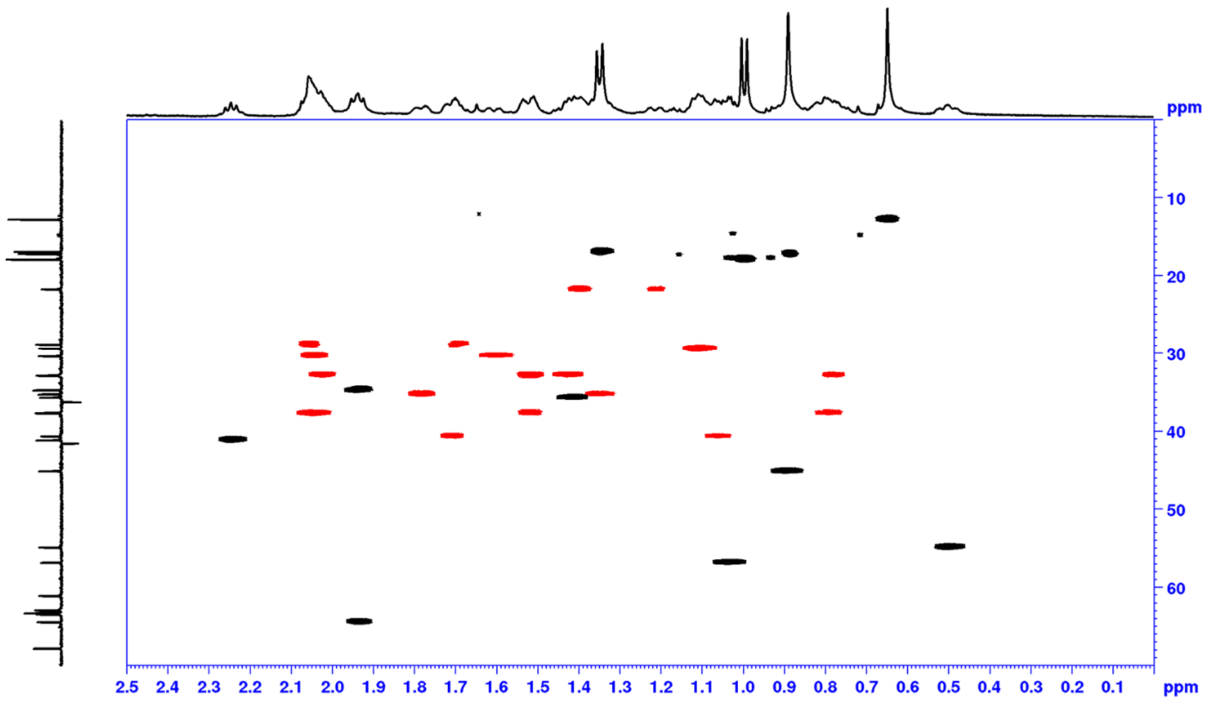

Supplementary Figure 19. Uttroside B (16), phase sensitive HSQC, aglycone range, in pyridine-*d*5.

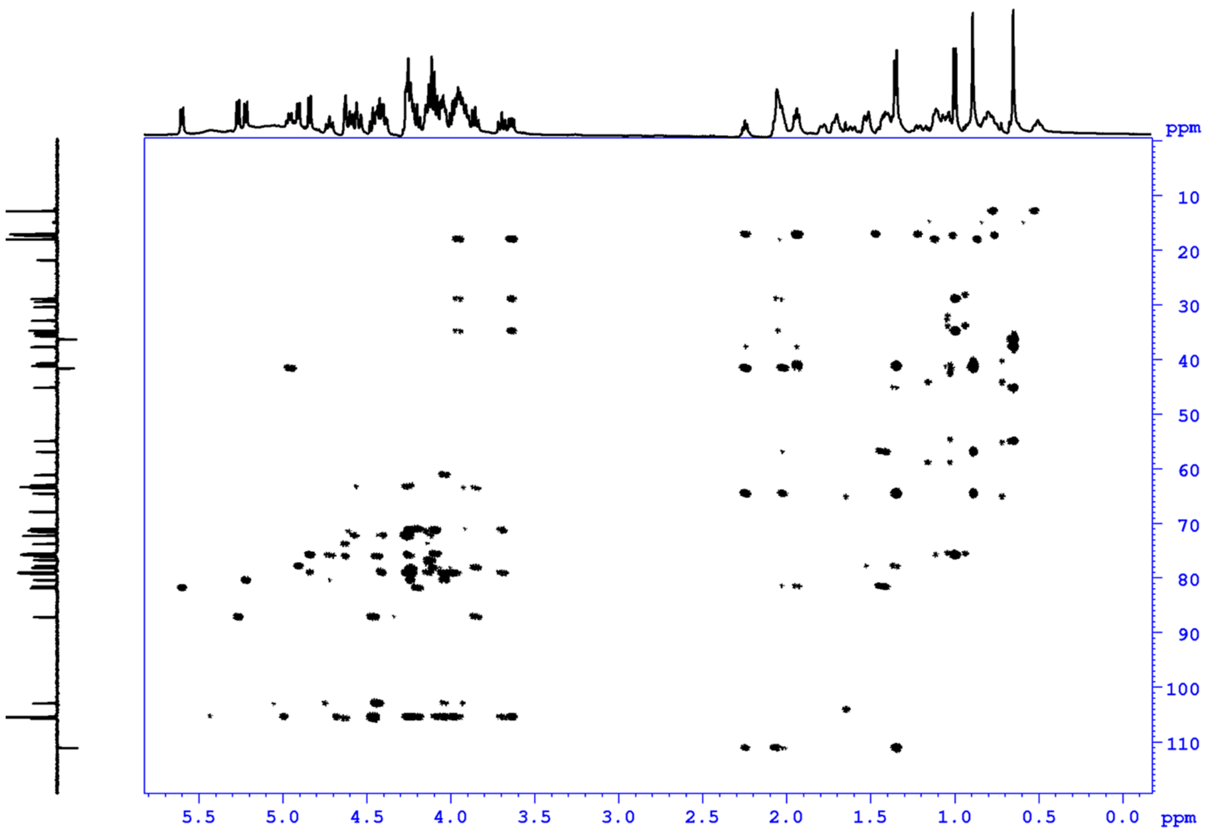

Supplementary Figure 20. Uttroside B (16), HMBC, full range, in pyridine-*d*$_5$.

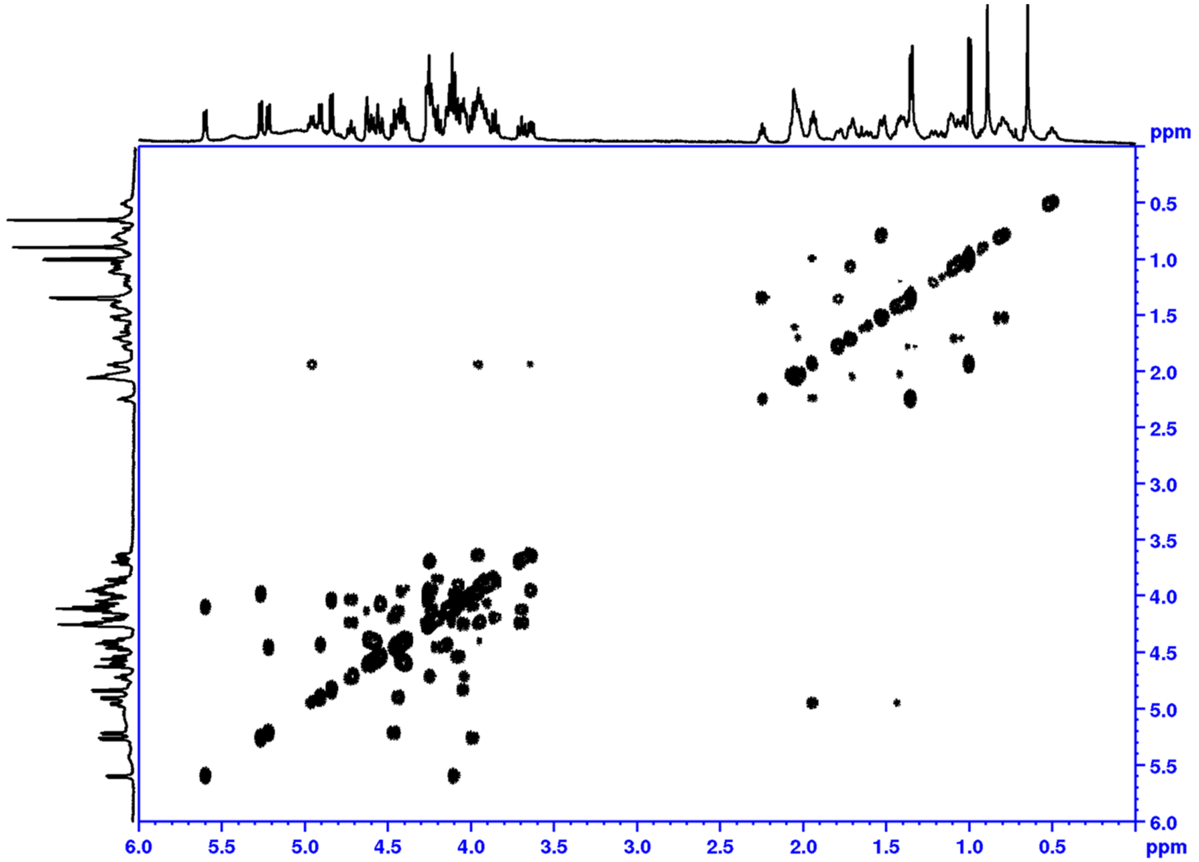

Supplementary Figure 21.Uttroside B (16), gradient-selected DQFCOSY, full range, in pyridine-*d*₅.

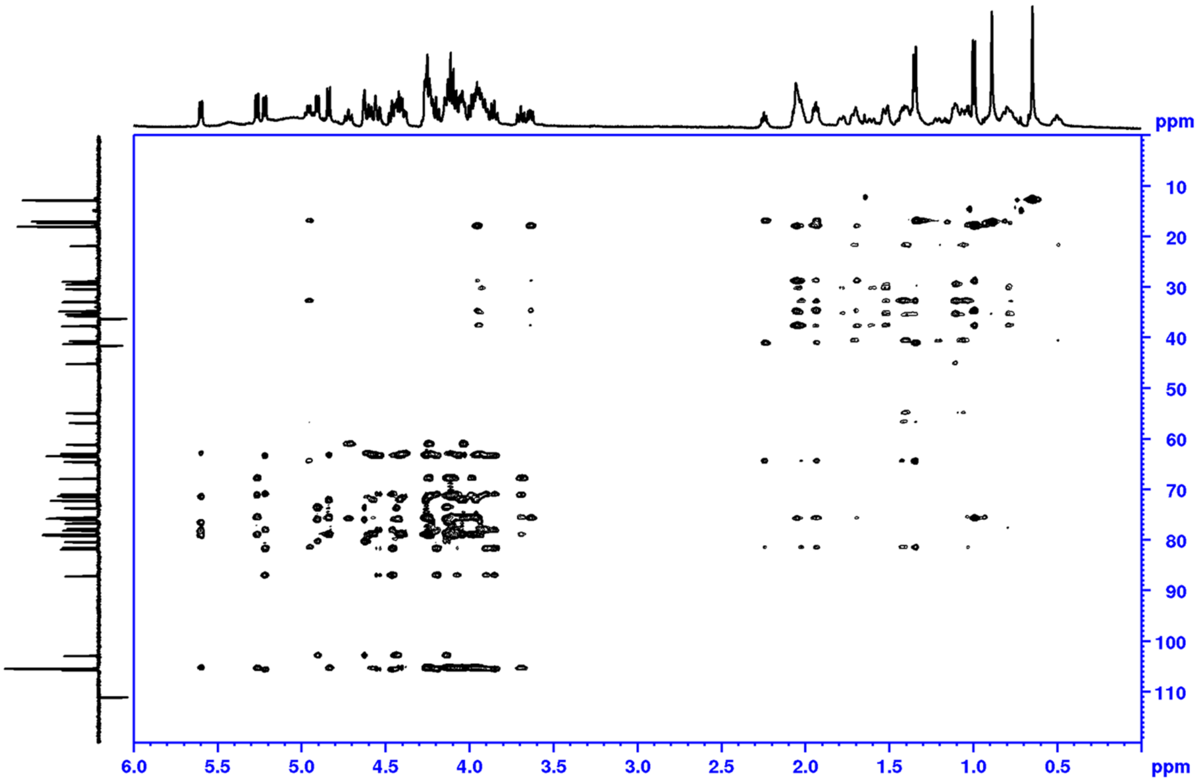

Supplementary Figure 22. Uttroside B (16), HSQC-TOCSY, full range, in pyridine-*d*₅.

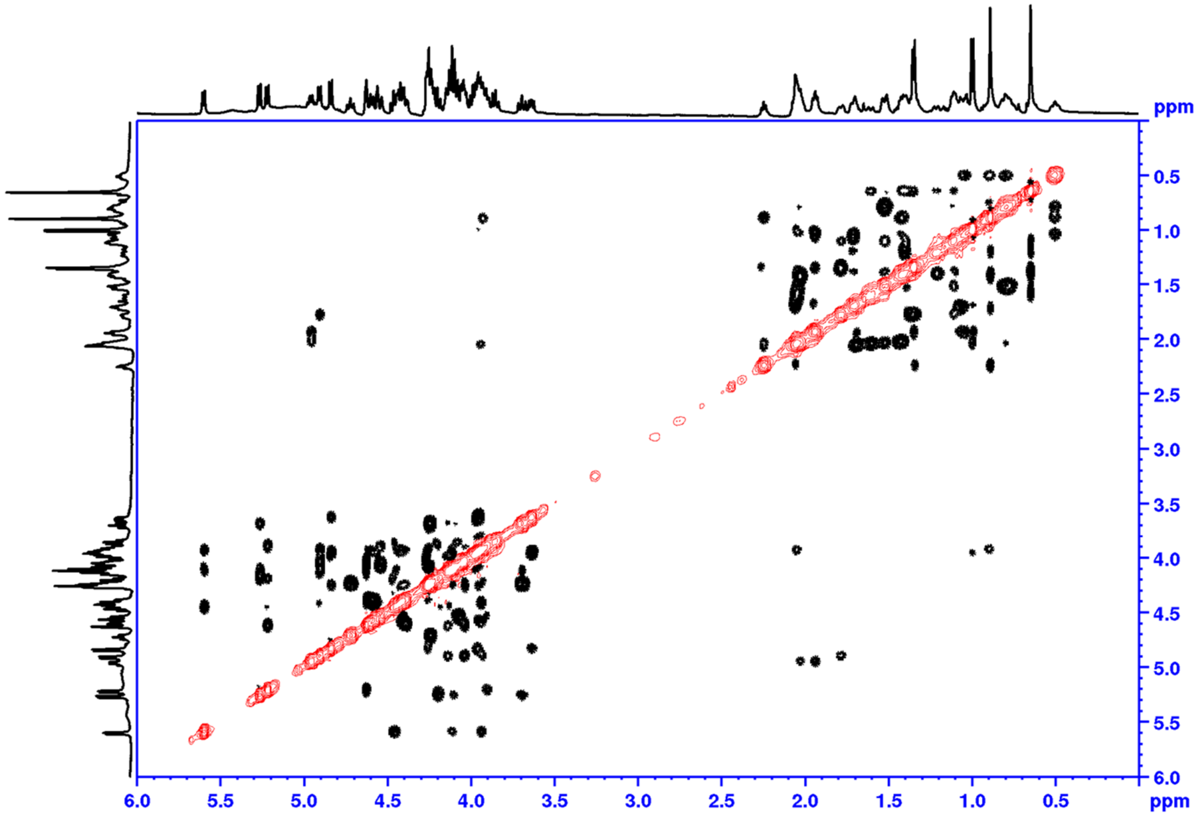

Supplementary Figure 23. Uttroside B (16), ROESY, full range, in pyridine-*d*$_5$.

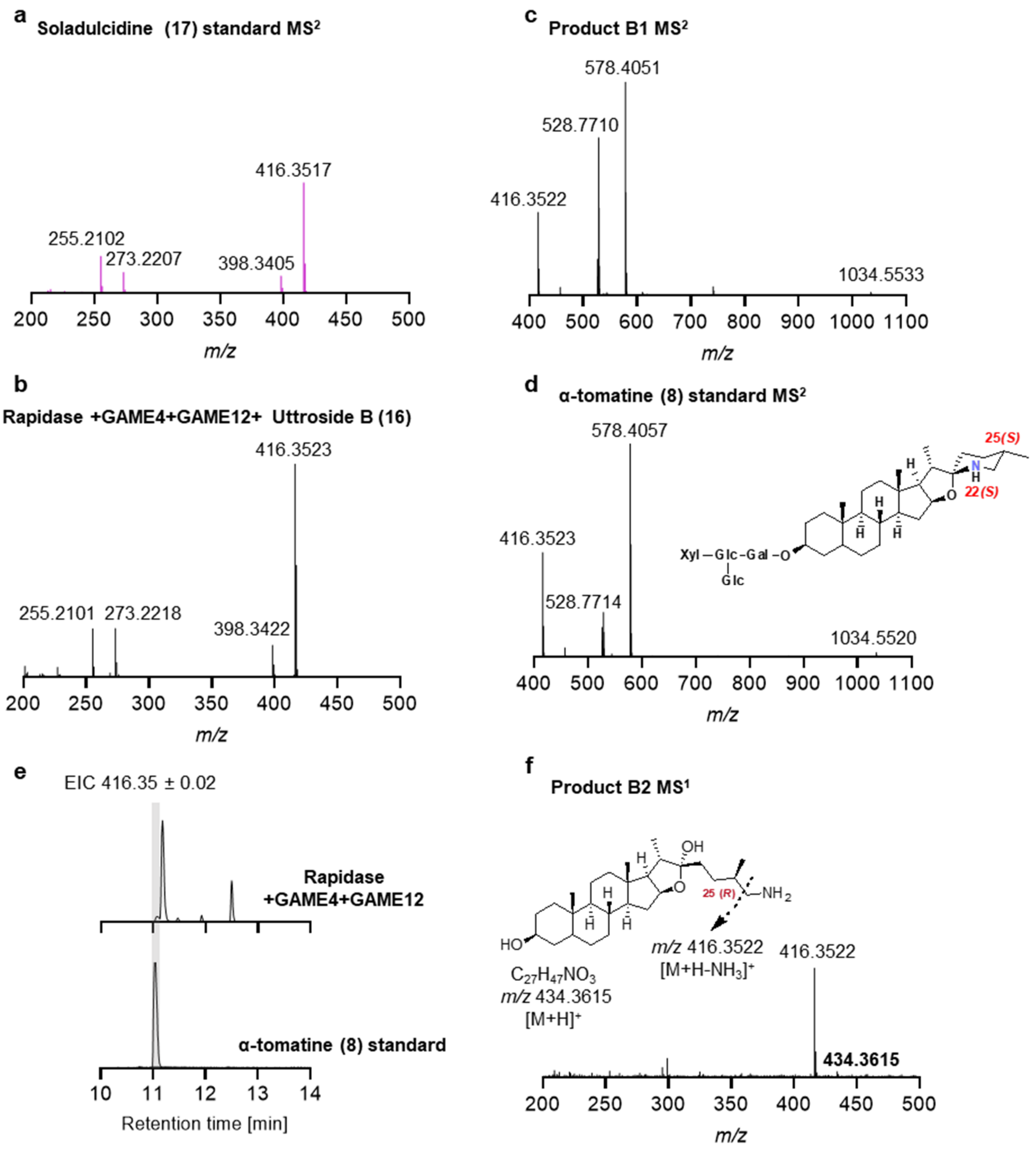

Supplementary Figure 24. The MS^2 spectra of the products obtained by *in vitro* coupled assays with uttroside B (16) and the corresponding standards. a. MS^2 spectrum of the soladulcidine **(17)** standard. **b**. MS^2 spectrum of the *in vitro* coupled assay product on uttroside B **(16) c.** MS^2 spectrum of product B. **d.** MS^2 spectrum of the α-tomatine **(8)** standard. **e.** EICs of the products of coupled *in vitro* assays on uttroside B **(16)** and the α-tomatine **(8)** standard. **f.** The MS^1 spectrum of Product B2 detected in coupled *in vitro* assays of Rapidase, GAME4 and GAME12 on uttroside B **(16)**.

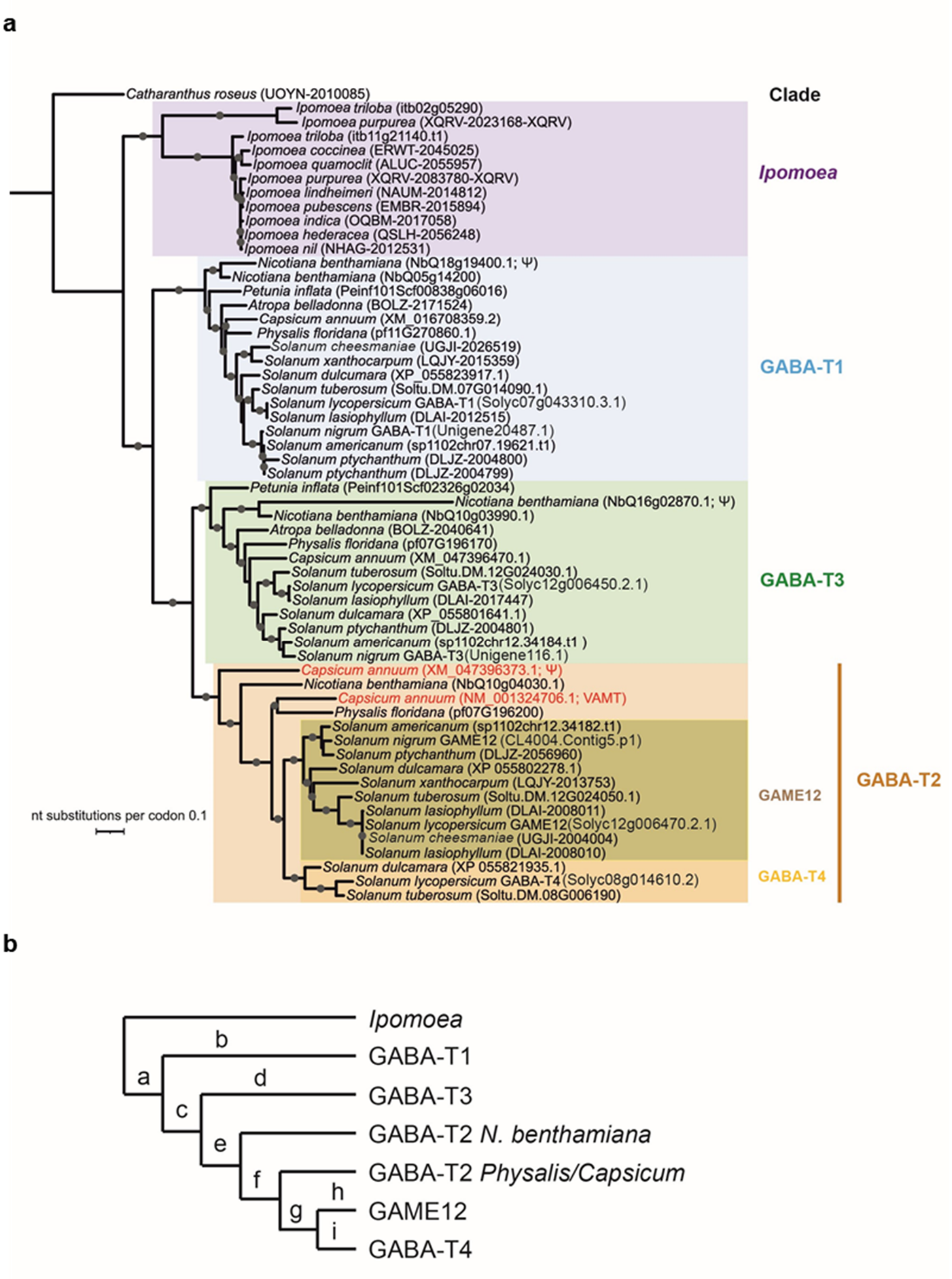

Supplementary Figure 25. Extended *GABA-T* phylogeny. a. *GABA-T* phylogeny with pseudogenes included (ψ). The *C. annuum GABA-T2* pseudogene and *VAMT* are marked in red. Grey circles show branches with >80% and >95% support as judged by 1000X SH-aLRT and UltraFast Boostrapping replicates, respectively. The long branches in the early diverging *GABA-T2* sequences make the topology in this region somewhat unstable. See **Extended Data Table 2** for gene models used. **b.** Simplified topology of *GABA-T* tree to highlight branches tested for diversifying selection (see **Supplementary Table 3**).

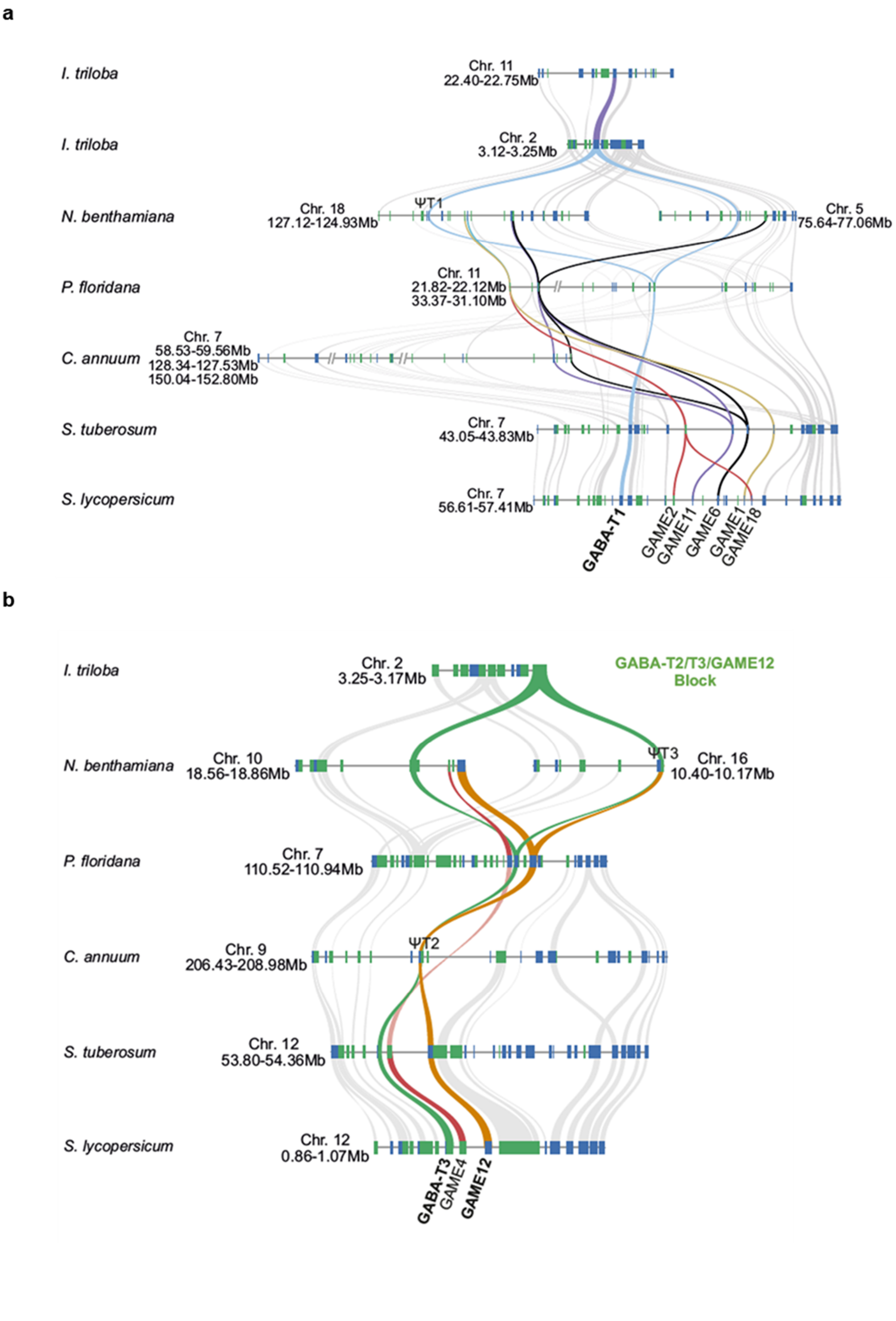
a
I. triloba
Chr. 11
22.40-22.75Mb
I. triloba
Chr. 2
3.12-3.25Mb
N. benthamiana
Chr. 18
127.12-124.93Mb
ΨT1
Chr. 5
75.64-77.06Mb
P. floridana
Chr. 11
21.82-22.12Mb
33.37-31.10Mb
C. annuum
Chr. 7
58.53-59.56Mb
128.34-127.53Mb
150.04-152.80Mb
S. tuberosum
Chr. 7
43.05-43.83Mb
S. lycopersicum
Chr. 7
56.61-57.41Mb
GABA-T1
GAME2
GAME11
GAME6
GAME1
GAME18
b
I. triloba
Chr. 2
3.25-3.17Mb
GABA-T2/T3/GAME12
Block
N. benthamiana
Chr. 10
18.56-18.86Mb
ΨT3
Chr. 16
10.40-10.17Mb
P. floridana
Chr. 7
110.52-110.94Mb
C. annuum
Chr. 9
206.43-208.98Mb
ΨT2
S. tuberosum
Chr. 12
53.80-54.36Mb
S. lycopersicum
Chr. 12
0.86-1.07Mb
GABA-T3
GAME4
GAME12

c

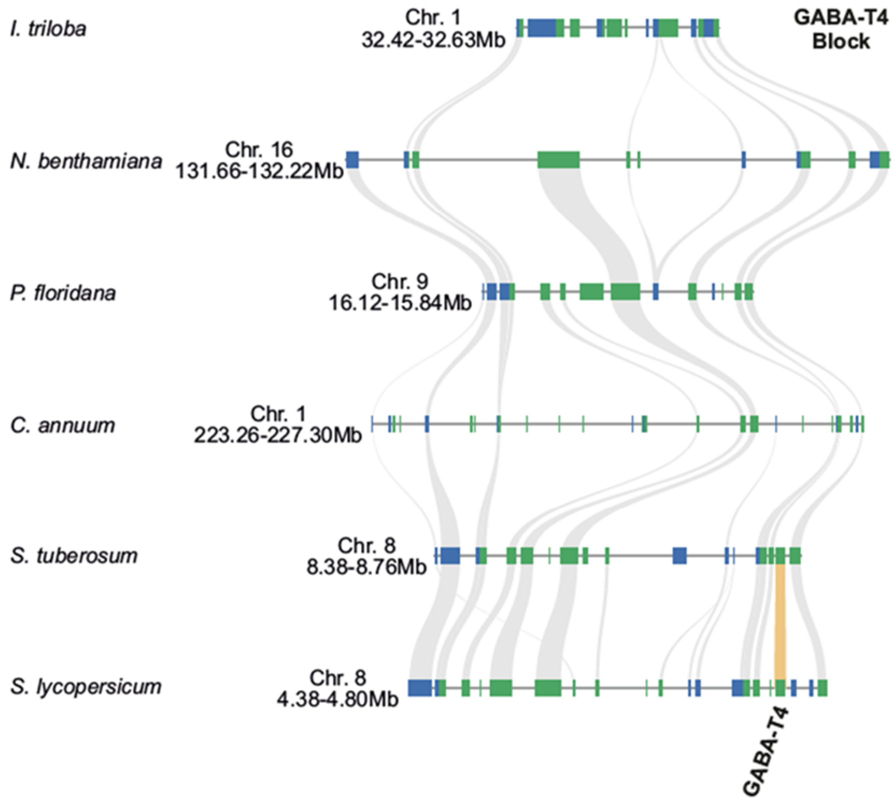

d

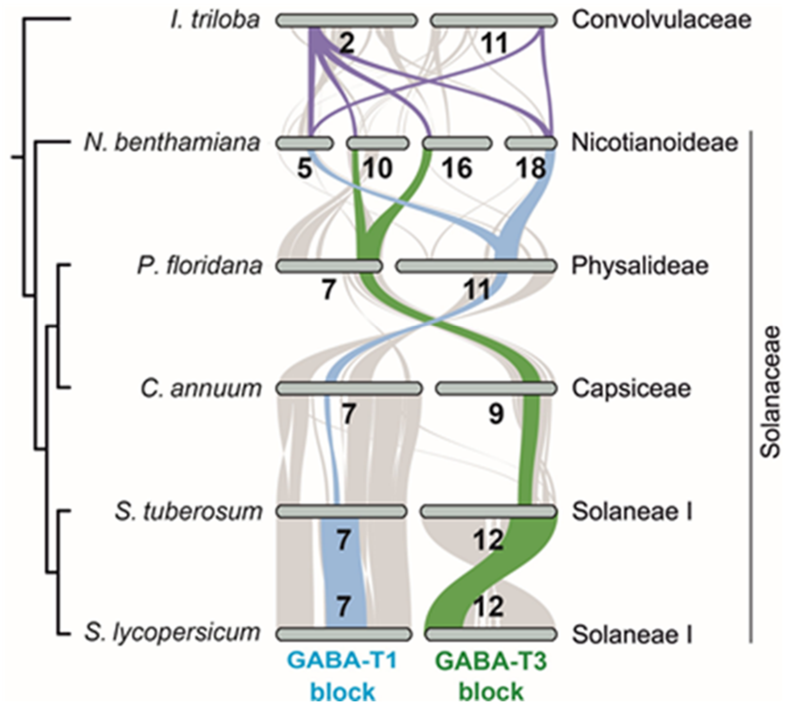

Supplementary Figure 26. Chromosome-level microsynteny analysis of GABA-T1, GABA-T3 and GABA-T4 (a, b, c) and macrosynteny (d) analysis of GABA-T1 GABA-T3 syntenic blocks. Chromosome numbers are depicted. The cladogram shows the species relationships inferred by Orthofinder.

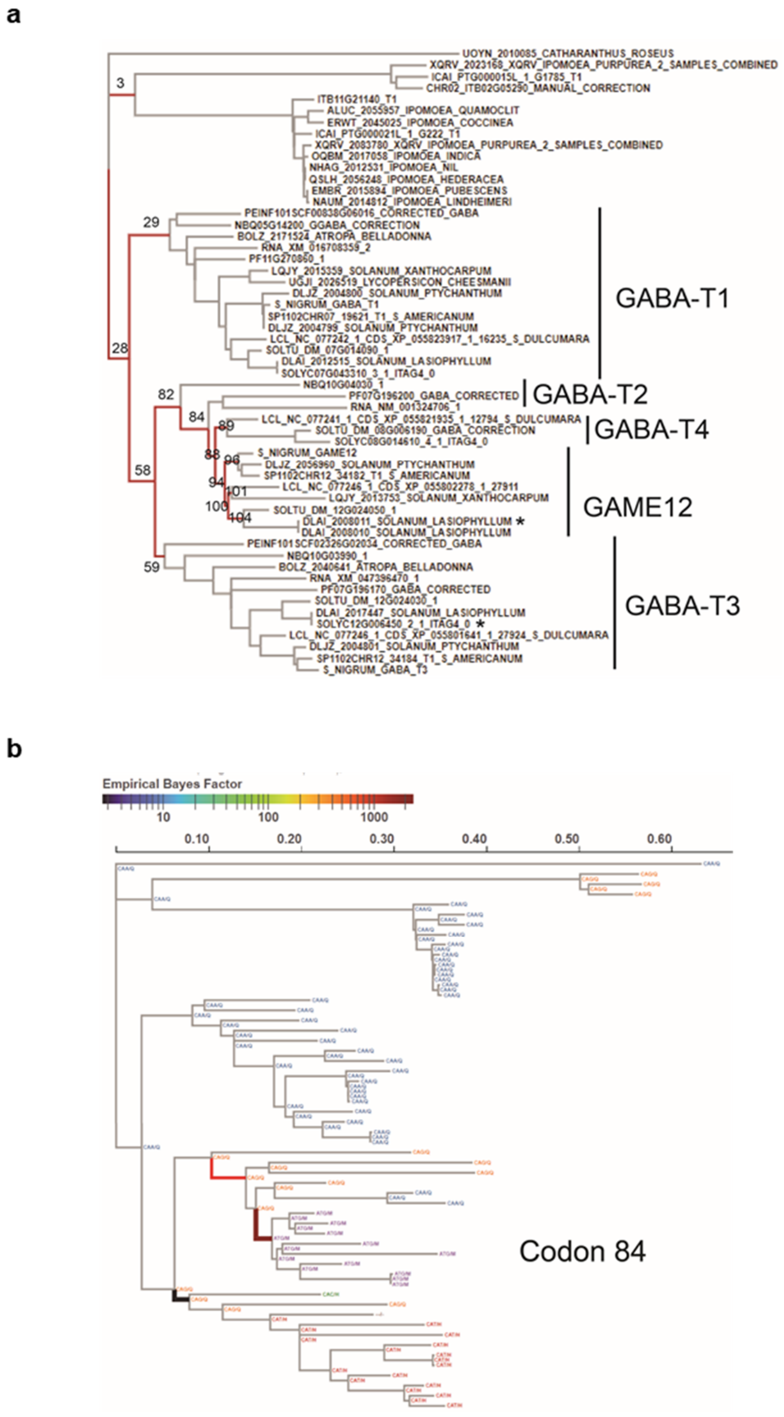

Supplementary Figure 27. Site-specific diversifying selection tests. a. BUSTED phylogeny with branches for selection tests highlighted in red and labelled with number. Stars show the position of S. *lycopersicum* GABA-T3 and GAME-12 (note: although *S. lasiophyllum* GAME12 is depicted, this is identical to *S. lycopersicum* GAME12). **b.** Example of codon under selection (codon 84) showing branch-site Empirical Bayes Factor test superimposed onto phylogeny with codons/amino acids labelled at tips and nodes.

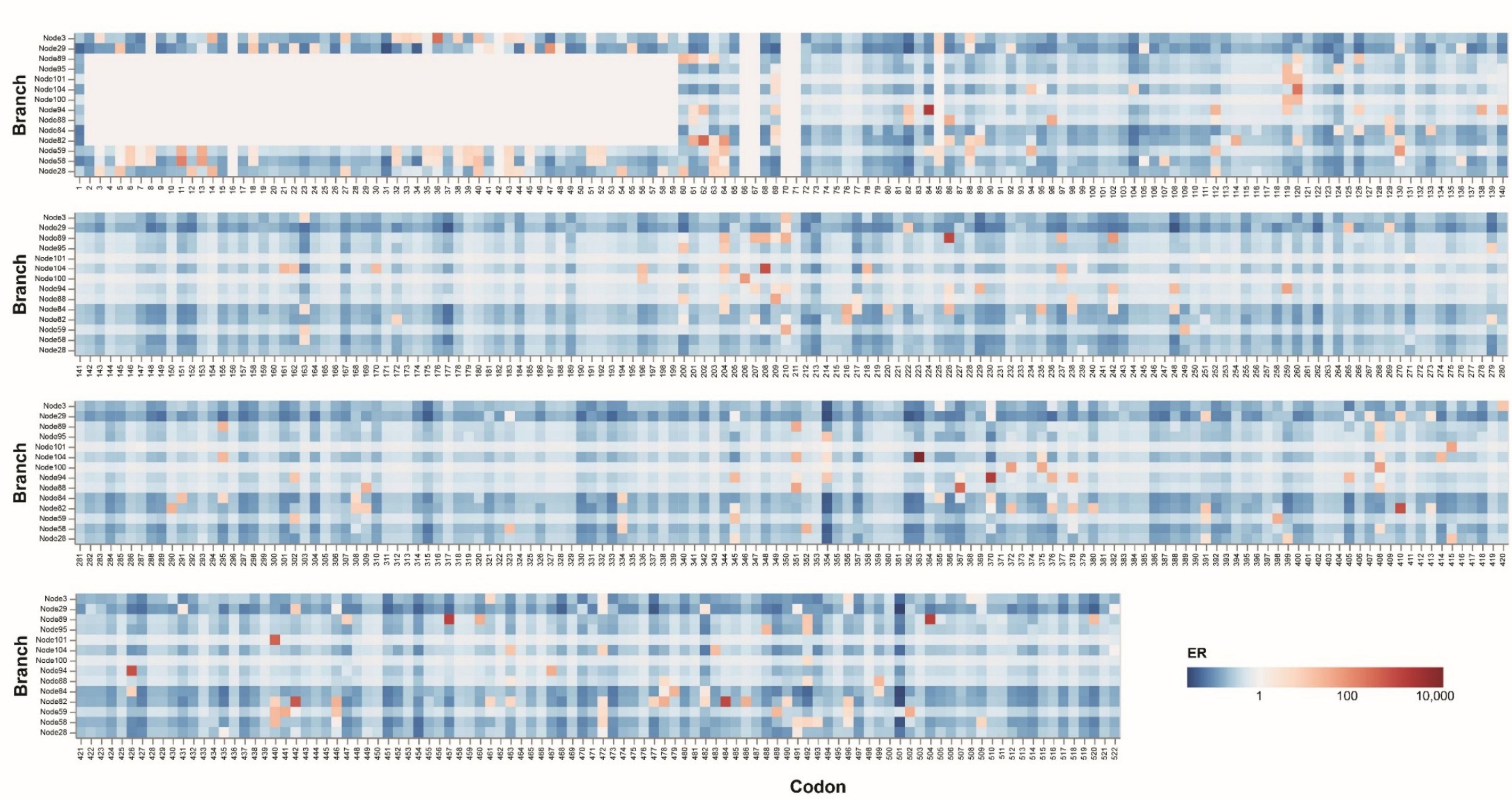

Supplementary Figure 28. Complete branch-site Empirical Bayes Factors for selected branches (labelled here as nodes in rows) across all codons.

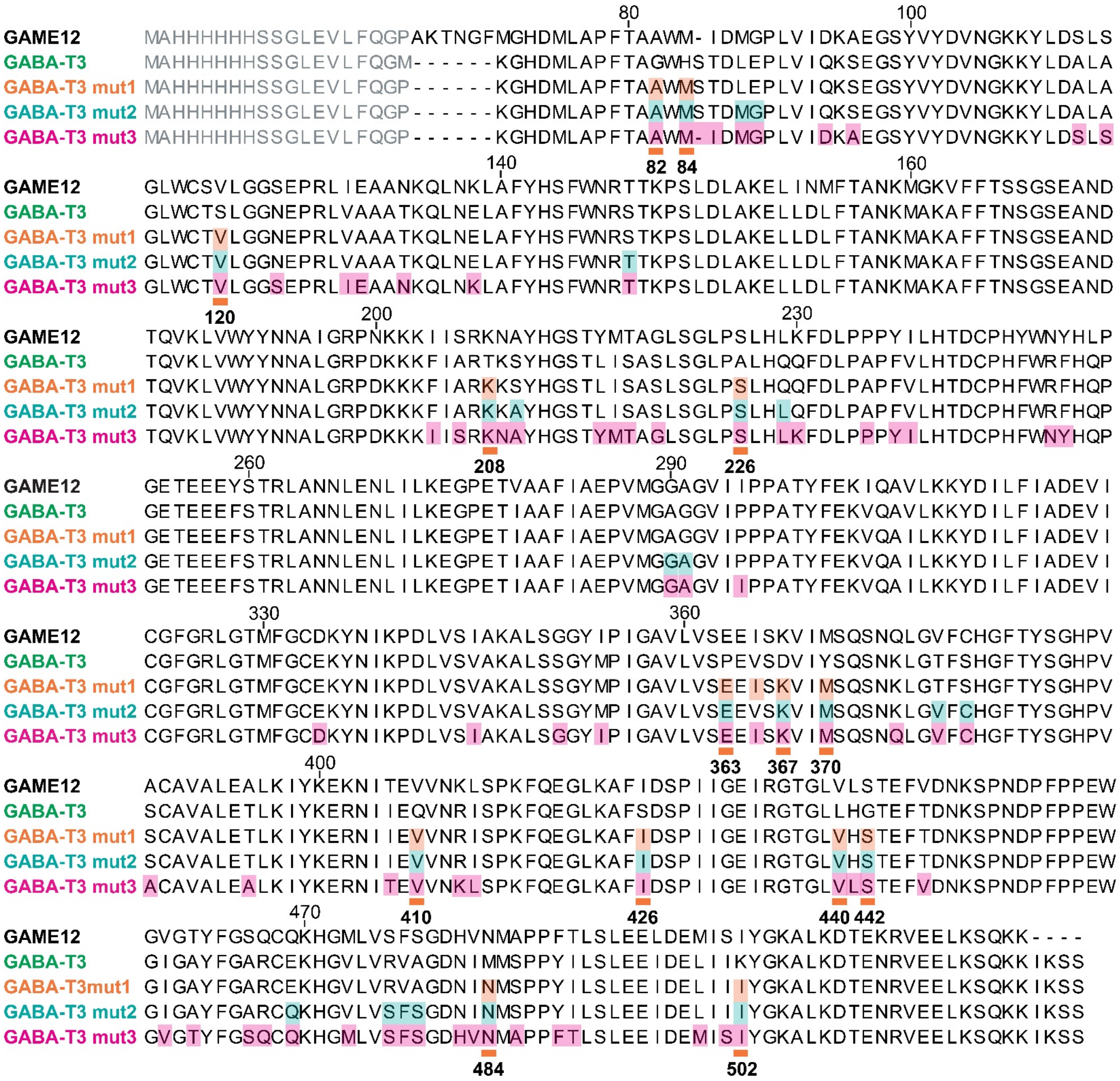
80
100
GAME12 MAHHHHHHSSGLEVLFQGPAKTNGFMGHDMLAPFTAAWM-IDMGPLVIDKAEGSYVYDVNGKKYLDSLS
GABA-T3 MAHHHHHHSSGLEVLFQGM------KGHDMLAPFTAGWHSTDLEPLVIQKSEGSYVYDVNGKKYLDALA
GABA-T3 mut1 MAHHHHHHSSGLEVLFQGP------KGHDMLAPFTAAWMSTDLEPLVIQKSEGSYVYDVNGKKYLDALA
GABA-T3 mut2 MAHHHHHHSSGLEVLFQGP------KGHDMLAPFTAAWMSTDMGPLVIQKSEGSYVYDVNGKKYLDALA
GABA-T3 mut3 MAHHHHHHSSGLEVLFQGP------KGHDMLAPFTAAWM-IDMGPLVIDKAEGSYVYDVNGKKYLDSLS
82 84
140
160
GAME12 GLWCSVLGGSEPRLIEAANKQLNKLAFYHSFWNRTTKPSLDLAKELINMFTANKMGKVFFTSSGSEAND
GABA-T3 GLWCTSLGGNEPRLVAAATKQLNELAFYHSFWNRSTKPSLDLAKELLDLFTANKMAKAFFTNSGSEAND
GABA-T3 mut1 GLWCTVLGGNEPRLVAAATKQLNELAFYHSFWNRSTKPSLDLAKELLDLFTANKMAKAFFTNSGSEAND
GABA-T3 mut2 GLWCTVLGGNEPRLVAAATKQLNELAFYHSFWNRTTKPSLDLAKELLDLFTANKMAKAFFTNSGSEAND
GABA-T3 mut3 GLWCTVLGGSEPRLIEAANKQLNKLAFYHSFWNRTTKPSLDLAKELLDLFTANKMAKAFFTNSGSEAND
120
200
230
GAME12 TQVKLVWYYNNAIGRPNKKKIISRKNAYHGSTYMTAGLSGLPSLHLKFDLPPPYILHTDCPHYWNYHLP
GABA-T3 TQVKLVWYYNNALGRPDKKKFIARTKSYHGSTLISASLSGLPALHQQFDLPAPFVLHTDCPHFWRFHQP
GABA-T3 mut1 TQVKLVWYYNNALGRPDKKKFIARKKSYHGSTLISASLSGLPSLHQQFDLPAPFVLHTDCPHFWRFHQP
GABA-T3 mut2 TQVKLVWYYNNALGRPDKKKFIARKKAYHGSTLISASLSGLPSLHLQFDLPAPFVLHTDCPHFWRFHQP
GABA-T3 mut3 TQVKLVWYYNNALGRPDKKKIISRKNAYHGSTYMTAGLSGLPSLHLKFDLPPPYILHTDCPHFWNYHQP
208
226
260
290
GAME12 GETEEEYSTRLANNLENLILKEGPETVAAFIAEPVMGGAGVIIPPATYFEKIQAVLKKYDILFIADEVI
GABA-T3 GETEEEFSTRLANNLENLILKEGPETIAAFIAEPVMGAGGVIPPPATYFEKVQAVLKKYDILFIADEVI
GABA-T3 mut1 GETEEEFSTRLANNLENLILKEGPETIAAFIAEPVMGAGGVIPPPATYFEKVQAILKKYDILFIADEVI
GABA-T3 mut2 GETEEEFSTRLANNLENLILKEGPETIAAFIAEPVMGGAGVIPPPATYFEKVQAILKKYDILFIADEVI
GABA-T3 mut3 GETEEEFSTRLANNLENLILKEGPETIAAFIAEPVMGGAGVIIPPATYFEKVQAILKKYDILFIADEVI
330
360
GAME12 CGFGRLGTMFGCDKYNIKPDLVSIAKALSGGYIPIGAVLVSEEISKVIMSQSNQLGVFCHGFTYSGHPV
GABA-T3 CGFGRLGTMFGCEKYNIKPDLVSVAKALSSGYMPIGAVLVSPEVSDVIYSQSNKLGTFSHGFTYSGHPV
GABA-T3 mut1 CGFGRLGTMFGCEKYNIKPDLVSVAKALSSGYMPIGAVLVSEEISKVIMSQSNKLGTFSHGFTYSGHPV
GABA-T3 mut2 CGFGRLGTMFGCEKYNIKPDLVSVAKALSSGYMPIGAVLVSEEVSKVIMSQSNKLGVFCHGFTYSGHPV
GABA-T3 mut3 CGFGRLGTMFGCDKYNIKPDLVSIAKALSGGYIPIGAVLVSEEISKVIMSQSNQLGVFCHGFTYSGHPV
363 367 370
400
GAME12 ACAVALEALKIYKEKNITEVVNKLSPKFQEGLKAFIDSPIIGEIRGTGLVLSTEFVDNKSPNDPFFPPEW
GABA-T3 SCAVALETLKIYKERNIIEQVNRISPKFQEGLKAFSDSPIIGEIRGTGLLHGTEFTDNKSPNDPFFPPEW
GABA-T3 mut1 SCAVALETLKIYKERNIIEVVNRISPKFQEGLKAFIDSPIIGEIRGTGLVHSTEFTDNKSPNDPFFPPEW
GABA-T3 mut2 SCAVALETLKIYKERNIIEVVNRISPKFQEGLKAFIDSPIIGEIRGTGLVHSTEFTDNKSPNDPFFPPEW
GABA-T3 mut3 ACAVALEALKIYKERNITEVVNKLSPKFQEGLKAFIDSPIIGEIRGTGLVLSTEFVDNKSPNDPFFPPEW
410
426
440 442
470
GAME12 GVGTYFGSQCQKHGMLVSFSGDHVNMAPPFTLSLEELDEMISIYGKALKDTEKRVEELKSQKK----
GABA-T3 GIGAYFGARCEKHGVLVRVAGDNIMMSPPYILSLEEIDELIIKYGKALKDTENRVEELKSQKKIKSS
GABA-T3mut1 GIGAYFGARCEKHGVLVRVAGDNINMSPPYILSLEEIDELIIIYGKALKDTENRVEELKSQKKIKSS
GABA-T3 mut2 GIGAYFGARCQKHGVLVSFSGDNINMSPPYILSLEEIDELIIIYGKALKDTENRVEELKSQKKIKSS
GABA-T3 mut3 GVGTYFGSQCQKHGMLVSFSGDHVNMAPPFTLSLEEIDEMISIYGKALKDTENRVEELKSQKKIKSS
484
502

Supplementary Figure 29. Alignment of the amino acid sequences of tomato GAME12, GABA-T3 and the GABA-T3 mutants. Residues are numbered according to the amino acid alignment used to generate the GABA-T tree in **Extended Data Figure 1** for consistency. The sites corresponding to codons under strong diversifying selection described in **Supplementary Table 4** are labelled with red number. The individual mutated amino acids are highlighted in the colour corresponding to the color code of the GABA-T3 mutants. The residues in grey correspond to the N-terminal His-tag used for purification of the proteins. GABA-T3 mut1 contains 15 amino acid substitutions corresponding to the 14 residues found to be under strong diversifying selection at the GABA-T3/GABA-T2 and GABA-T2/GAME12 divergence points and a single additional mutation at residue 365. GABA-T3 mut2 contains 27 residue substitutions corresponding to the residues found to be under strong diversifying selection and residues within 8 Å of the docked-in 26-furostanol aldehyde (**3**) substrate. GABA-T3 mut3 contains 77 residue substitutions corresponding to all the mutated residues of GABA-T3 mut2 and additional mutations of residues within a 12 Å of the docked-in 26-furostanol aldehyde (**3**) substrate.

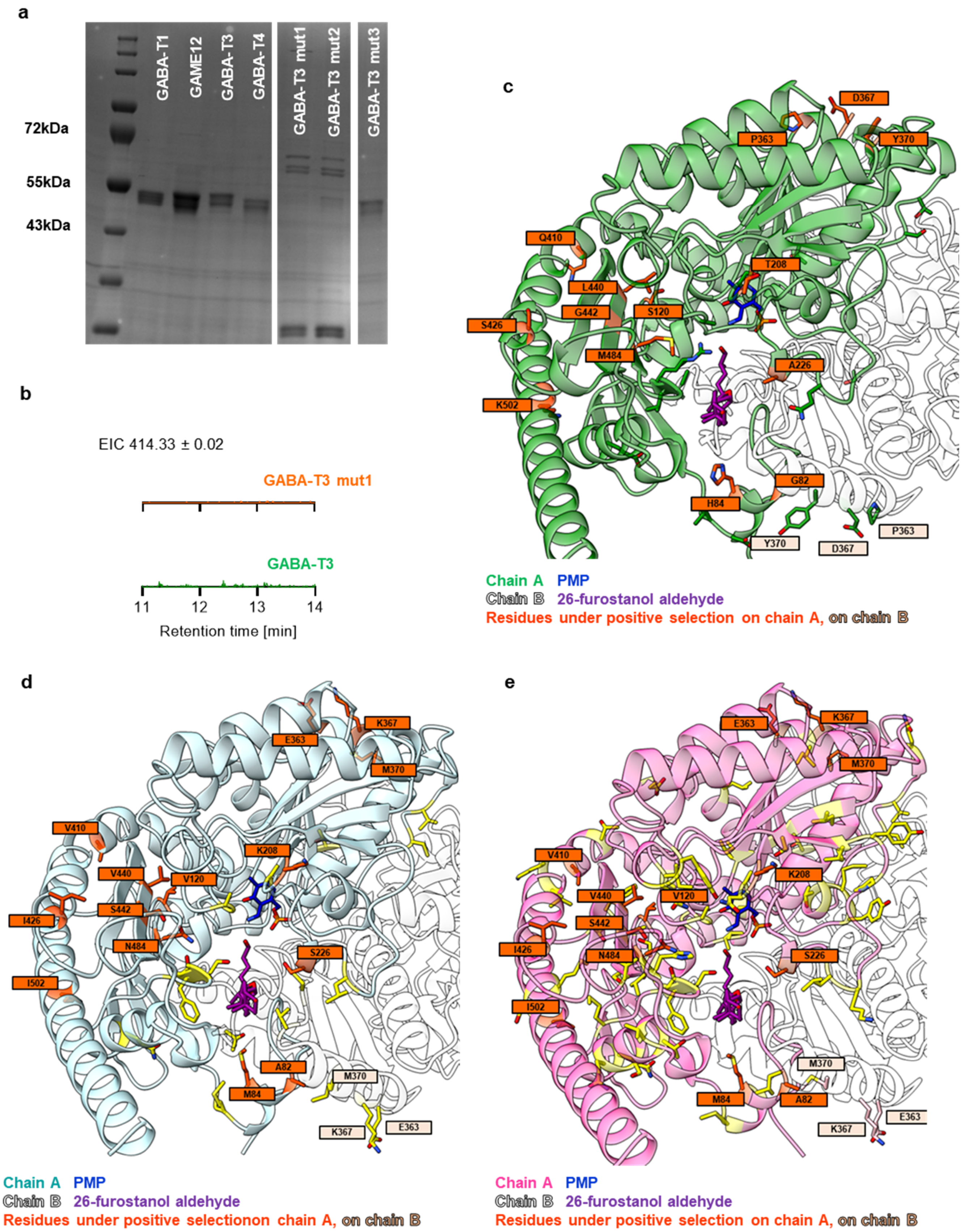
a
GABA-T1
GAME12
GABA-T3
GABA-T4
GABA-T3 mut1
GABA-T3 mut2
GABA-T3 mut3
72kDa
55kDa
43kDa
b
EIC 414.33 ± 0.02
GABA-T3 mut1
GABA-T3
11
12
13
14
Retention time [min]
c
D367
P363
Y370
Q410
T208
L440
G442
S120
S426
M484
A226
K502
G82
H84
Y370
D367
P363
Chain A PMP
Chain B 26-furostanol aldehyde
Residues under positive selection on chain A, on chain B
d
K367
E363
M370
V410
K208
V440
V120
S442
I426
N484
S226
I502
A82
M370
M84
K367
E363
Chain A PMP
Chain B 26-furostanol aldehyde
Residues under positive selectionon chain A, on chain B
e
E363
K367
M370
V410
K208
V440
V120
S442
N484
I426
S226
I502
M370
M84
A82
E363
K367
Chain A PMP
Chain B 26-furostanol aldehyde
Residues under positive selection on chain A, on chain B

Supplementary Figure 30. The architecture of the active sites of GABA-T3 and the GABA-T3 mutants and the results of mutant protein expression. a. SDS-PAGE analysis of the purified GABA-Ts and GABA-T3 mutants. The results for each GABA-T homologue and GABA-T3 mutant are representative of at least two independent experiments. An uncropped version of the image is presented in **Supplementary Figure 36**. **b.** EICs of the Rapidase and GAME4- coupled in vitro assays on the inactive GABA-T3 mut1 and GABA-T3 using protodioscin (**14**) as a substrate. The chromatogram y-axis max. is 1.6 x 10^3. **c.** Visualization of the AlphaFold-generated model of the GABA-T3 homodimer with 26-furostanol aldehyde (**3**) docked into the active site and the PMP co-factor modelled in. The residues under positive selection are labelled in orange boxes. **d.** Visualization of the AlphaFold-generated model of the GABA-T3 mut2 homodimer with 26-furostanol aldehyde (**3**) docked into the active site and the PMP co-factor modelled in. Residues identified to be under strong diversifying selection were substituted to the corresponding residues from GAME12 and are marked in orange. Other residues which were substituted are marked in yellow. **e.** Visualization of the AlphaFold-generated model of the GABA-T3 mut3 homodimer. Residues identified to be under strong diversifying selection are marked in orange. Other residues which were substituted are marked in yellow.

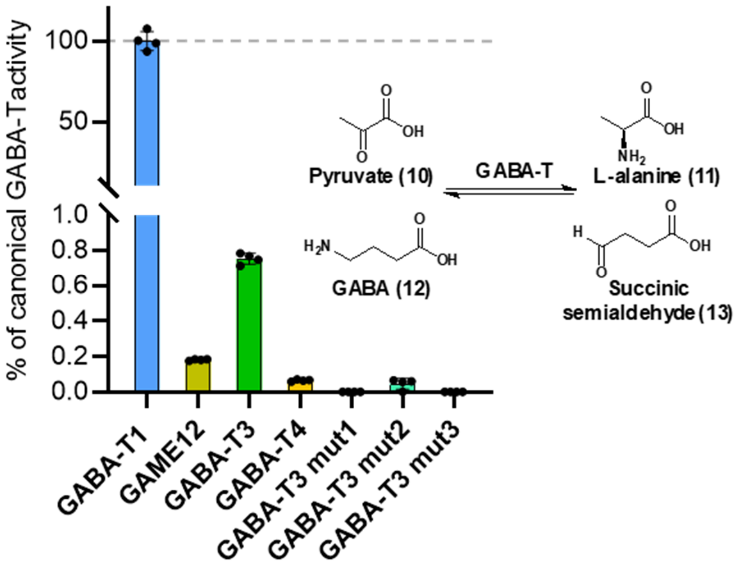

Supplementary Figure 31. Percentage activity of the GABA-Ts on the canonical substrate pyruvate (10), relative to the mean activity of GABA-T1. Each GABA-T assay was performed in four replicates (n = 4). The experiment was repeated twice with similar results. Individual data points are marked on the graph. Data are displayed as mean ± SD.

a

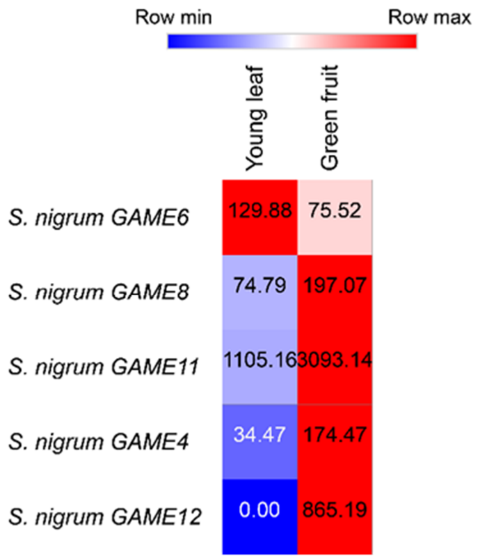

b

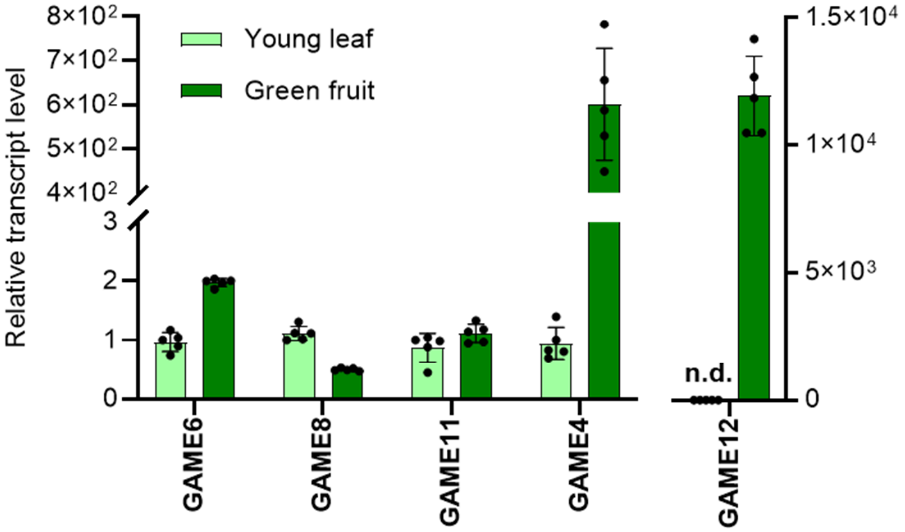

c

	Ef-1α	GAME6	GAME8	GAME11	GAME4	GAME12
			Average Ct			
Young leaf	19.39996	22.58755	20.36463	19.67973	35.78617	n.d.

Supplementary Figure 32. Analysis of the expression of *GAME* genes in wildtype *S. nigrum* tissues. a. Heatmap displaying the expression of the *S. nigrum GAME* homologues in young leaf and green fruit tissues**.** Normalized fragments per kilobase of transcript per million mapped reads (FPKM) values were used for visualization of expression. **b.** The results of qPCR analysis of the relative expression of *GAME* genes in *S. nigrum* wildtype plant tissues using the $2^{-\Delta\Delta Ct}$ method. *S. nigrum* elongation factor-1α (Ef-1α) was used as a housekeeping gene. *GAME12* expression in the *S. nigrum* young leaves was undetectable (n.d.), therefore the expression of *GAME12* in green fruit was calculated relative to the expression of *GAME4* in the young leaves. Individual data points derived from five (n=5) different biological replicates i.e., tissue samples from independent transgenic lines. Data are displayed as mean ± SD. **c.** The average Ct values obtained in the qPCR analysis of the expression of *GAME* genes in *S. nigrum.*

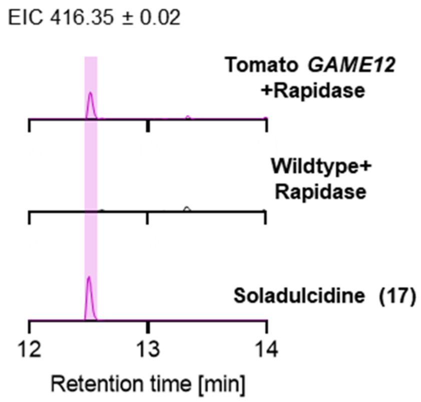

Supplementary Figure 33. UHPLC-MS data for the products of enzymatic hydrolysis of the extracts from the stable *S. nigrum* transgenic lines expressing tomato *GAME12*. Extracted ion chromatograms (EICs) of the products of enzymatic hydrolysis of leaf extracts from transgenic tomato *GAME1*2-expressing *S. nigrum* and wildtype *S. nigrum* lines and the soladulcidine (**17**) standard.

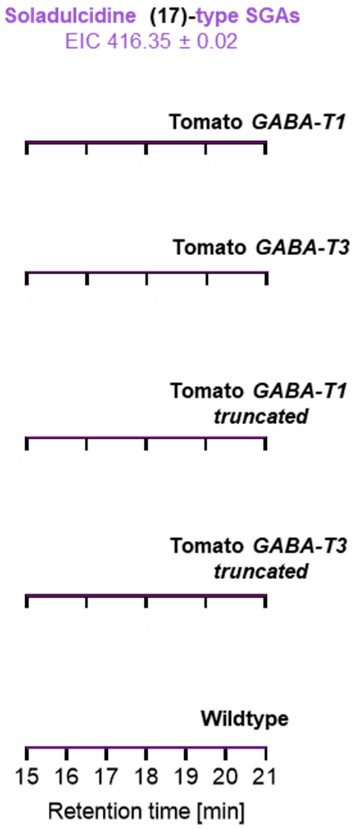

Supplementary Figure 34. UHPLC-MS data for the stable *S. nigrum* transgenic lines. a. EICs showing no accumulation of steroidal glycoalkaloids in the leaves of *GABA-T* overexpressing transgenic *S. nigrum* compared to wildtype *S. nigrum* plants.

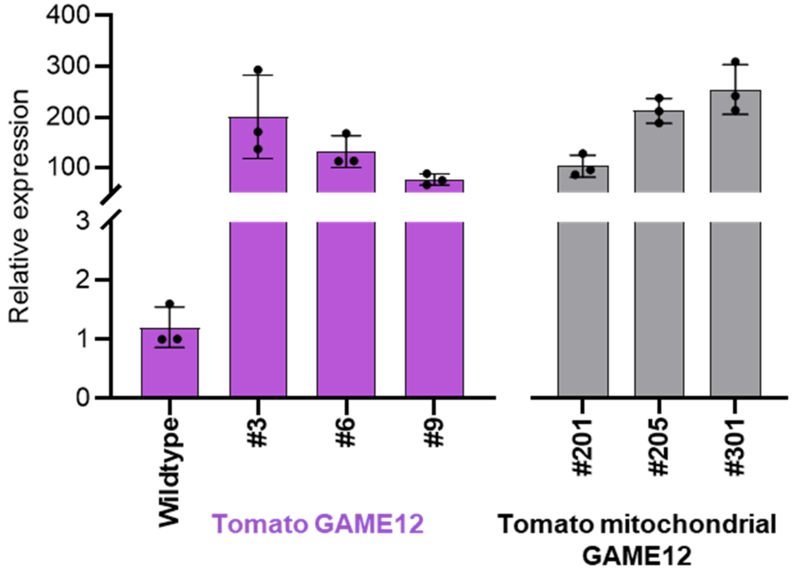

Supplementary Figure 35. Relative expression of GAME12 and mitochondrial-GAME12 version in transgenic *S. nigrum* lines as determined by qRT-PCR. The bar graphs represent data for three biological replicates (n = 3) obtained from independent transgenic lines (#3, #6, #9 for GAME12; #201, #205 and #301 for mitochondrial GAME12 version). Data are displayed as mean ± SD.

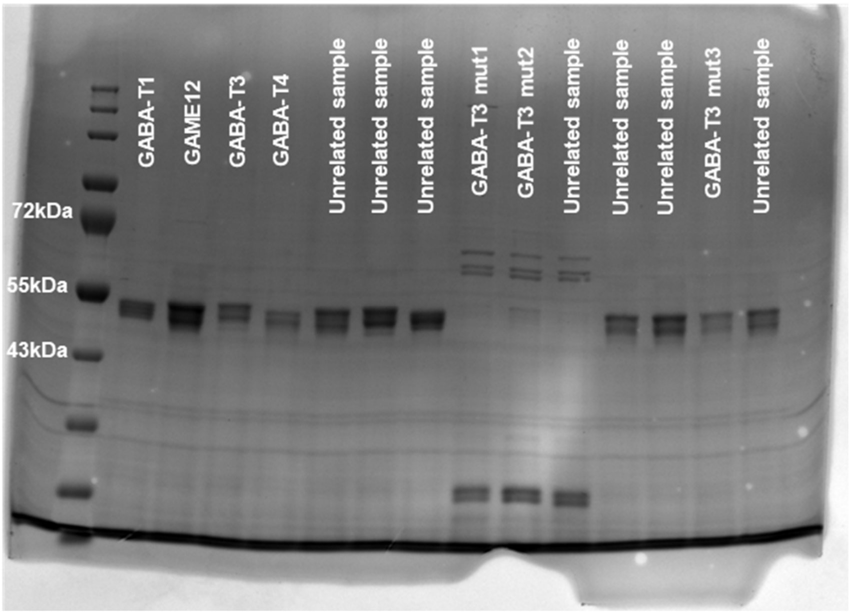

Supplementary Figure 36. The uncropped image of the SDS-PAGE analysis of the purified GABA-Ts and GABA-T3 mutants presented in Supplementary Figure 30a.

Supplementary Table 1. Amino acid sequence identity comparison between the four GABA-T homologues identified in tomato (*S. lycopersicum*).

	***S. lycopersicum* GABA-T1**	***S. lycopersicum* GABA-T2/GAME12**	***S. lycopersicum* GABA-T3**	***S. lycopersicum* GABA-T4**
***S. lycopersicum* GABA-T1**		76	80	78
***S. lycopersicum* GABA-T2/GAME12**	76		78	87
***S. lycopersicum* GABA-T3**	80	78		77
***S. lycopersicum* GABA-T4**	78	87	77	

Supplementary Table 2. ^{1}H- and ^{13}C-NMR data of uttroside B (16) in pyridine-d_5.

Aglycone part						Sugar moieties				
**pos.**	d_H[a] (ppm)	**mult.**	J_{HH} (Hz)	d_C[b] (ppm)		**pos.**	d_H[a] (ppm)	**mult.**	J_{HH} (Hz)	d_C[b] (ppm)
1a	0.80	*bdd*	12.7/11.9	37.65	Glc I :	1	4.84	*d*	7.7	105.4
1b	1.53	*bd*	12.7	37.65		2	4.04	*dd*	8.1/7.7	75.68
2a	2.05	*bd*	10.2	30.4		3	4.26	*m*[c]	-	79.1
2b	1.61	*bddd*	12.0/11.9/10.2	30.4		4	4.25	*m*[c]	-	72.2
3a	3.93	*bddd*	12.0/11.0/4.5	77.8		5	3.96	*m*	-	79.1
4a	1.79	*m*	-	35.3		6a	4.57	*dd*	11.8/2.0	63.3
4b	1.36	*bddd*	11.7/11.0/8.8	35.3		6b	4.41	*dd*	11.8/9.8	63.3
5a	0.90	*m*	-	45.1						
6a	1.11	*m*[c]	-	29.4	Gal :	1	4.91	*d*	7.52	102.9
6b	1.11	*m*[c]	-	29.4		2	4.44	*dd*	9.3/7.4	73.7
7a	0.78	*m*	-	32.88		3	4.14	*dd*	9.3/2.3	76.1
7b	1.52	*bd*	11.8	32.88		4	4.63	*d*	2.3	80.4
8b	1.41	*m*[c]	-	35.7		5	4.04	*dd*	8.6/5.3	75.8
9a	0.50	*m*	-	54.9		6a	4.72	*dd*	10.4/8.6	61.1
10	-	-	-	36.3		6b	4.25	*dd*	10.4/5.3	61.1
11a	1.41	*m*[c]	-	21.7						
11b	1.21	*bddd*	12.7/12.7/11.6	21.7	Glc II :	1	5.22	*d*	7.8	105.7
12a	1.07	*m*	-	40.7		2	4.46	*dd*	8.6/7.8	81.8
12b	1.71	*bd*	11.3	40.7		3	4.20	*dd*	8.8/8.6	87.1
13	-	-	-	41.6		4	3.85	*dd*	8.6/8.3	71.0
14a	1.04	*ddd*	12.5/12.4/6.0	56.8		5	3.91	*ddd*	8.3/8.3/1.5	78.1
15a	2.03	*ddd*	12.2/7.2/6.0	32.84		6a	4.55	*dd*	11.2/1.5	63.5
15b	1.43	*ddd*	12.5/12.2/7.2	32.84		6b	4.08	*dd*	11.2/8.3	63.5
16a	4.96	*ddd*	7.2/7.2/7.2	81.6						
17a	1.94	*dd*	7.2/6.6	64.5	Xyl :	1	5.27	*d*	7.7	105.4
18	0.89	*s*	-	17.2		2	3.99	*dd*	8.3/7.7	75.6
19	0.65	*s*	-	12.8		3	4.10	*dd*	8.6/8.3	79.1
20b	2.25	*dq*	6.6/6.8	41.2		4	4.13	*ddd*	9.7/8.6/4.8	71.2
21	1.35	*d*	6.8	16.9		5a	4.25	*dd*	11.0/4.8	67.8
22	-	-	-	111.1		5b	3.70	*dd*	11.0/9.7	67.8
23a	2.05	*m*	-	37.71						
23b	2.05	*m*	-	37.71	Glc III :	1	5.60	*d*	7.0	105.4
24a	2.06	*m*	-	28.9		2	4.10	*dd*	9.1/7.0	76.7
24b	1.70	*m*	-	28.9		3	4.13	*dd*	9.1/9.1	78.2
25	1.94	*m*	-	34.7		4	4.24	*dd*	9.1/8.4	71.5
26a	3.96	*dd*	9.1	75.68		5	3.94	*m*	-	79.3
26b	3.64	*dd*	9.1	75.68		6a	4.61	*dd*	12.0/1.5	62.9
27	1.00	*d*	6.6	17.94		6b	4.39	*dd*	12.0/4.3	62.9

[a] Measured at 500 MHz

[b] Measured at 125 MHz

[c] overlapped signals J unresolved

Supplementary Table 3. aBRSEL diversifying selection test. The *GABA-T* tree **(in Extended Data Figure 1a)** was fit to three models: a single ω rate class per branch model (1xω model), a full-adaptive model with multiple ω rate classes per branch (2xω model) and a null model where the branches are not allowed to have rate classes of ω>1. The Likelihood Ratio Test (LRT) is performed at each branch and compares the full-adaptive model to a null model. The *p*-values at each branch are corrected for multiple testing (using the Holm-Bonferroni correction). For a simplified tree showing position of test branches see **Supplementary Figure 25b**.

Branch	Description	LRT	Test *p*-value	Uncorrected *p*-value	1xω model	2xω model			
					ω	ω1	ω1 sites (%)	ω2	ω2 sites (%)
h	*GAME12*	15.3501	0.0014	0.0002	7.45	0.698	90	104	9.7
i	*GABA-T4*	11.4177	0.0091	0.0011	0.884	0	94	19	6.4
e	*GABA-T2*	8.773	0.0304	0.0043	1	0	86	9.53	14
d	*GABA-T3*	4.4161	0.2406	0.0401	3.39	1	99	224	0.79
a	Solanaceae	0	0	0	0.078	0.076	100	0	0
b	*GABA-T1*	0	0	0	0.103	0.11	100	0	0
c	*GABA-T2/T3*	0	0	0	0.151	0.144	100	0	0
e	*GABA-T2 N. benthamiana*	0	0	0	0.164	0.144	100	0	0
f	*GABA-T2 Physalis/Capsicum*	0	0	0	0.311	0.282	100	0	0

Supplementary Table 4. Site-specific diversifying selection tests. The *GABA-T* tree (**Extended Data Figure 1a, Supplementary Figure 25**) was subjected to BUSTED (Branch-site Unrestricted Statistical Test for Episodic Diversification) and MEME (Mixed Effects Model of Evolution) tests to detect specific codons under selection across branches of interest for mutation analysis. BUSTED found evidence of gene-wide episodic diversifying selection (p = 0.0023), with 15 codons with positive selection evidence ratio > 5. Branch-site positive selection test across *a priori* defined branches (**Supplementary Figure 25b**) of each codon was determined using empirical bayes factors (**Supplementary Figure 27b**, **Supplementary Figure 28**). In addition, MEME was used to identify sites under episodic positive/diversifying selection across a proportion of branches (p < 0.05). These sites were then assessed using branch-site Empirical Bayes Factors data to identify sites under selection with non-synonymous mutations on branches of interest not previously identified using BUSTED. Codons not used in mutation analysis (i.e. truncated N-terminal region or amino acids the same in GABA-T3/GAME12) not shown.

Codon	BUSTED				MEME	Codon/amino acid	
	Positive selection evidence ratio	Branch	Posterior Probability	Empirical Bayes Factor	*p*-value	GABA-T3	GAME12
82	1.11	94	0.142	14.7	0.01	GGA/G	GCA/A
84	19.223	94	0.964	2407.9	0.3	CAT/H	ATG/M
120	5.207	104	0.759	281.1	0.39	TCT/S	GTA/V
208	9.673	104	0.917	985.4	0	ACA/T	AAA/K
226	19.884	89	0.936	1300.2	0.15	GCA/A	TCA/S
363	100.442	104	0.994	15400.9	0.02	CCT/P	GAA/E
367	5.014	88	0.826	423.3	0.62	GAT/D	AAG/K
370	19.814	94	0.966	2499.2	0.01	TAT/Y	ATG/M
410	8.637	82	0.918	993.9	0.12	CAA/Q	GTA/V
426	8.567	94	0.915	963.2	0.67	TCT/S	ATA/I
440	9.08	101	0.860	549.2	0.08	CTA/L	GTA/V
442	7.906	82	0.898	785.9	0.15	GGT/G	TCT/S
484	10.868	82	0.936	1306.9	0.06	ATG/M	AAT/N
502	1.216	59	0.285	35.5	0.05	AAA/K	ATA/I

Supplementary Table 5. The putative fragmentation patterns of soladulcidine (17)-type SGAs produced *de novo* in the leaves of transgenic *S. nigrum* plants.

Product	Retention time [min]	*m/z* molecular ion	*m/z* fragment ions	Molecular formula	ΔppM
D	16.9	1150.6009 $[M+H]^+$	1018.5576 $[M\text{-}Xyl+H]^+$<br>724.4622$[M\text{-}Xyl\text{-}Hex\text{-}Xyl+H]^+$<br>578.4044 $[M\text{-}Xyl\text{-}Hex\text{-}Xyl\text{-}Hex+H]^+$<br>416.3523 $[M\text{-}Xyl\text{-}Hex\text{-}Xyl\text{-}Hex\text{-}Hex+H]^+$	$C_{55}H_{92}NO_{24}$	4.3
E	17.8	1034.5536 $[M+H]^+$	902.5060 $[M\ \text{-}Xyl+H]^+$<br>578.4053 $[M\ \text{-}Hex\text{-}Hex\text{-}Xyl+H]^+$<br>416.3528 $[M\ \text{-}Hex\text{-}Hex\text{-}Xyl\text{-}Hex+H]^+$	$C_{50}H_{84}NO_{21}$	1.7
F	18.5	1150.5947 $[M+H]^+$	1004.5444 $[M\text{-}Xyl+H]^+$<br>856. 5054 $[M\text{-}Xyl\text{-}Hex+H]^+$<br>724.4620 $[M\text{-}Xyl\text{-}Hex\text{-}Xyl+H]^+$<br>578.4044 $[M\text{-}Xyl\text{-}Hex\text{-}Xyl\text{-}Rha+H]^+$<br>416.3524 $[M\text{-}Xyl\text{-}Hex\text{-}Xyl\text{-}Rha\text{-}Hex+H]^+$	$C_{55}H_{92}NO_{24}$	0.3

Supplementary Table 6. The accession numbers of the genomes used for the phylogenomics and synteny analysis of GABA-T homologues.

Species	Accession	Database	Reference
I. triloba	NSP323_triloba_V3	http://sweetpotato.plantbiology.msu.edu	5
N. benthamiana	NbQld183.v103	https://www.nbenth.com/	6
P. floridana	GWHANUX00000000	https://ngdc.cncb.ac.cn/	7
C. annuum	GCA_002878395.3	NCBI BioProject PRJNA376668	8
S. tuberosum	DM1–3 516 R44	http://spuddb.uga.edu/dm_v6_1_download.shtml	9
S. lycopersicum	Tomato Genome version SL4.0 and Annotation ITAG4.0	https://solgenomics.net/ftp/tomato_genome/annotation/ITAG4.0_release/	10
S. americanum	GCA_030407365.1 (SP1102)	NCBI BioProject PRJNA845062	11
P. inflata	draft genome sequence v1.0.1	https://solgenomics.sgn.cornell.edu/organism/Petunia_inflata/genome	12
S. dulcumara	GCA_947179165.2	NCBI BioProject PRJEB56942	13

Supplementary Table 7. Sequences of primers used in this study.

Primer ID	Primer sequence
GAME6 3Ω1 fwd	TTTATGAATTTTGCAGCTCGA ATGGCTATTGCAATTTTCATAGCTTTGG
GAME6 3Ω1 rev	GACAACCACAACAAGCACCGTCATTCTAGCTTTCGAACAATCATAGG
GAME8 3Ω1 fwd	TTTATGAATTTTGCAGCTCGAATGGCAATTGTTACAGTAATTGGTGCAAC
GAME8 3Ω1 rev	GACAACCACAACAAGCACCGTCAGAGCTTGTTGAGGATTATCTGAGC
GAME11 3Ω1 fwd	TTTATGAATTTTGCAGCTCGAATGGCGGATCTTCTCTCGAACTGGTC
GAME11 3Ω1 rev	GACAACCACAACAAGCACCGTTAATTAGCTTCTGTTTTGAAGGGCTCC
GAME4 3Ω1 fwd	TTTATGAATTTTGCAGCTCGAATGGATTTCTACAATTTAGCCTTGTTCTTC
GAME4 3Ω1 rev	GACAACCACAACAAGCACCGTTAATTACCATTACTAGAGATCGATAGCTTC
GAME12 3Ω1 fwd	TTTATGAATTTTGCAGCTCGAATGGCCAAGACTAATGGATTTATGGG
GAME12 3Ω1 rev	GACAACCACAACAAGCACCGTTACTTCTTCTGAGACTTCAATTCTTCC
3Ω1 sequencing fwd	TCTTGTGTTTTAGGTTGTTTTAATCCT
3Ω1 sequencing rev	CAACCACCACAATGGGCAAC
GABA-T1 : RFP fwd	TAGAGCGATGAGTGGTCTCGAATGGCCAAGATTTCTCGTCTTTTTG
GABA-T1 : RFP rev	ACTAGTCGCGATGAGTGGTCTCACTGCCTTCTGAGACTTGAGTTCTTCTAC
GABA-T1 Truncated : RFP fwd	GATGAGTGGTCTCGAATGGGAACTGATAATAAAGGGTTCAA
GABA-T1 Truncated : RFP rev	GATGAGTGGTCTCACTGCCTTCTGAGACTTGAGTTCTTCTAC
GAME12 : RFP fwd	TAGAGCGATGAGTGGTCTCGAATGATGGCCAAGACTAATGGATTTATGG
GAME12 : RFP rev	ACTAGTCGCGATGAGTGGTCTCACTGCCTTCTTCTGAGACTTCAATTCTTCC
GABA-T3 : RFP fwd	TAGAGCGATGAGTGGTCTCGAATGGCCAAGATTACTAGTTTAATTGGA
GABA-T3 : RFP rev	AGTCGCGATGAGTGGTCTCACTGCTGAGCTTTTTATCTTCTTCTGAGACTTG
GABA-T3 Truncated : RFP fwd	GGTCTCGAATGAAGGGGCATGATATGTTGG
GABA-T3 Truncated : RFP rev	GATGAGTGGTCTCACTGCTGAGCTTTTTATCTTCTTCTGAGACTT
GABA-T4 : RFP fwd	TAGAGCGATGAGTGGTCTCGAATGGGAGAGGTTAATGGATTTATG
GABA-T4 : RFP rev	AGTCGCGATGAGTGGTCTCACTGCCTTCTTCATAGCCTTAAGTTCTTCTAC
GAME4 pESC fwd	GCGAAGAATTGTTAATTAAGATTACCATTACTAGAGATCGATAGCT
GAME4 pESC rev	CACTAAAGGGCGGCCACCATGGATTTCTACAATTTAGCCT
GABA-T1 pOPINF fwd	AAGTTCTGTTTCAGGGTCCGGGAACTGATAATAAAGGGTTC
GABA-T1 pOPINF rev	CAAACTGGTCTAGAAAGCTTTTACTTCTGAGACTTGAGTTCTTCTACTC
GAME12 pOPINF fwd	AAGTTCTGTTTCAGGGTCCGGCCAAGACTAATGGATTTATGGG
GAME12 pOPINF rev	CAAACTGGTCTAGAAAGCTTTTACTTCTTCTGAGACTTCAATTCTTCC
GABA-T3 pOPINF fwd	AAGTTCTGTTTCAGGGTATGAAGGGGCATGATATGTTGG
GABA-T3 pOPINF rev	AACTGGTCTAGAAAGCTTCATGAGCTTTTTATCTTCTTCTGAGAC
GABA-T4 pOPINF fwd	AAGTTCTGTTTCAGGGCATGGGAGAGGTTAATGGATTTATGGG
GABA-T4 pOPINF rev	AACTGGTCTAGAAAGCTTTACTTCTTCATAGCCTTAAGTTCT
pOPINF sequencing fwd	TAATACGACTCACTATAGGG
pOPINF sequencing rev	TAGCCAGAAGTCAGATGCT
S. nigrum GAME12 qPCR fwd	ATGGCCAAGACTACTAATGGATTTAC
S. nigrum GAME12 qPCR rev	AAGACGAGGCTCATTTCCTCC
S. nigrum EF-1α qPCR fwd	TTTCACTGCCCAGGTCATCA
S. nigrum EF-1α qPCR rev	CAAACTTGACAGCAATGTGGGA
S. nigrum GAME6 qPCR fwd	CAATTCCAGCAGGTGCACAAC
S. nigrum GAME6 qPCR rev	TCTGGGCGGAAAATTAGTGCG
S. nigrum GAME8 qPCR fwd	TGAGTTGGATGTGGCGGAC

S. nigrum GAME8 qPCR rev	AGCTCACACTGCTCCTTTTG
S. nigrum GAME11 qPCR fwd	AACCACGGAGTACCGGAAAA
S. nigrum GAME11 qPCR rev	GTACAGTGTAGCAGCACCTCT
S. nigrum GAME4 qPCR fwd	AATACATGGGGATTATTGCAAAGGA
S. nigrum GAME4 qPCR rev	GTTCATAATTGCCTTGGTAGCCA
GABA-T1 mitochondrial fwd1	AAAAA ATGGCCAAGATTTCTCGTCTTTTTGG
GABA-T1 mitochondrial rev1	CCCATAAATCCATTAGTCTTGGCCAT AGAAATATCATTTTCCAAACATGCC
GABA-T1 mitochondrial fwd2	GGCATGTTTGGAAAATGATATTTCTATGGCCAAGACTAATGGATTTATGGG
GABA-T1 mitochondrial rev2	AAAAA TTACTTCTTCTGAGACTTCAATTCTTCC
GABA-T1 mitochondrial qPCR fwd	ATGGCCAAGACTACTAATGGATTTAC
GABA-T1 mitochondrial qPCR rev	AAGACGAGGCTCATTTCCTCC

Supplementary References

1. Clark, S. M., Di Leo, R., Van Cauwenberghe, O. R., Mullen, R. T. & Shelp, B. J. Subcellular localization and expression of multiple tomato γ-aminobutyrate transaminases that utilize both pyruvate and glyoxylate. *J. Exp. Bot.* **60**, 3255–3267 (2009).
2. Sievers, F. & Higgins, D. G. Clustal Omega for making accurate alignments of many protein sequences. *Protein Sci.* **27**, 135–145 (2018).
3. Waterhouse, A. M., Procter, J. B., Martin, D. M. A., Clamp, M. & Barton, G. J. Jalview Version 2—a multiple sequence alignment editor and analysis workbench. *Bioinformatics* **25**, 1189–1191 (2009).
4. Fei, Z. *et al.* Tomato Functional Genomics Database: a comprehensive resource and analysis package for tomato functional genomics. *Nucleic Acids Res.* **39**, D1156–D1163 (2011).
5. Wu, S. *et al.* Genome sequences of two diploid wild relatives of cultivated sweetpotato reveal targets for genetic improvement. *Nat. Commun.* **9**, 4580 (2018).
6. Ranawaka, B. *et al.* A multi-omic *Nicotiana benthamiana* resource for fundamental research and biotechnology. *Nat. Plants* **9**, 1558–1571 (2023).
7. Lu, J. *et al.* The *Physalis floridana* genome provides insights into the biochemical and morphological evolution of *Physalis* fruits. *Hortic. Res.* **8**, 1–19 (2021).
8. Hulse-Kemp, A. M. *et al.* Reference quality assembly of the 3.5-Gb genome of *Capsicum annuum* from a single linked-read library. *Hortic. Res.* **5**, 4 (2018).
9. Pham, G. M. *et al.* Construction of a chromosome-scale long-read reference genome assembly for potato. *GigaScience* **9**, giaa100 (2020).
10. Hosmani, P. S. *et al.* An improved *de novo* assembly and annotation of the tomato reference genome using single-molecule sequencing, Hi-C proximity ligation and optical maps. 767764 Preprint at https://doi.org/10.1101/767764 (2019).

11. Lin, X. *et al. Solanum americanum* genome-assisted discovery of immune receptors that detect potato late blight pathogen effectors. *Nat. Genet.* **55**, 1579–1588 (2023).

12. Bombarely, A. *et al.* Insight into the evolution of the Solanaceae from the parental genomes of *Petunia hybrida*. *Nat. Plants* **2**, 1–9 (2016).

13. Christenhusz, M. J. M. The genome sequence of bittersweet, *Solanum dulcamara* L. (Solanaceae). *Wellcome Open Res.* **8**, 409 (2023).
